# Inert Catalytic Sites Unlocked by Micropollutants for Rapid Water Decontamination with Near‐Complete Chemical Utilization

**DOI:** 10.1002/adma.73103

**Published:** 2026-04-19

**Authors:** Yu‐Hang Li, Mingyi Liu, Yuanhao Li, Yaran Bai, Fu‐Xue Wang, Xiaoyi Hu, Xiaoguang Duan, Haodong Ji

**Affiliations:** ^1^ Eco‐environment and Resource Efficiency Research Laboratory, School of Environment and Energy Peking University Shenzhen Graduate School Shenzhen Guangdong P. R. China; ^2^ Department of Chemistry University of Pittsburgh Pennsylvania USA; ^3^ Laboratory of Agro‐Forestry Environmental Processes and Ecological Regulation of Hainan Province, School of Environmental Science and Engineering Hainan University Hainan P. R. China; ^4^ School of Chemical Engineering Adelaide University Adelaide South Australia Australia

**Keywords:** charge‐confined PKU‐24, dipole moment, peroxymonosulfate activation, pollutant degradation, singlet oxygen

## Abstract

The utilization efficiencies of peroxide and the evolved reactive species are typically low in advanced oxidation processes due to their self‐quenching or scavenging by background factors in water matrices. Here, we design a new Co‐based metal‐organic framework (PKU‐24) with a six‐coordinate structure that induces strong electron localization and suppresses the redox activity of Co sites, thereby blocking the electron‐transfer pathway. Interestingly, electron‐rich pollutants with oriented dipole moments can act as molecular “switches”, effectively turning on PKU‐24 activity for peroxymonosulfate (PMS) activation. Computations reveal that the dipole moment of pollutants is the decisive descriptor governing the behavior of organic contaminants in activating inert Co centers and reopening the electron‐transfer channel to mediate SO_5_^•−^ toward selective singlet oxygen generation. Additionally, this work develops a large‐scale synthesis method for PKU‐24 with a single synthesis yield of ∼0.55 kg (costing ∼0.52 USD g^−1^) and achieves long‐term, efficient, continuous treatment of organic wastewater. This work introduces a new principle for the design of pollutant‐sensitive Fenton‐like catalysts to achieve effective organic elimination while significantly reducing peroxide consumption.

## Graphical abstract

Micropollutants can act as molecular triggers that activate a newly designed and chemically inert Fenton‐like PKU‐24 catalyst for on‐demand singlet oxygen generation from peroxymonosulfate (PMS), enabling nearly complete peroxide utilization and selective micropollutant removal from complex real‐world wastewater. Overall, this study is not only groundbreaking in the exploration of advanced oxidation mechanisms but also demonstrates significant environmental applicability.

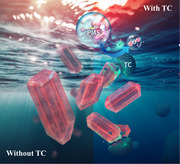

## 1 Introduction

The increasing prevalence of emerging contaminants has intensified global concerns over water pollution. Heterogeneous Fenton‐like reactions have shown promising practical potential, as the generated reactive species can effectively degrade contaminants and even achieve complete mineralization [1, 2, 3]. Conventionally, reactive oxygen species (ROS) are generated in advanced oxidation processes (AOPs) via electron transfer between catalytic sites and oxidants such as hydrogen peroxide, persulfates, or peracetic acid [4, 5, 6], thereby enabling radical and/or nonradical pathways [7, 8, 9]. In most reported AOP systems, catalysts act as either electron donors or acceptors, whereas peroxide activation is largely independent of the presence of contaminants [10, 11]. Consequently, Fenton‐like processes continue even after pollutants are completely removed, along with significant ROS quenching in complex water matrices [12], leading to excessive oxidant consumption. Designing catalysts whose activity can be switched on or off by microcontaminants would therefore represent a major advancement, enabling precise control of reaction initiation and termination and maximizing the utilization of oxidants.

To achieve controllable reactivity, the catalyst should possess an inert, charge‐confined structure that suppresses electron transfer between catalytic sites and oxidants, which can be achieved through atomic‐level coordination regulation. Metal‐organic frameworks (MOFs), with their tunable structures and well‐defined coordination environments, are particularly suitable: their specific coordination modes effectively prevent metal aggregation, comparable to single‐atom catalysts [13, 14, 15], while offering high structural adjustability [16, 17]. Typically, transition metals in high‐spin states, such as Co(II), Fe(II), or Ni(I), readily donate electrons to oxidants, thereby generating ROS to attack organic pollutants [18, 19]. Increasing the coordination number or introducing electronegative heteroatoms around the metal center can instead regulate orbital splitting and spin state, confining charge transfer and weakening oxidant‐catalyst interactions. This design strategy enhances electron localization, suppressing the intrinsic redox activity of catalytic sites. Intriguingly, adsorption of electron‐rich pollutants may perturb this confinement. Recent studies show that such pollutants can directly donate electrons to transition‐metal sites, increasing their electron density near the Fermi level [20, 21].

Here, we design and synthesize a new cobalt‐based MOF (Co‐MOF, named PKU‐24) via a simple hydrothermal route, where 2‐hydroxyterephthalic acid and 4,4‐bipyridine coordinate with Co atoms to form a six‐coordinate octahedral structure. The carboxyl oxygens, with strong electron‐withdrawing ability, induce a strong charge‐confined effect that suppresses Co redox activity. Combined characterizations and density functional theory (DFT) calculations confirm that this effect weakens peroxymonosulfate (PMS)‐Co interactions, preventing anti‐bonding orbitals from crossing the Fermi level and thereby blocking the electron transfer channel. Remarkably, adsorption of electron‐rich pollutants with oriented dipole moments such as tetracycline (TC) reconfigures the electronic density of Co sites, elevating the *d*‐band center and spin state, and reopening the electron transport channel, as directly observed by in situ X‐ray photoelectron spectroscopy (XPS). We identify that pollutants act as molecular “switches” that regulate catalytic initiation and termination when electron‐rich groups are located near the origin of the dipole moment (defined from negative charge to positive charge). The magnitude of the dipole moment shows a positive correlation with the catalytic degradation rate. This switchable process achieves near 100% PMS utilization to convert into selective singlet oxygen (^1^O_2_), as ROS forms only in the presence of pollutants. Additionally, we develop synthesis methods for PKU‐24 in kilograms, with a single synthesis yield of ∼0.55 kg (costing 0.52 USD g^−1^), demonstrating that PKU‐24/PMS has significant environmental promise from both operational and life‐cycle perspectives. Overall, our findings explain how organic contaminants regulate electron transport between catalysts and oxidants, offering a theoretical basis for developing charge‐confined catalysts with unprecedented persulfate utilization and remediation efficiencies.

## 2 Results and Discussion

### 2.1 Characterizations of PKU‐24

**FIGURE 1:**
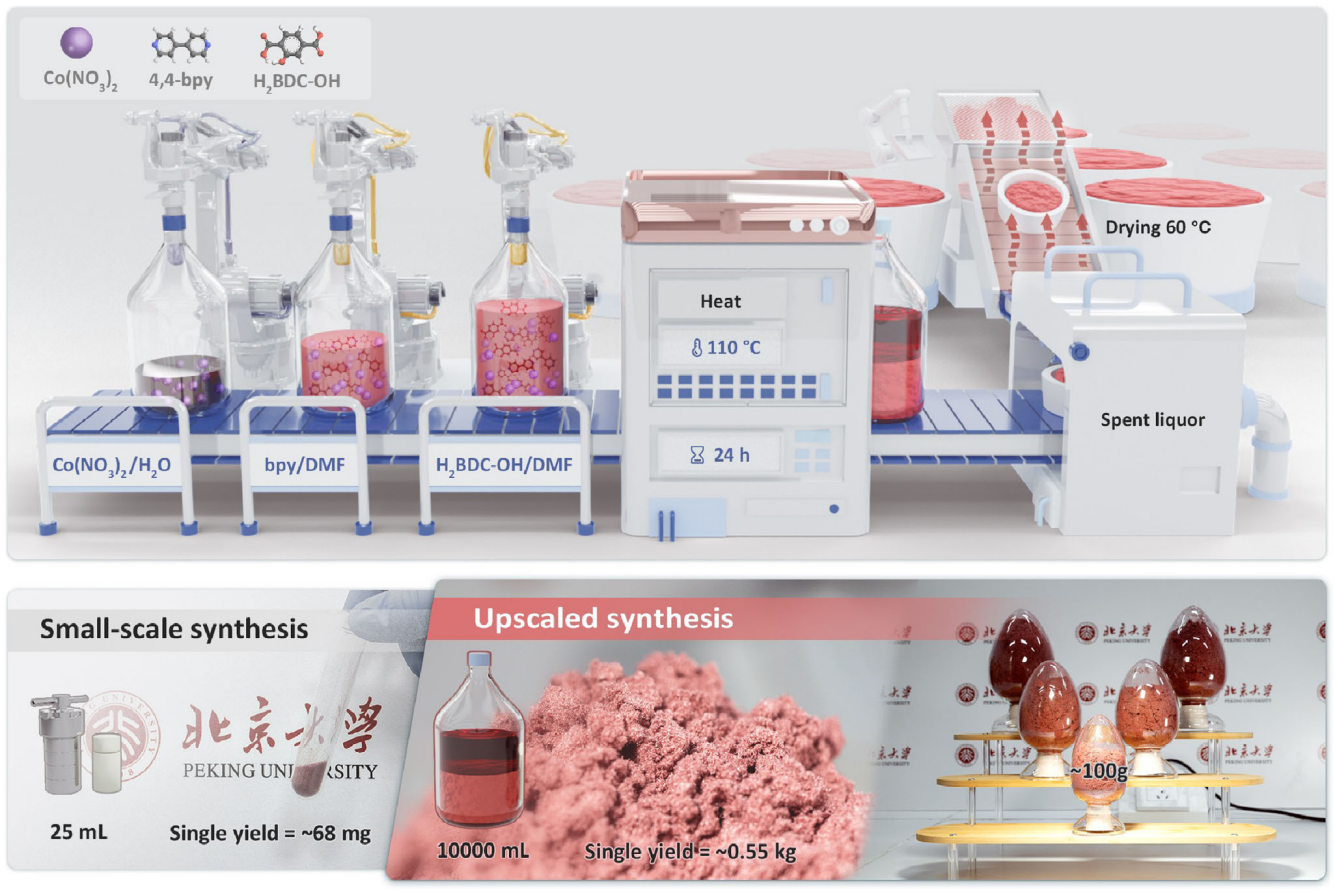
Fabrication of PKU‐24. Schematic diagram of synthesis processes of small‐scale and upscaled PKU‐24.

**FIGURE 2:**
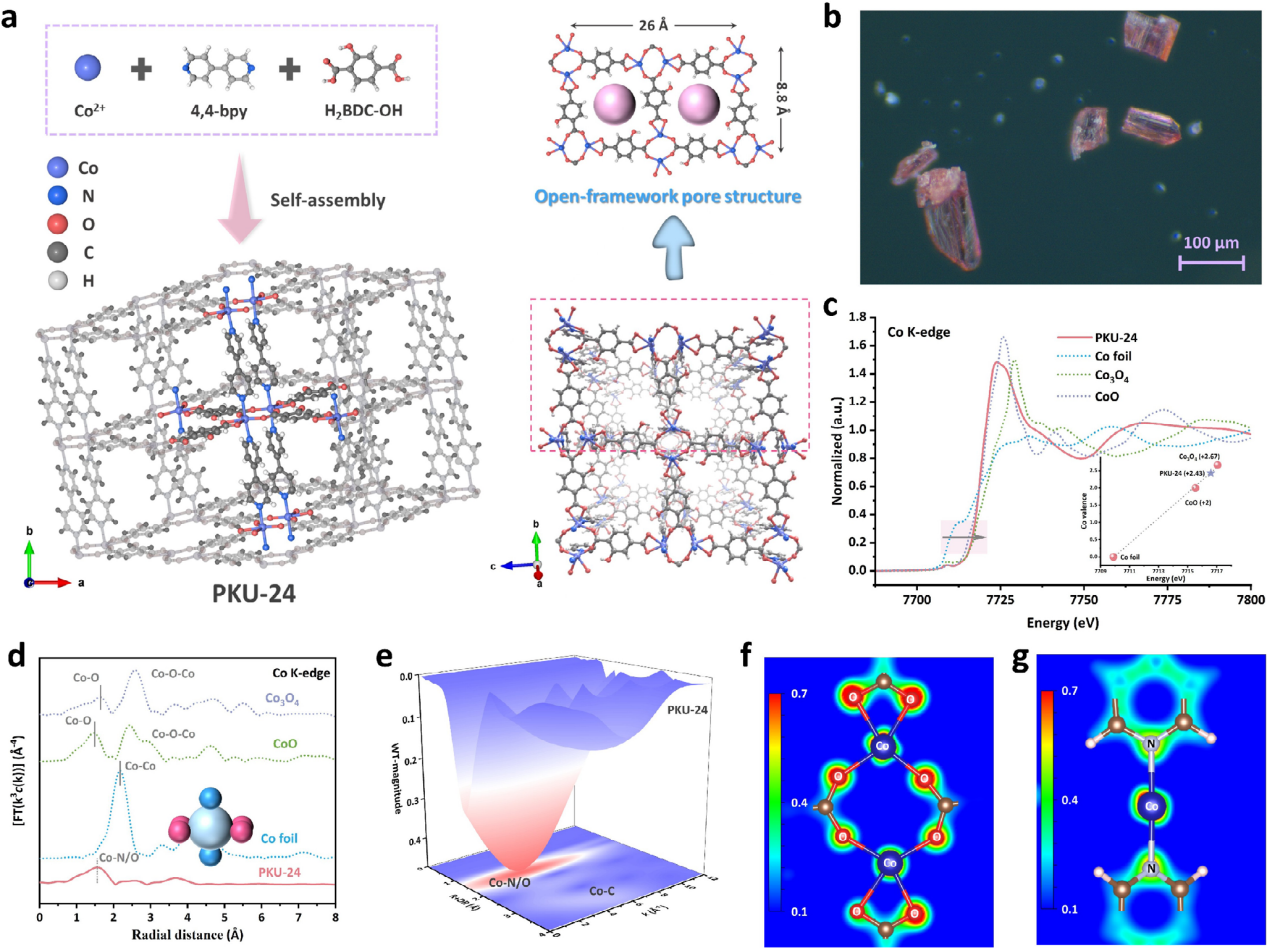
Characterizations of PKU‐24. (a) Crystal structure and open‐framework pore structure of PKU‐24. (b) Optical microscope image of PKU‐24 crystal. (c) XANES spectra (insert: the Co valence of different samples), (d) EXAFS spectra, and (e) WT plot of Co K‐edge in PKU‐24. (f,g) 2D valence electron density color‐filled map of PKU‐24.

The schematic diagrams of the fabrication processes for small‐scale and upscaled PKU‐24 are presented in Figure 1 and Video S1. We could realize the fabrication of large‐scale PKU‐24 in kilograms with the single production output of ∼0.55 kg. The crystal structure and data of PKU‐24 were analyzed by single‐crystal X‐ray diffraction (XRD), as exhibited in Figure 2a and Table S2. In a PKU‐24 unit cell, one Co atom with six coordination was linked by four O atoms from 2‐hydroxyterephthalic acid (H_2_BDC‐OH) and two N atoms from 4,4‐bipyridine (4,4‐bpy). Powder XRD patterns (Figure S1) of pure PKU‐24 produced by small‐scale and large‐scale synthesis all fitted well with simulated PKU‐24 from the crystal data (CCDC: 2424707). Optical microscope (Figure 2b) and scanning electron microscopy (SEM) images (Figure S2) showed that pure PKU‐24 presented the pink bulk crystal with a size of about 50.0 µm. The corresponding elemental mapping (Figure S3) showed uniform distributions of Co, C, N, and O. The N_2_ adsorption‐desorption isotherm (Figure S4) exhibited that PKU‐24 owned a large specific surface area (2.11 m^2^ g^−1^), and the type I isotherm indicated that the microporous structure (∼2.49 nm) was dominant in PKU‐24. The thermogravimetric analysis curve illustrated the good thermal stability of PKU‐24 (Figure S5).

The Co K‐edge X‐ray absorption near‐edge structure (XANES) illustrated that the chemical valence state of Co in PKU‐24 was located between +2 and +8/3 (Figure 2c), indicating that the electronic properties of Co sites changed when two ligands coordinated with Co(II). The extended X‐ray absorption fine structure (EXAFS) indicated that the coordination number of Co atoms was ∼6, which could be identified as Co─N/O bonds (Figure 2d; Figure S6 and Table S3). Additionally, no vacancy‐related signals were observed in the electron paramagnetic resonance (EPR) spectrum of PKU‐24 (Figure S7), further confirming the six‐coordinate structure of the Co atoms. The average bond length (Co─N/O, 2.06 Å) from EXAFS analysis was similar to the Co─N bond (2.06 Å) and Co─O bond (2.06 Å) presented by the results of single crystal XRD. Wavelet transform (WT) contour plots of PKU‐24 displayed the long distance between two Co atoms in PKU‐24, which was difficult to observe in the contour plot (Figure 2e). Moreover, the Co─N/O bonds in the WT contour plot differed from those in CoO and Co_3_O_4_ (Figure S8). XPS O 1*s* spectra (Figure S9a) showed that the peaks of hydroxyl oxygen (─OH, 532.74 eV) and carboxyl oxygen (O─C═O, 531.6 eV) for pristine PKU‐24 shifted to lower binding energies compared with those for H_2_BDC‐OH (─OH: 532.36 eV; O─C═O: 531.24 eV), signifying that partial electrons were transferred from the Co sites to hydroxyl oxygen and carboxyl oxygen. Meanwhile, N 1*s* spectra analysis (Figure S9b) showed that 4,4‐bpy, as the electrophilic ligand in PKU‐24, decreased the charge of Co sites. In addition, the Bader charge (Figure 2f,g; Figure S10) of all O and N atoms in the PKU‐24 unit cell decreased compared to pristine ligands, and the O atoms possessed better electronic local effect than that of N atoms. Electrostatic potential spectra of ligands and the PKU‐24 unit cell (Figure S11) displayed that abundant electron‐dissipating clouds were aggregated around the Co sites, confirming the electron‐withdrawing capacities of organic ligands. Therefore, although Co(II) was one of the precursors of PKU‐24 synthesis, the content of Co(II) was only about 16.7% as presented by the XPS Co 2*p* spectrogram (Figure S12).

### 2.2 Fenton‐Like Activity Test and Oxidation Mechanism Identification

**FIGURE 3:**
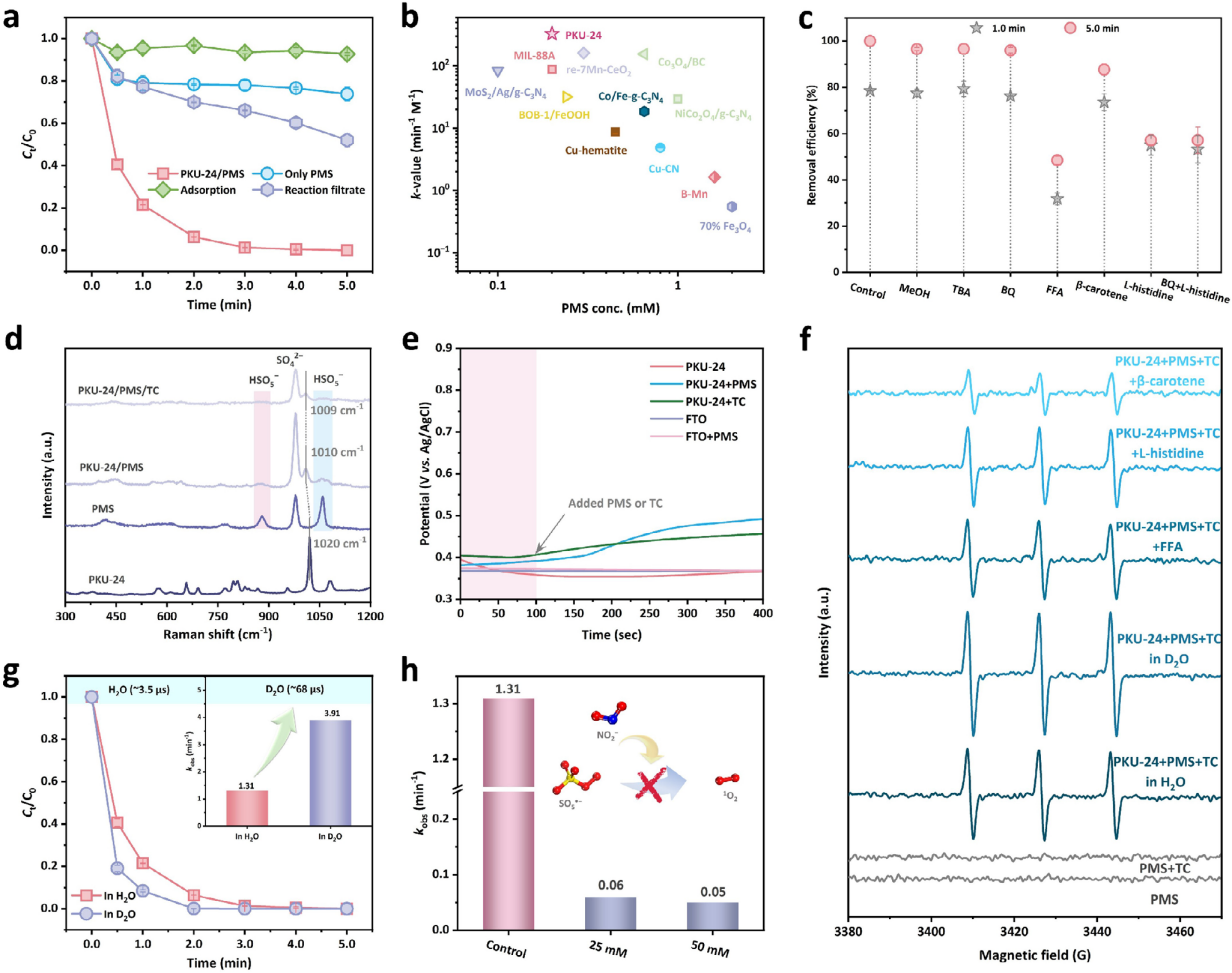
Fenton‐like activity test and ROS identification. (a) The Fenton‐like reaction activities test over different systems. (b) Comparison of the *k*_obs_ of TC elimination by the state‐of‐the‐art materials activating PMS systems (the detailed information was offered in Table S4). (c) Influences of various quenchers on TC removal efficiencies. (d) In situ Raman spectra of different systems. (e) Open‐circuit potential curves in different systems. (f) EPR spectra using TEMP as the trapping agent under different conditions. (g) Influence of D_2_O on TC elimination performance in PKU‐24/PMS (inset: the corresponding first‐order rate constants). (h) Influences of nitrite with different concentrations on TC degradation performances in PKU‐24/PMS. Experimental conditions: [Catalyst] = 0.2 g L^−1^, [PMS] = 0.2 mm, [TC] = 10.0 mg L^−1^ (in (a,c,h,g)), [MeOH] = [TBA] = 40.0 mm, [L‐histidine] = 10 mm, [BQ] = [FFA] = [β‐carotene] = 5.0 mm (in (c)).

The Fenton‐like reaction activity test of PKU‐24 was conducted under dark conditions. Figure 3a shows the superior TC elimination performance of PKU‐24 with the aid of PMS, and the corresponding apparent reaction rate constant (*k*_obs_, min^−1^) reached 1.31 min^−1^ (Figure S13). The reaction filtrate could not efficiently activate PMS for TC removal, excluding the contribution from leached Co (0.24 mg L^−1^), which was below the emission standard (1.0 mg L^−1^, GB 25467–2010). Also, the poor removal efficiencies from the adsorption of PKU‐24 or individual PMS confirmed that effective TC elimination required the co‐presence of PMS and PKU‐24. Additionally, *k*_obs_ of PKU‐24/PMS was higher than that of other MOFs/PMS systems, such as MIL‐88A, MIL‐100(Fe), and BUC‐92 (Figure S14). The *k*‐value (min^−1^ M^−1^) exhibited by PKU‐24/PMS exceeded that of many reported catalytic systems (Figure 3b; Table S4), indicating outstanding Fenton‐like activity.

ROS quenching experiments (Figure 3c; Figure S15) combined with EPR measurement excluded the contributions of sulfate (SO_4_^•−^) and hydroxyl (^•^OH) radicals owing to the negligible inhibition on TC removal with the addition of methanol (MeOH) or tert‐butyl alcohol (TBA) [22, 23]. Additionally, no 5,5‐dimethyl‐1‐pyrroline N‐oxide (DMPO)‐SO_4_^•−^ or DMPO‐^•^OH signals were observed (Figure S16a), further supporting the quenching outcomes. Benzoquinone (BQ) did not lead to distinct inhibition of TC removal, and the superoxide radical (O_2_^•−^) was excluded by quantitative experiment (Figure S17) and EPR (Figure S16b) [24]. The low conversion efficiency from methyl phenyl sulfoxide (PMSO) to the featured product of methyl phenyl sulfone (PMSO_2_) suggested that high‐value Co(IV) was not the primary ROS (Figure S18) [25]. To probe the nonradical electron transfer process (ETP), in situ Raman measurement (Figure 3d) was used to capture the surface‐confined PMS intermediate (HSO_5_^−^*) [26, 27]. However, no new peak could be observed when PMS and PKU‐24 were mixed into the system, besides the peaks for PMS at ∼1059 cm^−1^ and ∼881 cm^−1^, signifying that the metastable PKU‐24‐PMS* complex was not formed. Accordingly, only ∼0.1 eV increase in potential was monitored after adding PMS in the open‐circuit potential test (Figure 3e), excluding the generation of the PKU‐24‐PMS* complex.

In contrast, when using 2,2,6,6‐tetramethyl‐1‐piperidinyloxy (TEMP) as the spin trap for ^1^O_2_ in EPR analysis [28], pronounced triplet peaks for TEMPO were observed, indicating the generation of ^1^O_2_ (Figure 3f). Specific ^1^O_2_ quenching agents, including furfuryl alcohol (FFA), L‐histidine, or β‐carotene [29, 30], significantly impaired the intensity of the TEMP‐^1^O_2_ signal and inhibited the TC removal efficiencies, confirming the dominant contribution of ^1^O_2_ to TC oxidation. Furthermore, D_2_O was selected to replace H_2_O to prolong the lifetime of ^1^O_2_ [31, 32], which remarkably accelerated the degradation kinetics by 2.98‐fold, affirming the crucial role of ^1^O_2_ in TC degradation (Figure 3g). Accordingly, the intensity of the TEMP‐^1^O_2_ signal was enhanced in D_2_O compared to H_2_O (Figure 3f). As the ROS quenching (Figure 3c) and EPR tests (Figure S16b) excluded O_2_^•−^ as the precursor for ^1^O_2_ generation [33], SO_5_^•−^, generated through one‐electron oxidation of PMS, was proposed as the major intermediate (PMS → SO_5_^•−^ → ^1^O_2_). Sodium nitrite (NO_2_^−^), as the quencher of SO_5_^•−^ [34], distantly inhibited TC degradation under different concentrations (Figure 3h; Figure S19), confirming the SO_5_^•−^‐mediated ^1^O_2_ production pathway.

### 2.3 Inert Charge‐Confinement Effect Investigation and Mechanism Insight

**FIGURE 4:**
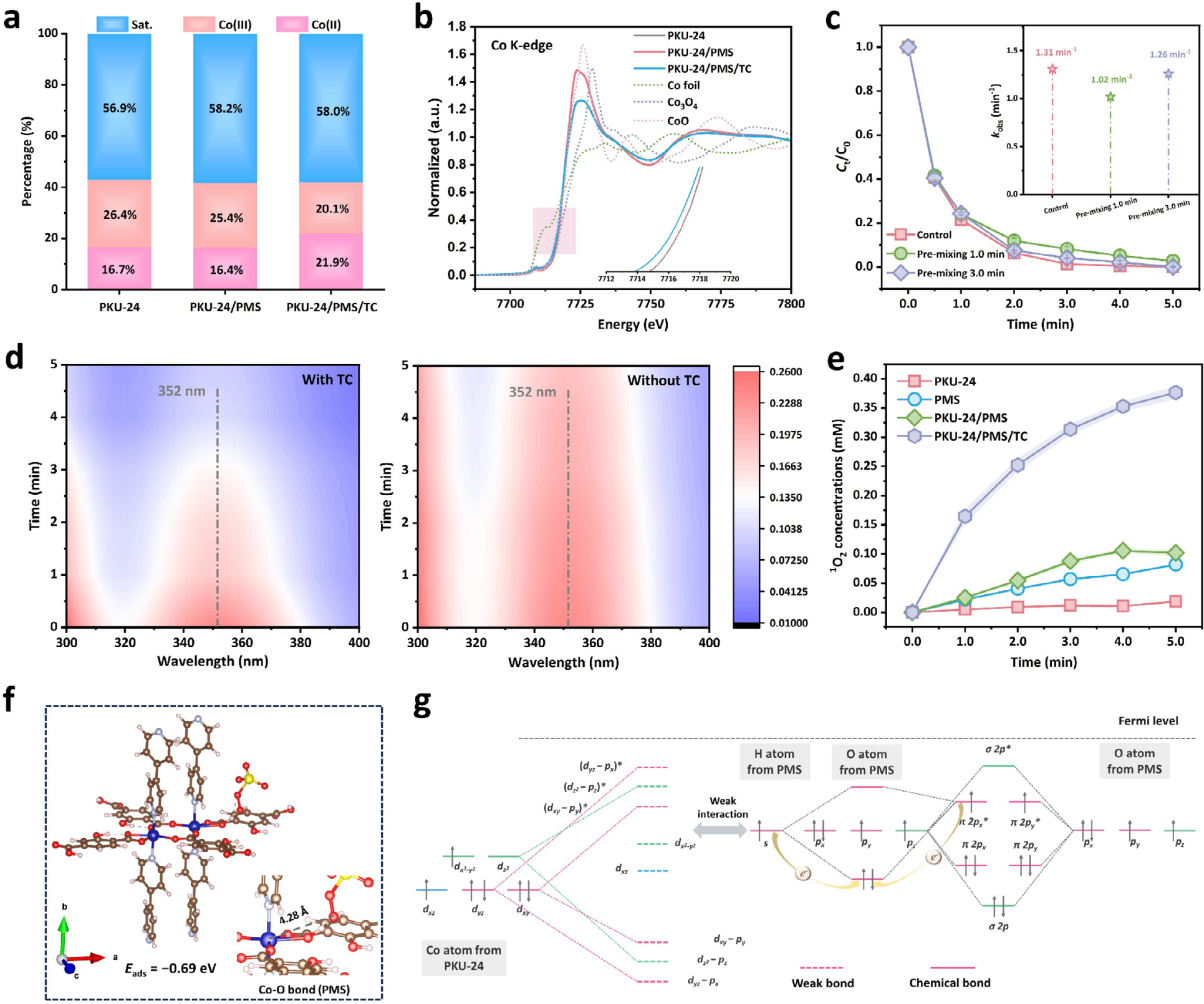
Exploration of the interaction between PMS and PKU‐24. (a) The contents of Co(II), Co(III), and Sat. in different systems obtained by XPS Co 2*p* analyses. (b) XANES spectra of Co K‐edge in different reaction systems (insert: the absorption edge of Co). (c) The influences of premixture of PMS and PKU‐24 on TC removal efficiencies (inset: the corresponding first‐order rate constants). (d) The UV absorption spectra of PMS residual concentration in different systems (352 nm was the measuring wavelength of I_3_^−^). (e) ^1^O_2_ quantitative experiments of different systems using DPBF. (f) PMS adsorption model on PKU‐24 and sorption energy calculation. (g) Schematic illustration of bonding and anti‐bonding orbitals between Co atom from PKU‐24 and O atom from PMS. Experimental conditions: [Catalyst] = 0.2 g L^−1^, [PMS] = 0.2 mm, [TC] = 10.0 mg L^−1^ (in (c,d)).

To facilitate one‐electron PMS oxidation to SO_5_^•−^, the catalytic Co sites with depressed valence state are expected. XPS Co 2*p* spectra (Figure 4a; Figure S20) confirmed the increase in the content of Co(II) from 16.7% to 21.9% after reaction, indicating that Co sites accepted electrons and resulted in the decrease in oxidation state. Meanwhile, Co K‐edge XANES profiles (Figure 4b; Figure S21) of PKU‐24 after reaction showed that the average valence state of Co turned lower. Intriguingly, the contents of Co(II)/Co(III) in PKU‐24 were almost unchanged in the presence of PMS when TC was absent. Accordingly, the valence state of Co atoms also remained unchanged, as obtained by the Co K‐edge XANES spectrum, and the invariant white line illustrated that the traditional chemical bond could not be formed between Co sites and PMS. TC seemed like a “switch” based on the above results, opening the electron transport channel between Co sites and PMS.

The premixing experiments confirmed no direct electron transfer between PKU‐24 and PMS in the absence of TC. First, changes in PMS concentration affected the TC catalytic degradation efficiency and rate (Figure S22), confirming that 0.2 mm PMS was the optimal concentration. Figure 4c shows similar TC degradation rates when the premixing time between PKU‐24 and TC was 1 or 3 min, indicating that PKU‐24 could not directly transport electrons to PMS to generate reactive species [35]. In contrast, we selected other Co‐based MOFs (Figure S23) with distinct coordination modes, including ZIF‐67 (Co‐N4) and BUC‐92 (Co‐O5), for premixing experiments. Figure S24 displays that TC degradation performances were all seriously affected in both ZIF‐67/PMS and BUC‐92/PMS systems if PMS were premixed with catalysts in advance, indicating that ZIF‐67 and BUC‐92 could all directly activate PMS. Also, DFT calculations showed that ZIF‐67 and BUC‐92 could all exhibit strong interactions and electron transfer with PMS, as evidenced by adsorption energy, projected density of states (PDOS), electron density difference, and *d*‐band center analysis, as shown in Figures S25–S29.

In addition, PMS consumption in PKU‐24/PMS and PKU‐24/PMS/TC systems was monitored by iodometry, because I^−^ could react with PMS to generate I_3_^−^, which appeared as a pale yellow color and could be detected by UV absorption spectra. As shown in Figure 4d, the slight decrease in absorbance signified the consumption of a minor quantity of PMS in the reaction system without TC, which was attributed to the activation of leaching Co ions and the adsorption of PKU‐24, because the positive zeta potential of PKU‐24 in acidic conditions could produce weak electrostatic interactions with PMS (Figure S30). The addition of TC accelerated the decay in absorbance, indicating that the existence of TC promotes PMS activation. Meanwhile, the PMS concentrations at different reaction times were also monitored (Figure S31). The existence of TC significantly accelerated PMS consumption, confirming that pollutant adsorption promotes PMS activation. Once TC was completely removed at ∼4 min, the PMS concentration remained nearly unchanged, indicating that PMS activation was largely suppressed in the absence of pollutants. 1,3‐diphenylisobenzofuran (DPBF) was used for quantitative analyses of ^1^O_2_ in different systems in Figure 4e [36, 37]. The co‐presence of TC, PMS, and PKU‐24 greatly accelerated DPBF oxidation, yielding the characteristic 1,2‐dibenzoylbenzene product via ^1^O_2_ oxidation, indicating a greater generation of ^1^O_2_ than in other control systems (PUK‐24, PMS only, and PUK‐24/PMS).

DFT calculations were employed to explain why no electron transfer process occurred between PKU‐24 and PMS. First, the PKU‐24 structural model (Figure S32) was constructed based on single‐crystal XRD and XAFS data. The saturated coordinated center [Co‐N_2_O_4_] could not form strong chemical adsorption with PMS but could instead exhibit the weak hydrogen bonding and van der Waals interactions, as indicated in Figure 4f. Consequently, PMS was captured at [Co‐N_2_O_4_] sites with a moderate negative adsorption energy (*E*_ads_ = −0.69 eV) due to the long distance (4.28 Å) between the Co site and the O atom of PMS.

We employed PDOS to analyze the electronic configurations of Co *d* orbitals from PKU‐24 and O *p* orbitals from PMS before and after adsorption (Figures S33 and S34). In an octahedral field, the Co *d* orbitals split into two e_g_ orbitals with high energy (*d*_x_2_‐y_2, *d*_z_2) and three t_2g_ orbitals with low energy (*d*_xy_, *d*_yz_, *d*_xz_), in which the *d*_xy_ and *d*_yz_ orbitals were occupied by two electrons, while *d*_xz_ and *d*_z_2 contained one spin‐up electron, respectively. As shown in Figures S35 and S36, a weak interaction induced the orbital hybridization between Co sites and hydroxyl oxygen from PMS based on the increased broadening (W_d_) to split bonding and anti‐bonding orbitals (Figure 4g; Figure S37), while failing to significantly elevate the energy gap. Moreover, the highest anti‐bonding orbitals formed by orbital hybridization remained below the Fermi level, implying no electron could be transferred between Co sites and PMS, leading to the poor activity of PKU‐24 in direct PMS activation. Meanwhile, electron density difference confirmed the absence of strong electron cloud overlap (Figure S38). Inert catalytic center induced the slight change of Bader charge, the total charge of [Co‐N_2_O_4_] center only changed from 48.74 |e| to 48.76 |e| (Figure S39).

**FIGURE 5:**
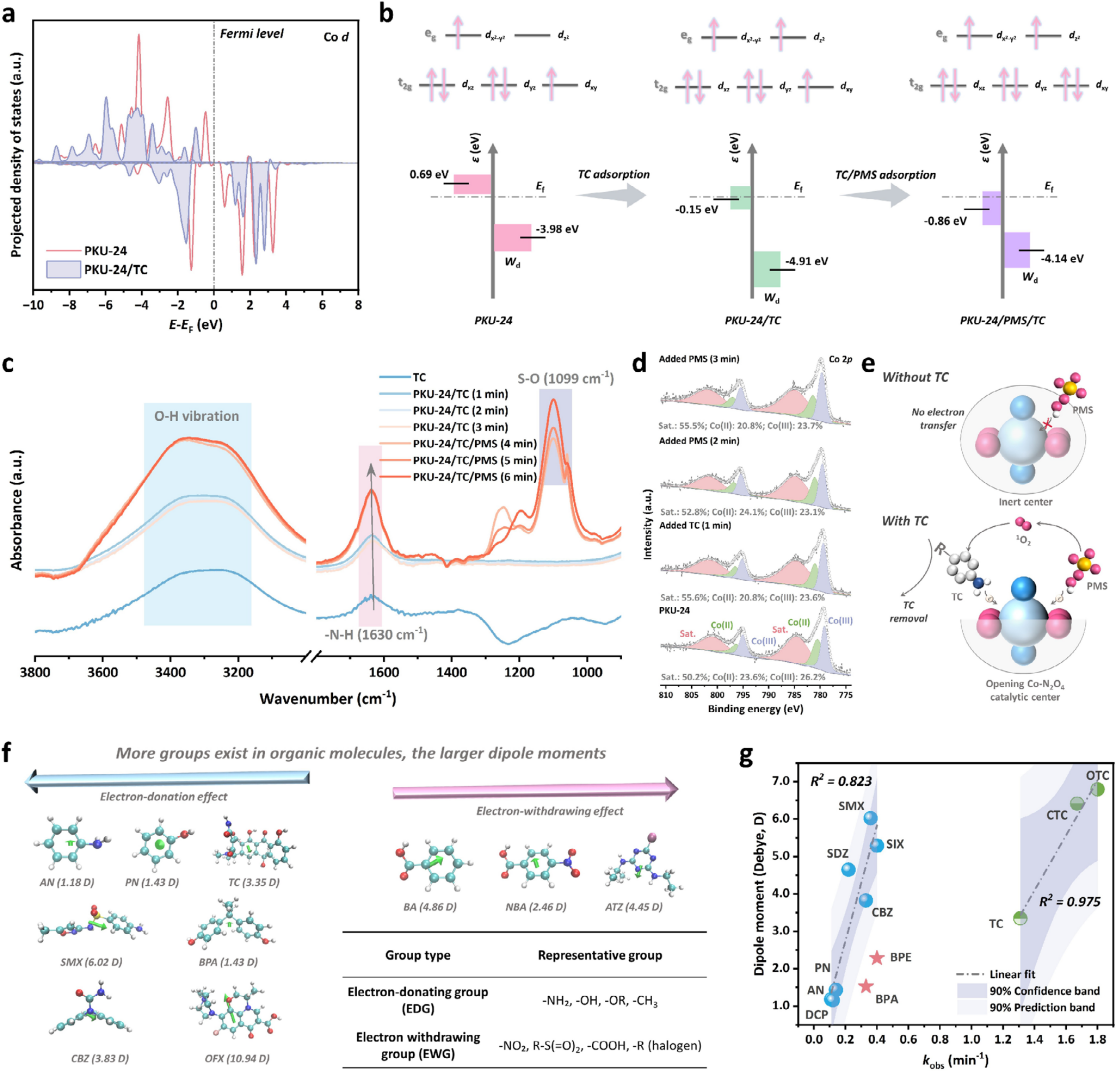
Investigation of PMS activation mechanism with the aid of pollutants. (a) PDOS of Co 3*d* orbital in PKU‐24 and PKU‐24‐TC models. (b) The change of spin‐states and *d*‐band center in different models. (c) In situ FTIR spectra of PKU‐24/PMS/TC. (d) In situ XPS Co 2*p* spectra of PKU‐24/PMS/TC. (e) Schematic illustration of PMS activation mechanism in PKU‐24/PMS system. (f) Dipole moment calculations of various organic molecules. (g) The linear correlation between organic degradation rates and their dipole moments.

However, the intervention of TC molecule as the electron donor could activate the catalytic center. The Bader charge of O and N atoms of [Co‐N_2_O_4_] center decreased about 0.06 |e| (Figure S40), impairing the charge confinement effect. While the Bader charge of Co atom increased about 0.08 |e| after TC adsorption, which was attributed to the electronic donation effect of adjacent O/N atoms and ─NH_2_ from the TC molecule. Accordingly, PDOS (Figure 5a) showed a decrease in the area of the empty orbitals, and the number of spin‐up electrons increased, signifying increased spin density (Figure 5b). High‐spin states of Co sites in PKU‐24 are preferred as electron acceptors because of the instability of single‐electron orbitals. Subsequently, co‐adsorption of TC and PMS molecules increased spin‐down electron in PDOS of Co *d* orbital and decreased spin density, indicating that the guest PMS molecule in the co‐adsorption model offered electrons to Co sites (Figure S41).

The changes in the spin states of Co sites in different models were confirmed by the effective magnetic moment (µ_eff_) from temperature‐dependent magnetic susceptibility (M–T) curves [38, 39]. Figure S42 demonstrates that the µ_eff_ values of PKU‐24, PKU‐24‐TC, and PKU‐24‐PMS‐TC models were 9.10 µ_B_, 12.11 µ_B_, and 9.69 µ_B_, respectively, which were consistent with DFT calculations that the number of spin‐up electrons increased after TC adsorption (Figure 5a). TC adsorption also redshifted the *d*‐band center of the Co site from −3.20 to −2.24 eV, allowing the anti‐bonding orbitals of PMS and PKU‐24 to cross the Fermi level, further opening the electron transfer channel between PMS and PKU‐24. The electron density difference of the PKU‐24‐PMS‐TC model showed a stronger electron cloud overlap than the PKU‐24‐PMS model (Figure S43), indicating the opening of an electron transport channel between the catalytic center and PMS upon co‐adsorption of TC. Furthermore, Gibbs free energy diagrams (Figures S44 and S45) displayed that the presence of TC molecule effectively reduced the energy barrier for the rate‐determining step (PMS* → SO_5_*) to form the critical intermediate of SO_5_^•−^ for ^1^O_2_ generation, confirming the synergy upon TC adsorption.

In situ technologies and electrochemical measurements were also conducted to elucidate the role of TC in enhanced PMS activation. In situ Fourier‐transform infrared (FTIR) spectra (Figure 5c) indicated that the ─N─H bond (∼1630 cm^−1^) [40] belonging to TC showed a red shift if PKU‐24 was added into the TC solution, confirming the strong interaction between PKU‐24 and TC. After adding the PMS solution, the stretching vibration of the ─OH group indicated the cleavage of the O‐H bond in PMS (PMS → SO_5_^•−^) [41]. In addition, the electron transfer between PKU‐24 and TC or PMS was also observed via in situ XPS. When TC was added, O─C═O, Co─O, and pyridine N all acted as electron acceptors, as reflected by their blue‐shifted binding energies (Figure S46). With the introduction of PMS, their binding energies were red shifted, implying the electron migration to Co centers. Accordingly, Co 2*p* spectra (Figure 5d) showed that the content of Co(III) decreased, while the content of Co(II) and satellite peak increased with the addition of TC, confirming the electron acceptor of Co sites with TC adsorption.

Notably, the increase in the satellite peak component from 50.2% to 55.6% implied the increase in population of unpaired electrons and total spin state [42], which was in line with the M‐T curve in Figure S42. When PMS was added, the area of Co(II) turned larger, and the satellite peak area decreased from 55.6% to 52.8%, signifying the reception of electrons from PMS and the decrease in spin state.

Amperometric *i*−*t* curves (Figure S47a) displayed a weak current response when TC was added, regardless of the presence of PMS, and a strong current response was triggered when TC and PMS simultaneously existed in the reactor. Electrochemical impedance spectroscopic (EIS) curves showed that the addition of PMS slightly increased the diameter of the arc (Figure S47b), while the co‐presence of TC and PMS greatly reduced electron transport resistance. Based on the above conclusion, PMS could not be consumed by PKU‐24 with inert charge‐confined catalytic sites. TC, as the electron‐rich pollutant, acted as a “switch” to open the electron‐transfer channel between PKU‐24 and PMS, further activating PMS via an electron‐withdrawing process, thereby promoting selective ^1^O_2_ generation (Figure 5e).

### 2.4 Probing into the Electronic Properties of Contaminants for Activating the Catalytic Center

To investigate which properties of organics could unlock the inert charge‐confined catalytic center, we selected various organic pollutants containing TC, chlorotetracycline (CTC), oxytetracycline (OTC), sulfamethoxazole (SMX), sulfisoxazole (SIX), sulfadiazine (SDZ), phenol (PN), aniline (AN), 2,4‐dichlorophenol (DCP), ciprofloxacin (CIP), ofloxacin (OFX), bisphenol A (BPA), bisphenol S (BPS), carbamazepine (CBZ), atrazine (ATZ), benzoic acid (BA) and 4‐nitrobenzoic acid (NBA) to calculate electronic properties containing the lowest unoccupied molecular orbital (LUMO), highest occupied molecular orbital (HOMO), electrostatic potential (ESP), electrophilicity index, nucleophilicity index and make the comparison (Figures S48–S50 and Table S5). Catalytic degradation experiments (Figures S51 and S52) showed that PKU‐24/PMS exhibited degradation of most electron‐rich pollutants. However, we investigated the relationship between the HOMO value, energy gap (LUMO‐HOMO), electrophilicity index, nucleophilicity index, and their degradation rates (Figure S53), which all presented poor correlation. Therefore, these factors were not decisive in determining whether targeted pollutants could activate the inert charge‐confined catalytic center of PKU‐24.

If an organic pollutant wants to activate the inert catalytic center, it must first be able to approach the catalytic sites. Consequently, adsorption experiments (Figure S51) and molecular dynamics (MD) simulations were conducted on representative organic molecules [43]. Some electron‐rich pollutants like CIP, OFX, and BPS could not be degraded, which also could not be adsorbed by PKU‐24. Figure S54 indicates that electrostatic repulsion was the main factor between CIP, OFX, or BPS and PKU‐24 under weakly acidic conditions (0.2 mm PMS dosage). In contrast, TC, OTC, CTC, SMX, SIX, and SDZ could be adsorbed through electrostatic interactions. Other organic molecules containing the benzene ring, such as BPA, AN, PN, or DCP, could be captured by PKU‐24 via π–π interactions. MD simulations revealed the interaction between pollutants and PKU‐24. As shown in Figures S55 and S56 and Video S2, MD simulations were performed to investigate the surface absorption of TC molecules to the PKU‐24 solid. The simulation modeled the free diffusion of a relatively large TC molecule along the y‐axis for 10 ns. Over this period, the TC molecule adsorbed onto the surface of the PKU‐24. The adsorption process occurred primarily through hydrogen bonding between the functional groups of TC and PKU‐24.

Additionally, Figure 5f and Figure S57 show that PKU‐24/PMS could effectively oxidize pollutants bearing electron‐rich groups, such as ─NH_2_, ─OH, or ─CH_3_, whereas pollutants that were difficult to degrade featured strong electron‐withdrawing groups, such as ─COOH or ─NO_2_. Despite CIP, OFX, and BPS being classified as electron‐rich pollutants due to their high HOMO values, electron‐withdrawing functionalities, such as halogens (‐F) or R‐S(= O)_2_, will significantly reduce the molecular electron‐donating capacity, even in the presence of electron‐rich groups such as CIP, OFX, and BPS. Collectively, the electron‐donating and ‐withdrawing groups affected the size and direction of the overall dipole moment of organic molecules, where the direction of the dipole moment was defined from the negative charge to the positive charge. When electron‐rich groups were located near the dipole moment origin, pollutants could act as molecular “switches” to control catalytic initiation and termination, revealing a well‐defined linear relationship with the degradation rate (Figure 5g). We also employed DFT calculations to explain why pollutants with small dipole moments exhibit poor degradation performances. PN can spontaneously adsorb onto PKU‐24 via π–π interactions, but it induced only a minor perturbation in the electron density of Co sites, with the Bader charge decreasing from 7.75 |e| to 7.72 |e| (Figure S58). In contrast, the adsorption of TC exerted a stronger redistribution of electron density at the Co center (from 7.75 |e| to 7.83 |e|), which facilitates reopening of the electron‐transfer pathway and accounts for the higher removal efficiency of TC compared to PN.

The exception for BPA was attributed to the dipole moment being canceled by its symmetrical structure. However, the abundant electron‐rich groups (two ─OH and two ─CH_3_) of BPA endowed it with a strong electron‐donating property, which could unlock the inert catalytic site of PKU‐24 for PMS activation, resulting in ∼36% degradation of BPA. Bisphenol E (BPE) could partially unlock the inert catalytic site of PKU‐24, achieving ∼39% degradation efficiency (Figure S59). Although BPE contains multiple electron‐rich functional groups, its symmetric molecular structure results in a relatively small dipole moment (2.28 D). This observation suggests that while dipole moment plays a key role in pollutant‐induced activation, electron‐rich functional groups may also contribute to the “switching” behavior.

Based on the above conclusion, the decisive factors in activating inert catalytic centers were the size and direction of the dipole moment, rather than the HOMO value, energy gap, or electrophilicity index. Additionally, premixing experiments were conducted using BPA, SMX, OTC, and SIX to examine whether the same experimental phenomenon could be observed for other pollutants. As shown in Figure S60, the premixture of PKU‐24 and PMS did not affect the degradation rates or catalytic rates, confirming that PKU‐24 remains inert toward PMS activation in the absence of pollutants. Furthermore, in situ FTIR spectra (Figure S61) showed a red shift of the ─N─H bond from SMX, while the ─OH from BPA exhibited a stretching vibration upon addition of PKU‐24 (Figure S62), which all confirmed the interaction between the catalyst and the electron‐rich groups.

### 2.5 Practical Environmental Application Investigation

The impacts of co‐existing factors (inorganic anions and humic acid) and solution pH were also assessed (Figures S63 and S64). The PKU‐24/PMS exhibited strong immunity to interferences and maintained TC or SIX removal efficiencies in the presence of background factors and across a wide operational pH window from 3.0 to 10.0. When treating TC solutions with different initial concentrations (5–30 mg L^−1^), the PKU‐24/PMS system still maintained high degradation efficiencies (>90%), although the reaction rate decreased at higher concentrations (Figure S65), demonstrating its broad applicability for wastewater treatment. Combining the Fukui index of the TC molecule (Table S6) and non‐targeted screening results for UPLC‐MS product identification (Figures S66 and S67), possible TC elimination pathways were proposed (Figure S68). The corresponding toxicities of pristine TC and its possible intermediates showed that most intermediates exhibited depressed biotoxicity (Figure S69). The growth condition of *Escherichia coli* (*E. coli*) via adding different solutions containing deionized water, TC solution (the diameters of inhibition zones were 12.2 mm), and TC degradation solution confirmed the low toxicity of intermediates (Figure S70). In addition, the TC‐degraded solution was used to cultivate bean sprouts. The germination rate and growth status of bean sprouts were comparable to the bean sprouts cultivated with tap water, confirming the detoxification ability of the PKU‐24/PMS system (Figure S71). Cyclic experiments illustrated the good reusability and water stability of PKU‐24 (Figures S72 and S73).

**FIGURE 6:**
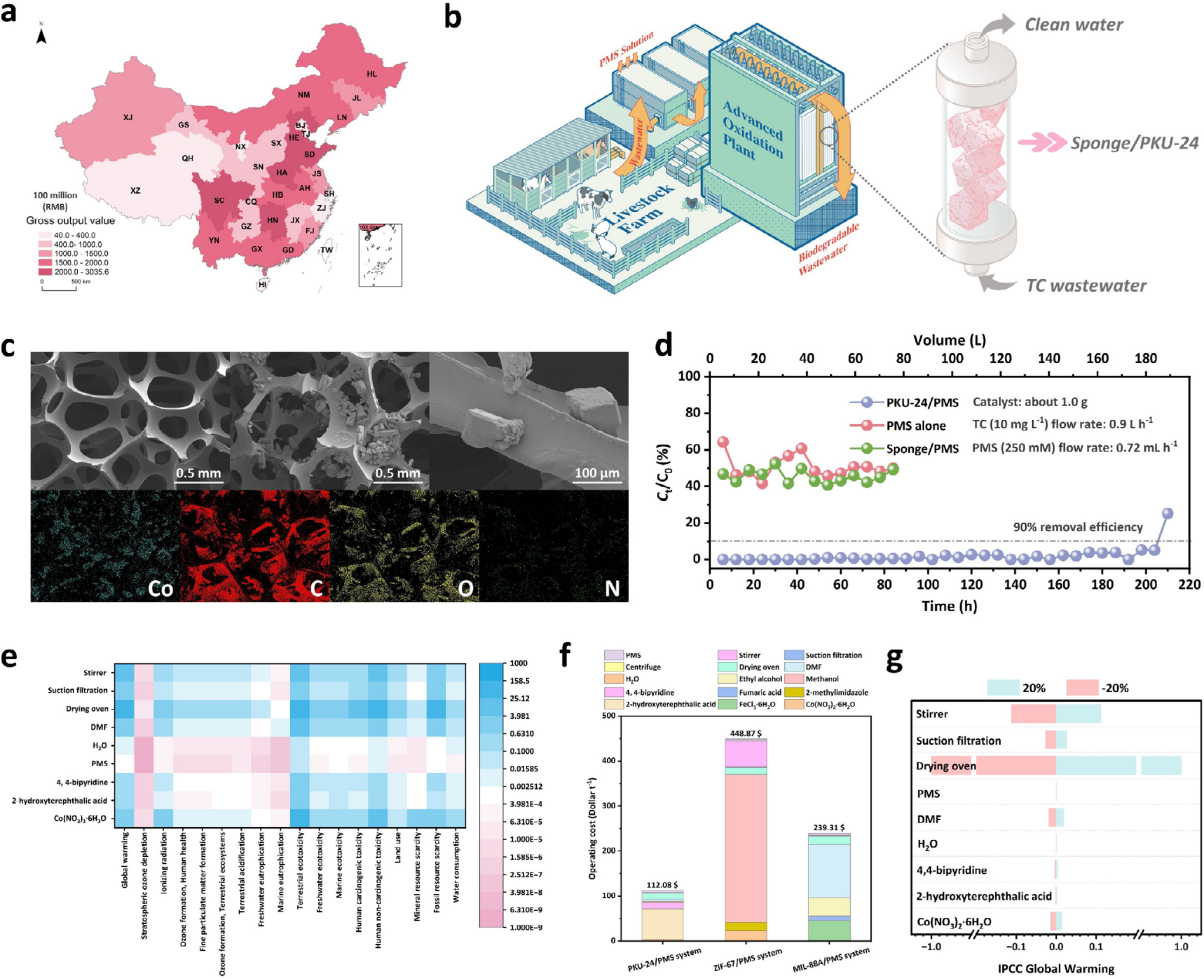
Environmental applications of PKU‐24/PMS. (a) Annual output value of animal husbandry in each region of China (www.stats.gov.cn/sj/). (b) Schematic illustration of the long‐time treatment reactor and potential application scenario. (c) SEM images and elemental mapping of PKU‐24/sponge. (d) TC removal efficiencies of different systems in the long‐time treatment reactor. (e) Impact assessment for purifying 1.0‐ton TC wastewater by PKU‐24/PMS. (f) Comparison of the operating costs of different systems. (g) Sensitivity analysis of PKU‐24/PMS system.

Antibiotics such as tetracyclines (TCs) are widely used in livestock farming, and China alone reports annual animal husbandry output values exceeding 2.1 billion USD (Figure 6a), underscoring both the scale of usage and the urgent need for effective wastewater treatment solutions (Figure 6b). To demonstrate practical feasibility, we conducted catalytic tests on the PKU‐24 synthesized at the kilogram scale (the cost of synthesizing each gram of PKU‐24 was only ∼0.52 USD), finding that TC could still be rapidly eliminated with the assistance of PMS (Figure S74). Furthermore, we immobilized PKU‐24 on a polymeric sponge using polyvinyl butyral (PVB) as the adhesive [44], constructing a continuous‐flow treatment device (Figure 6c). With only ∼1 g of PKU‐24, the system maintained >90% TC removal over 200 h of operation (Figure 6d), while PXRD and SEM confirmed the chemical stability of the catalyst (Figure S75). Life‐cycle assessment revealed that PKU‐24/PMS exhibited clear environmental advantages: unlike MOFs such as MIL‐88A (DMF) and ZIF‐67 (methanol), the synthesis of PKU‐24 was primarily performed in water, where only 1 mL of DMF was used to promote the coordination reaction, thereby avoiding extensive use of organic solvents. Moreover, lower PMS dosages were required to achieve comparable treatment performance. As a result, PKU‐24/PMS significantly reduced emissions of CO_2_, PM_2.5_, SO_2_, and NO_x_ (Figure 6e; Figure S76 and Tables S7–S10). Economic analysis further confirmed its competitiveness, with treatment costs for 1 t of TC‐containing wastewater estimated at ∼112 USD in batch mode, less than half those of MIL‐88A/PMS (239 USD) and ZIF‐67/PMS (449 USD), and decreasing to ∼13 USD when operated continuously (Figure 6f). Although the synthesis of PKU‐24 requires relatively expensive organic ligands, both H_2_BDC‐OH (∼10 USD kg^−1^) and 4,4‐bpy (∼2.18 USD kg^−1^) were commercially available ligands with mature industrial production routes. For large‐scale synthesis, the use of industrial‐grade raw materials and the optimization of ligand stoichiometry can further reduce material costs. In addition, the cost impact could be mitigated through partial mother liquor recycling, a well‐established, immediately implementable strategy for MOF‐scale‐up production. Previous studies [45, 46] have shown that recovered mother liquor after filtration could be reused for 3–5 subsequent batches with only minor supplementation of fresh ligands and metal salts, without compromising product crystallinity or performance. These approaches can effectively reduce the cost contribution of organic ligands during scale‐up production. Sensitivity analysis (Figure 6g; Figure S77) highlighted the energy consumption as the dominant factor influencing CO_2_ emissions, emphasizing that optimizing both synthesis and operational conditions would be essential to maximize the environmental and economic benefits of PKU‐24/PMS in future large‐scale application.

## 3 Conclusions

In this work, we synthesize a new Co‐MOF, PKU‐24, to drive Fenton‐like reactions for wastewater purification. In the PKU‐24/PMS system, the charge‐confined catalytic center (Co‐N_2_O_4_) loses its direct electron‐donating ability in pollutant‐free environments, preventing PMS activation and consumption. Remarkably, electron‐rich microcontaminants act as molecular “switches,” reopening the electron‐transfer channel between Co sites and PMS, thereby triggering ^1^O_2_ generation and achieving nearly 100% utilization of PMS. In situ FTIR and XPS measurements reveal strong interactions between the functional groups of pollutants, such as ─OH and ─NH_2_, and the Co centers, which increase the spin state and unlock otherwise inert sites, in agreement with DFT calculations. Furthermore, we identify dipole moment as a decisive descriptor that governs whether organics can activate the catalytic center.

From an application perspective, the ^1^O_2_ pathway demonstrates strong resistance to interference from co‐existing constituents and an excellent detoxification effect during real wastewater treatment. Additionally, the developed synthesis methods for PKU‐24 in kilograms help the PKU‐24/PMS system demonstrate excellent practical application prospects, with large‐scale synthesis yielding ∼0.55 kg per batch and a cost of only ∼0.52 USD per gram. The PKU‐24/sponge continuous reactor sustains effective performance for over 200 h, highlighting its stability and scalability. Overall, this study provides new mechanistic insights into Fenton‐like catalysis, revealing that contaminants themselves can dictate ROS generation. This switchable mechanism not only achieves near‐complete oxidant utilization but also establishes a theoretical foundation for designing smart charge‐confined MOFs for sustainable water purification.

## 4 Materials and Methods

### 4.1 Chemicals

All the chemical reagents for catalyst syntheses and experimental evaluation in this work are listed in Supporting Information (Section S1).

### 4.2 Fabrication of PKU‐24

The new crystal MOF (named PKU‐24) was synthesized by a simple hydrothermal method. In detail, 0.3 mmol of Co(NO_3_)_2_·6H_2_O, 0.3 mmol of 4,4‐bpy, and 0.3 mmol of H_2_BDC‐OH were mixed in a 25 mL Teflon‐lined autoclave containing a H_2_O (10 mL)/DMF (1 mL) mixed solution, and the reactor was heated to 110°C for 12 h. Finally, the prepared pink crystal catalyst was washed with DMF and deionized water three times, and dried at 60°C. The fabrication methods of other MOFs were listed in Section S2. The detailed characterizations of the catalysts were presented in Sections S3–S6.

In addition, we developed other high‐yield, scalable methods for producing PKU‐24 (Figure 1). As for the hydrothermal method, a 25 mL volume in a Teflon‐lined autoclave (<20 mg) could be expanded to 2000 mL, yielding a single synthesis of ∼57 g. Accordingly, the dosage of both Co(NO_3_)_2_·6H_2_O and the two organic linkers needed to be increased to 150 mmol, and the reactor was further heated to 110°C for 12 h. Additionally, the hydrothermal reaction could also be performed in a high‐borosilicate glass container. The volume of the container could be expanded from 50 mL to 10 000 mL. In a 10 000 mL glass bottle, the dosage of Co(NO_3_)_2_·6H_2_O and the two organic ligands could be increased to 1500 mmol, but the heating time would need to be extended to 24 h. After washing and drying, the yield of PKU‐24 produced in a single synthesis was ∼0.55 kg.

### 4.3 Batch Catalytic Experiments

The Fenton‐like reactions were all conducted in the dark. Specifically, 10 mg of the crystal PKU‐24 catalyst was added to a 50 mL solution containing 10 mg L^−1^ of organic pollutants, stirring the solution at 300 rpm min^−1^ under room temperature (25 ± 2°C). The required pH solution was adjusted with HNO_3_ or NaOH aqueous solutions of suitable concentration. The reaction began with the addition of 0.2 mm PMS. About 1 mL of the reaction solution was filtered using a PTFE membrane every 1 min for the determination of the residual concentration. The addition of 10 µL methanol and 10 µL of sodium thiosulfate solution in the filtered solution could ensure the termination of the reaction. The residual concentrations of various organics were determined by ultra‐high performance liquid chromatography (UHPLC; Thermo Scientific Vanquish Flex), and the detailed data analysis and test methods were listed in Section S7 and Table S1.

### 4.4 Theoretical Calculations

All of the first‐principles calculations in this work were performed using Vienna Ab Initio Simulation Package (VASP) [47]. The exchange‐correlation energy was described by the Perdew–Burke–Ernzerhof (PBE) form of generalized‐gradient approximation (GGA) exchange‐correlation energy functional [48]. The spin state and DFT + U (*d* = 3.4) were set to perform structure optimization [49]. Energy cutoff was 350.0 eV, *k*‐point spacing was smaller than 0.05 Å^−1^. The convergence standards for optimizing bulk and surface were set to be less than 2.0 × 10^−5^ eV (EDIFF) and 0.05 eV/Å (EDIFFG). The specific calculation items are presented in Section S8.

All calculations for organic contaminants were conducted using the Gaussian C program [50]. The geometric structure optimization and property calculations for all organics were completed using the DFT method at the B3LYP/6‐311+G** level [51]. MD simulations details are provided in Section S9. The life cycle perspective calculations are provided in Section S10.

## Funding

Financial supports from the National Natural Science Foundation of China (22576005), Shenzhen Science and Technology Program (JCYJ20241202125900002), and the 2023 Shenzhen Sustainable Supporting Funds for Colleges and Universities (20231121170027002) were greatly acknowledged.

## Conflicts of Interest

The authors declare no conflicts of interest.

## Supporting information




**Supporting File 1**: adma73103‐sup‐0001‐SuppMat.docx.

**Supporting File 2**: adma73103‐sup‐0002‐VideoS1.mp4.

**Supporting File 3**: adma73103‐sup‐0003‐VideoS2.mp4.

## Data Availability

The data that support the findings of this study are available from the corresponding author upon reasonable request.

## References

[1] Z.‐Y. Guo , Y. Si , W.‐Q. Xia , et al., “Electron Delocalization Triggers Nonradical Fenton‐Like Catalysis over Spinel Oxides,” Proceedings of the National Academy of Sciences 119 (2022): 2201607119, 10.1073/pnas.2201607119. PMC9351537 35878043

[2] L. Chen , J. Hu , A. G. Borthwick , et al., “Solar‐light‐activated Periodate for Degradation and Detoxification of Highly Toxic 6PPD‐quinone at Environmental Levels,” Nature Water 2 (2024): 453–463, 10.1038/s44221-024-00236-3.

[3] Y.‐J. Zhang , J.‐S. Tao , Y. Hu , et al., “Metal Oxyhalide‐Based Heterogeneous Catalytic Water Purification with Ultralow H_2_O_2_ Consumption O_2_ Consumption,” Nature Water (2024): 770–781, 10.1038/s44221-024-00281-y.

[4] D. Zhang , Y. Li , P. Wang , J. Qu , Y. Li , and S. Zhan , “Dynamic Active‐site Induced by Host‐guest Interactions Boost the Fenton‐Like Reaction for Organic Wastewater Treatment,” Nature Communications 14 (2023): 3538, 10.1038/s41467-023-39228-4. PMC10272134 37322015

[5] Y. Zhou , W. Guo , Y. Li , et al., “Insights into Free Radical and Non‐radical Routes Regulation for Water Cleanup,” Nature Communications 16 (2025): 7753, 10.1038/s41467-025-63235-2. PMC12368032 40835831

[6] Q. Tian , X. Zhang , J. Chang , et al., “Silico‐oxygen Bonding Integrated with Nano‐Size Pore Enrichment Enables Sustainable Low‐oxidant‐consumption Fenton‐Like Chemistry,” Water Research 281 (2025): 123550, 10.1016/j.watres.2025.123550. 40174569

[7] Y. Gao , T. Wu , C. Yang , et al., “Activity Trends and Mechanisms in Peroxymonosulfate‐Assisted Catalytic Production of Singlet Oxygen over Atomic Metal‐N‐C Catalysts,” Angewandte Chemie International Edition 60 (2021): 22513–22521, 10.1002/anie.202109530. 34387407

[8] X. Tian , T. Luo , Y. Nie , et al., “New Insight into a Fenton‐Like Reaction Mechanism over Sulfidated β‐FeOOH: Key Role of Sulfidation in Efficient Iron (III) Reduction and Sulfate Radical Generation,” Environmental Science & Technology 56 (2022): 5542–5551, 10.1021/acs.est.2c00132. 35412804

[9] S. Li , Y. Yang , H. Zheng , et al., “Introduction of Oxygen Vacancy to Manganese Ferrite by Co Substitution for Enhanced Peracetic Acid Activation and 1O2 Dominated Tetracycline Hydrochloride Degradation under Microwave Irradiation,” Water Research 225 (2022): 119176, 10.1016/j.watres.2022.119176. 36191527

[10] J. Jiang , S. Liu , D. Shi , et al., “Spin state‐dependent in‐situ Photo‐Fenton‐Like Transformation from Oxygen Molecule towards Singlet Oxygen for Selective Water Decontamination,” Water Research 244 (2023): 120502, 10.1016/j.watres.2023.120502. 37651870

[11] Y. Tao , Y. Hou , H. Yang , et al., “Interlayer Synergistic Reaction of Radical Precursors for Ultraefficient ^1^O_2_ Generation via Quinone‐based Covalent Organic Framework,” Proceedings of the National Academy of Sciences 121 (2024): 2401175121, 10.1073/pnas.2401175121. PMC11420197 39250664

[12] T. Yang , M. Chen , J. Li , et al., “One Heterogeneous Catalyst Drives Two Selective Fenton‐Like Reaction Modes for Sustainable Water Decontamination,” Environmental Science & Technology 59 (2025): 8155–8166, 10.1021/acs.est.4c13436. 40239063

[13] X. Ma , H. Liu , W. Yang , G. Mao , L. Zheng , and H.‐L. Jiang , “Modulating Coordination Environment of Single‐atom Catalysts and Their Proximity to Photosensitive Units for Boosting MOF Photocatalysis,” Journal of the American Chemical Society 143 (2021): 12220–12229, 10.1021/jacs.1c05032. 34324821

[14] X. Ma , Y. Chai , P. Li , and B. Wang , “Metal–Organic Framework Films and Their Potential Applications in Environmental Pollution Control,” Accounts of Chemical Research 52 (2019): 1461–1470, 10.1021/acs.accounts.9b00113. 31074608

[15] Z.‐B. Wang , H.‐Y. Chu , M. Chang , and C.‐C. Wang , “Tailoring Metal–organic Frameworks: from Morphological Control to Superstructure Assembly,” Coordination Chemistry Reviews 554 (2026): 217606, 10.1016/j.ccr.2026.217606.

[16] H.‐C. Zhou , J. R. Long , and O. M. Yaghi , “Introduction to Metal–Organic Frameworks,” Chemical Reviews 112 (2012): 673–674, 10.1021/cr300014x. 22280456

[17] A. Kirchon , J. Li , F. Xia , et al., “Modulation versus Templating: Fine‐Tuning of Hierarchally Porous PCN‐250 Using Fatty Acids To Engineer Guest Adsorption,” Angewandte Chemie International Edition 58 (2019): 12425–12430, 10.1002/anie.201905006. 31265165

[18] J. Krzystek , A. Ozarowski , and J. Telser , “Multi‐frequency, High‐field EPR as a Powerful Tool to Accurately Determine Zero‐field Splitting in High‐spin Transition Metal Coordination Complexes,” Coordination Chemistry Reviews 250 (2006): 2308–2324, 10.1016/j.ccr.2006.03.016.

[19] S. J. Tereniak , R. K. Carlson , L. J. Clouston , et al., “Role of the Metal in the Bonding and Properties of Bimetallic Complexes Involving Manganese, Iron, and Cobalt,” Journal of the American Chemical Society 136 (2014): 1842–1855, 10.1021/ja409016w. 24125042

[20] P. Zhang , Y. Yang , X. Duan , Y. Liu , and S. Wang , “Density Functional Theory Calculations for Insight into the Heterocatalyst Reactivity and Mechanism in Persulfate‐based Advanced Oxidation Reactions,” ACS Catalysis 11 (2021): 11129–11159, 10.1021/acscatal.1c03099.

[21] Q. Tian , X. Xu , and X. Duan , “Application‐oriented Advanced Oxidation Processes: Research Priorities for Upscaling and Deployment,” Environmental Science & Technology 59 (2025): 16823–16826, 10.1021/acs.est.5c09384. 40772697

[22] Z.‐Y. Guo , C. X. Li , M. Gao , et al., “Mn−O Covalency Governs the Intrinsic Activity of Co‐Mn Spinel Oxides for Boosted Peroxymonosulfate Activation,” Angewandte Chemie International Edition 60 (2021): 274–280, 10.1002/anie.202010828. 32965786

[23] F. Liu , P. Zhou , Y. Hou , et al., “Covalent Organic Frameworks for Direct Photosynthesis of Hydrogen Peroxide from Water, Air and Sunlight,” Nature Communications 14 (2023): 4344, 10.1038/s41467-023-40007-4. PMC10356944 37468482

[24] Z. Wei , S. Zhao , W. Li , et al., “Artificial Photosynthesis of H_2_O_2_ through Reversible Photoredox Transformation between Catechol and o‐benzoquinone on Polydopamine‐coated CdS,” ACS Catalysis 12 (2022): 11436–11443, 10.1021/acscatal.2c03288.

[25] Y. Lin , Y. Wang , Z. Weng , et al., “Coordination Engineering of Heterogeneous High‐valent Fe(IV)‐oxo for Safe Removal of Pollutants via Powerful Fenton‐Like Reactions,” Nature Communications 15 (2024): 10032, 10.1038/s41467-024-54225-x. PMC11576887 39562564

[26] Z.‐S. Zhu , Y. Wang , P. Wang , et al., “Multidimensional Engineering of Single‐atom Cobalt Catalysts for Ultrafast Fenton‐Like Reactions,” Nature Water 3 (2025): 211–221, 10.1038/s44221-024-00382-8.

[27] J. Mao , K. Yin , Y. Zhang , et al., “Ultrafast Oxidation of Refractory Organics via PMS Activation by Si‐O Doped Biomimetic Montmorillonite: Simultaneous Enhanced Radical/Electron Transfer Pathways and Efficient Catalytic Membrane System,” Applied Catalysis B: Environmental 342 (2024): 123428, 10.1016/j.apcatb.2023.123428.

[28] L. Chen , J. Duan , P. Du , W. Sun , B. Lai , and W. Liu , “Accurate Identification of Radicals by in‐situ Electron Paramagnetic Resonance in Ultraviolet‐based Homogenous Advanced Oxidation Processes,” Water Research 221 (2022): 118747, 10.1016/j.watres.2022.118747. 35728498

[29] Z. Yang , J. Qian , A. Yu , and B. Pan , “Singlet Oxygen Mediated Iron‐based Fenton‐Like Catalysis under Nanoconfinement,” Proceedings of the National Academy of Sciences 116 (2019): 6659–6664, 10.1073/pnas.1819382116. PMC6452667 30872470

[30] A. D. Bokare and W. Choi , “Singlet‐oxygen Generation in Alkaline Periodate Solution,” Environmental Science & Technology 49 (2015): 14392–14400, 10.1021/acs.est.5b04119. 26594871

[31] Y.‐H. Li , C.‐C. Wang , F. Wang , et al., “Nearly Zero Peroxydisulfate Consumption for Persistent Aqueous Organic Pollutants Degradation via Nonradical Processes Supported by In‐Situ Sulfate Radical Regeneration in Defective MIL‐88B (Fe),” Applied Catalysis B: Environmental 331 (2023): 122699, 10.1016/j.apcatb.2023.122699.

[32] J. Jiang , S. Liu , B. Zhao , et al., “Angstrom Confinement‐Triggered Adaptive Spin State Transition of CoMn Dual Single Atoms for Efficient Singlet Oxygen Generation,” Advanced Materials 37 (2025): 2417834, 10.1002/adma.202417834. 39901371

[33] Y. Bu , H. Li , W. Yu , et al., “Peroxydisulfate Activation and Singlet Oxygen Generation by Oxygen Vacancy for Degradation of Contaminants,” Environmental Science & Technology 55 (2021): 2110–2120, 10.1021/acs.est.0c07274. 33427455

[34] J. Zhen , J. Sun , X. Xu , et al., “M−N 3 Configuration on Boron Nitride Boosts Singlet Oxygen Generation via Peroxymonosulfate Activation for Selective Oxidation,” Angewandte Chemie International Edition 63 (2024): 202402669, 10.1002/anie.202402669. 38637296

[35] Y.‐H. Li , T. Li , X. Hu , et al., “Synchronous Increase in Spin‐State Induced by Advanced 3*d*–4*d* Orbital Hybridization for Enhancing Radical Yield,” Advanced Functional Materials 35 (2025): 14549, 10.1002/adfm.202514549.

[36] Z. Chen , J. Wang , B. Yang , et al., “Organic Carbon Transfer Process in Advanced Oxidation Systems for Water Clean‐up,” Nature Water 3 (2025): 334–344, 10.1038/s44221-025-00399-7.

[37] J. Jiang , X. Wang , Y. Liu , et al., “Photo‐Fenton Degradation of Emerging Pollutants over Fe‐POM Nanoparticle/Porous and Ultrathin G‐C_3_N_4_ Nanosheet with Rich Nitrogen Defect: Degradation Mechanism, Pathways, and Products Toxicity Assessment,” Applied Catalysis B: Environmental 278 (2020): 119349, 10.1016/j.apcatb.2020.119349.

[38] J. Yuan , F. Chen , W. Feng , et al., “Dynamic Switching Spin State of Fe Single Atoms for Piezoelectric‐Mediated Overall Nitrogen Fixation Photosynthesis,” Advanced Materials 37 (2025): 2504015, 10.1002/adma.202504015. 40401403

[39] H. Zhang , H. C. Chen , S. Feizpoor , et al., “Tailoring Oxygen Reduction Reaction Kinetics of Fe−N−C Catalyst via Spin Manipulation for Efficient Zinc–Air Batteries,” Advanced Materials 36 (2024): 2400523, 10.1002/adma.202400523. 38594481

[40] S. Xie , J. Fu , Q. Huang , et al., “Electronic Modulation and Active Site Exposure Using C_60_ Fullerenolamine Enable High‐Performance Alcohol Oxidation on Pd Metallene Catalysts,” Angewandte Chemie 137 (2025): 202506044, 10.1002/ange.202506044. 40468840

[41] M.‐Y. Lan , Y.‐H. Li , C.‐C. Wang , et al., “Multi‐channel Electron Transfer Induced by Polyvanadate in Metal‐organic Framework for Boosted Peroxymonosulfate Activation,” Nature Communications 15 (2024): 7208, 10.1038/s41467-024-51525-0. PMC11341957 39174565

[42] C. Huck‐Iriart , L. Soler , A. Casanovas , et al., “Unraveling the Chemical state of Cobalt in Co‐based Catalysts during Ethanol Steam Reforming: an in Situ Study by near Ambient Pressure XPS and XANES,” ACS Catalysis 8 (2018): 9625–9636, 10.1021/acscatal.8b02666.

[43] R. Zhao , Q. Wang , Y. Yao , et al., “Pd Single Atoms Guided Proton Transfer along an Interfacial Hydrogen Bond Network for Efficient Electrochemical Hydrogenation,” Science Advances 11 (2025): adu1602, 10.1126/sciadv.adu1602. PMC12333693 40779631

[44] X.‐H. Yi , Y. Gao , C.‐C. Wang , Y.‐H. Li , H.‐Y. Chu , and P. Wang , “Photocatalytic Cr(VI) Reduction over MIL‐88A(Fe) on Polyurethane Sponge: from Batch to Continuous‐flow Operation,” Chinese Chemical Letters 34 (2023): 108029, 10.1016/j.cclet.2022.108029.

[45] Y. Feng , Z. Kang , Z. Wang , et al., “Preprocessed Monomer Interfacial Polymerization for Scalable Fabrication of High‐Valent Cluster‐Based Metal–Organic Framework Membranes,” Journal of the American Chemical Society 146 (2024): 33452–33460, 10.1021/jacs.4c10241. 39540404

[46] K. Wu , W.‐T. Zeng , J.‐Y. Huang , et al., “Covalent‐Coordination‐Dual‐Driven in Situ Modular Assembly of 12‐Connected Nickel‐Based Metal–Organic Frameworks,” Journal of the American Chemical Society 148 (2026): 9920–9929, 10.1021/jacs.5c22441. 41742633

[47] J. Hafner , “Ab‐Initio Simulations of Materials Using VASP: Density‐Functional Theory and beyond,” Journal of Computational Chemistry 29 (2008): 2044–2078, 10.1002/jcc.21057. 18623101

[48] G. Kresse and D. Joubert , “From Ultrasoft Pseudopotentials to the Projector Augmented‐wave Method,” Physical Review B 59 (1999): 1758–1775, 10.1103/PhysRevB.59.1758.

[49] A. Jain , G. Hautier , S. P. Ong , et al., “Formation Enthalpies by Mixing GGA and GGA+ U Calculations,” Physical Review B 84 (2011): 045115, 10.1103/PhysRevB.84.045115.

[50] C. Weedbrook , S. Pirandola , R. García‐Patrón , et al., “Gaussian Quantum Information,” Reviews of Modern Physics 84 (2012): 621–669, 10.1103/RevModPhys.84.621.

[51] F. Momany , M. Appell , G. Strati , and J. Willett , “B3LYP/6‐311++ G** Study of Monohydrates of α‐and β‐d‐glucopyranose: Hydrogen Bonding, Stress Energies, and Effect of Hydration on Internal Coordinates,” Carbohydrate Research 339 (2004): 553–567, 10.1016/j.carres.2003.10.013. 15013392

