# Supplementary material for: Inert Catalytic Sites Unlocked by Micropollutants for Rapid Water Decontamination with Near‐Complete Chemical Utilization
Source: Adv Mater. 2026 Apr 19;38(28):e73103. doi: 10.1002/adma.73103 (PMC13181517; doi:10.1002/adma.73103)
Supplement: Supplementary file 1 — Supporting File 1: adma73103‐sup‐0001‐SuppMat.docx. [file ADMA-38-e73103-s002.docx]

Supporting Information

Inert Catalytic Sites Unlocked by Micropollutants for Rapid Water Decontamination with Near-Complete Chemical Utilization

Yu-Hang Li^1^, Mingyi Liu^1^, Yuanhao Li^2^, Yaran Bai^1^, Fu-Xue Wang^3^, Xiaoyi Hu^1^, Xiaoguang Duan^4,^ *, Haodong Ji^1,^ *

**Section S1: Chemicals**

Iron chloride hexahydrate (FeCl_3_·6H_2_O), 4,4-bipyridine (4,4-bpy), 2-hydroxyterephthalic acid (H_2_BDC-OH), 1,3,5-benzenetricarboxylic acid, fumaric acid (FA), peroxymonosulfate (PMS, KHSO_5_, 4.5% active oxygen), deuterium oxide (D_2_O), tert butyl alcohol (TBA), phenol (PN), aniline (AN), 2,4-dichlorophenol (DCP) and 1,3-Diphenylisobenzofuran (DPBF) were brought from J&K company (China). Cobalt nitrate hexahydrate (Co(NO_3_)_2_·6H_2_O), cobalt chloride hexahydrate (CoCl_2_·6H_2_O), iron powder (Fe^0^), 2-methylimidazole (c), tetracycline (TC), chlorotetracycline (CTC), oxytetracycline (OTC), sulfamethoxazole (SMX), sulfadiazine (SDZ), sulfisoxazole (SIX), ciprofloxacin (CIP), ofloxacin (OFX), bisphenol A (BPA), bisphenol S (BPS), bisphenol E (BPE), carbamazepine (CBZ), atrazine (ATZ), benzoic acid (BA), 4-nitrobenzoic acid (NBA), methyl phenyl sulfoxide (PMSO), methyl phenyl sulfone (PMSO_2_), furfuryl alcohol (FFA), humic acid (HA), benzoquinone (BQ), nitrotetrazolium blue chloride (NBT), L-histidine, β-carotene, potassium iodide (KI), potassium sulfate (K_2_SO_4_), sodium nitrite (NaNO_2_), monosodium phosphate (NaH_2_PO_4_), sodium chloride (NaCl), sodium hydroxide (NaOH), sodium bicarbonate (NaHCO_3_) were purchased from MACKUIN company (China). cis-1,3-Dibenzyl-2-oxo-4,5-imidazolidinedicarboxylic acid (H_2_L) was purchased from Tixiai (TCI) company (China). 5-dimethyl-1-pyrrolin-n-oxide (DMPO) and 2,2,6,6-tetramethyl-4-piperidone hydrochloride (TEMP) were purchased from DOJINDO LABORATORIES (Japan). N,N-dimethylformamide (DMF), methyl alcohol (MeOH), ethyl alcohol, sodium nitrate (NaNO_3_), nitric acid (HNO_3_), hydrofluoric acid (HF) and sulfuric acid (H_2_SO_4_) were obtained from Shenzhen Chang Tai Chemical Co., Ltd. (China). Deionized (DI) water with a resistivity of 18.2 MΩ cm^–1^ was produced by Milli-Q EQ 7008, and used in the synthesis of catalysts and batch experiments.

**Section S2: Synthesis of MIL-88A, MIL-100(Fe), ZIF-67 and BUC-92**

**MIL-88A:** The mixed solution with FeCl_3_·6H_2_O (5 mmol), fumaric acid (5 mmol) and 50 mL DMF was stirred in a 100 mL Teflon-lined for 1 h, then heated at 100 ℃ for 12 h. The prepared MIL-88A powder was washed twice with DMF and absolute ethanol [1].

**MIL-100(Fe):** 110.0 mg iron powder (Fe^0^) was dissolved in the mixed solution with 0.15 mL HNO_3_ and 10.0 mL H_2_O in a 25 mL Teflon-lined and ultrasound for 15 min. Then, 285 mg 1,3,5-Benzenetricarboxylic acid and 0.18 mL HF were added and heated at 150 ℃ for 24 h. The prepared MIL-100(Fe) was obtained by centrifugation and washed several times with deionized water and ethanol [2].

**ZIF-67:** 5 mmol of Co(NO_3_)_2_·6H_2_O and 20 mmol of 2-methylimidazole were respectively dispersed into the 80 mL methanol solution, then ultrasonic treatment for 10 min. Following that, the resultant mixture was put into a beaker to be stirred at room temperature for 24 h. Finally, the obtained purple powder was washed with methanol three times and dried at 60 ℃ over 12.0 h.

**BUC-92:** 71.38 mg CoCl_2_·6H_2_O, 106.31 mg H_2_L, 1 mL DMF and 14 mL deionized water were sealed in a 25 mL Teflon-lined and heated at 170 ℃ for 12 h. After cooling to ambient temperature, the resultant BUC-92 was washed with deionized water and dried at 60 ℃ over 12 h [3].

**Section S3: Characterizations**

X-ray single-crystal data collection toward PKU-24 was performed by Bruker D8 VENTURE with Mo Kα radiation (λ = 0.71073 Å) at 293(2) K. The SMART software was selected to collect crystal data, and the SAINT software was used for data extraction. Empirical absorption correction was carried out using the SADABS program. The structure was solved using Olex2 with the SIR2004 structure solution program using Direct Methods and refined with the ShelXL refinement package using Least Squares minimization. Crystallographic information has been deposited in the Cambridge Crystallographic Data Centre under the accession codes CCDC 2424707 (PKU-24). Powder X-ray diffraction (PXRD) patterns were recorded on a Bruker D8 diffractometer in the range of 2*θ* = 5°-50° with Cu *K_α_* radiation. The morphology of the materials was observed by scanning electron microscopy (SEM) (SU8020, Hitachi Limited, Japan), Tecnai G2 F20 high-resolution transmission electron microscopy (HR-TEM) and JEM GRAND ARM 300F (AC-HAADF-STEM). X-ray photoelectron spectroscopy (XPS) was measured using a Thermo ESCALAB 250XI. The metal ions leaching was detected by an inductively coupled plasma optical emission spectrometer (ICP-OES). Total organic carbon (TOC) was determined with a TOC analyzer (SHIMADZU TOC-L CPH). Electron spin resonance (ESR) spectra were obtained by EPR200-Plus instrument (CIQTEK Co., Ltd.) using 5,5-dimethyl-1-pyrroline-N-oxide (DMPO) and 2,2,6,6-Tetramethylpiperidine (TEMP) as spin-trapping agents to detect SO_4_^•−^, ^•^OH, O_2_^•−^ and ^1^O_2_.

**Section S4: Electrochemical Measurements**

All the electrochemical measurements were performed using an electrochemical workstation (CHI 760E, Shanghai Chenhua, China). The precursor suspension of the working electrode was prepared by sonication of 5.0 mg catalyst powder, 230.0 μL ethanol, 230.0 μL ultrapure water, and 40.0 μL Nafion solution (10 wt%). Then, the 40.0 μL turbid liquid was spread on the ITO with an area of 1.0 cm × 1.0 cm and dried it before use. The Pt electrode and Ag/AgCl electrode were used as a counter and reference electrode, respectively. Amperometric *i*-*t* curve and the open circuit potential were all measured in a 0.1 M Na_2_SO_4_ solution.

**Section S5: In-situ Fourier-Transform Infrared (FTIR) Measurement**

The in-situ FTIR spectra were performed using the INVENIO S spectrometer (Bruker). All of spectra were scanned with a wave number range of 4000-500 cm^–1^ at a resolution of 4 cm^–1^. During in-situ reaction, 2 mL of organic pollutants aqueous solution (TC, BPA and SMX) was added to the reaction tank, and tested the spectrum. Subsequently, 3 mg of PKU-24 catalyst was introduced to perform the reaction. After 3 min, 50 μL PMS solution was dropped and recorded until the reaction lasted for 6 min. All the spectra had subtracted the water as the background.

**Section S6: X-ray Absorption Fine Structure (XAFS) Data Analysis**

The XAFS data were processed according to the standard procedures using the Athena module implemented in the IFEFFIT software packages. The EXAFS spectra were obtained by subtracting the post-edge background from the overall absorption and then normalizing with respect to the edge-jump step. Subsequently, the χ(k) data were Fourier transformed to real (*R*) space using a Hanning windows (d_k_ = 1.0 Å^−1^) to separate the EXAFS contributions from different coordination shells. To obtain the quantitative structural parameters around central atoms, least-squares curve parameter fitting was performed using the ARTEMIS module of IFEFFIT software packages.

**Section S7: Data Analysis and Test Methods**

The residual concentrations of TC, CTC, OTC, SMX, SDZ, SIX, BPA, BPS, BPE, CIP, OFX, AN, PN, DCP, CBZ, ATZ, BA, NBA, PMSO and PMSO_2_ were tested by the ultra–high performance liquid chromatography (UHPLC, Thermo Scientific Vanquish Flex) equipped with a multi-wavelength UV detector. The separation was performed on a C18 reversed-phase column (2.1 mm × 100 mm, 1.7 μm). The column temperature was 25 °C. The detail detection methods were provided in **Table S1**.

The kinetics (*k*_obs_) of the microcontaminants elimination data were fitted by first-order equation which given as Eq. S1:

$ln(C_{t}/C_{0})=-k_{obs}\times t$ (S1)

Where *C_t_* was the concentration of pollutants at t time (mg L^-1^), C_0_ was the original concentration of contaminants (mg L^-1^), *k*_obs_ was the degradation rate constant (min^-1^).

The PMS residual concentration was determined using the modified KI method [4, 5]. In detail, 10.0 μL Filtrate was mixed with 10.0 mL KI solution (50.0 mM). Then, the mixed solution was stirred for enough time to ensure the complete reaction between PMS and I^−^, which further generated the yellow-colored I_3_^−^. The I_3_^−^ concentration, proportional to PMS, was determined at *λ*_max_ = 352 nm by the UV-vis spectrophotometer.

**Section S8: Density Functional Theory (DFT) Calculations**

Various electronic properties like density of states (DOS), partial density of states (PDOS), electron density, and spin states were used to analyze the electronic structure of various catalysts. Adsorption energy, PDOS, electron density difference and Bader charge population were calculated to investigate the interaction between the catalyst and PMS. The adsorption energy (*E*_ads_) of PMS was defined as Eq. S2:

$E_{\mathrm{ads}}=E_{PMS/surf}-E_{\mathrm{surf}}-E_{\mathrm{PMS}}$ (S2)

Where, *E*_PMS/surf_, *E*_surf_ and *E*_PMS_ are the energy of PMS adsorbed on the surface, the energy of a clean surface, and the energy of an isolated PMS atom in a cubic periodic box, respectively.

The change of Gibbs free energy for PMS activation was calculated by the Eq. S3:

$\Delta G=\Delta E+\Delta ZPE-T\Delta S$ (S3)

Where, Δ*E* was the binding energy, Δ*ZPE* was the difference in zero-point energies, and Δ*S* was the change of entropy, respectively. The Gibbs free energies of the reaction were modified by the contribution of the vibrational frequencies of the surface molecules to ΔZPE and entropy.

**Section S9: Molecular Dynamics (MD) Simulations**

All MD simulations were performed using the open-source software package LAMMPS [6] and atomic structures were visualized using OVITO [7]. The OPLS all-atom force field was used to model interactions among the cyclohexanone molecules [8].

$E\left( r^{N} \right)=E_{bonds}+E_{angles}+E_{dihedrals}+E_{nonbonded}$ (S4)

$E_{bonds}=\sum_{bonds} K_{r}{(r-r_{0})}^{2}$ (S5)

$E_{angles}=\sum_{angles} K_{\theta}{(\theta-\theta_{0})}^{2}$ (S6)

$E_{dihedrals}=\sum_{dihedrals} (\frac{V_{1}}{2}\left[ 1+\cos\left( \Phi-\Phi_{1} \right) \right]+\frac{V_{2}}{2}\left[ 1-\cos\left( 2\Phi-\Phi_{2} \right) \right]+\frac{V_{3}}{2}\left[ 1+\cos\left( 3\Phi-\Phi_{3} \right) \right]+\frac{V_{4}}{2}\left[ 1-\cos\left( 4\Phi-\Phi_{4} \right) \right]$ (S7)

$E_{nonbonded}=\sum_{i>j} f_{ij}(\frac{A_{ij}}{r_{ij}^{12}}-\frac{C_{ij}}{r_{ij}^{6}}+\frac{q_{i}q_{j}e^{2}}{4\pi\varepsilon_{0}r_{ij}})$ (S8)

where *r*_ij_ is the interparticle separation between particles i and j, and σ and ε are characteristic length and energy scales of the Lennard-Jones interaction.

The system was first energy-minimized via the gradient descent approach. The Nose-Hoover thermostat [9, 10] was used to maintain the equilibrium temperature at 300 K and periodic boundary conditions were imposed on all three dimensions. The Particle Mesh-Ewald method [11] was used to compute long-range electrostatics within a relative tolerance of 1 × 10^-8^. A cut-off distance of 1nm was applied to real-space Ewald interactions. Equilibrium molecular dynamics (EMD) simulations were performed at 300 K to obtain the mass density profiles of TC molecules along the nanochannels.

**Section S10: Life Cycle Perspective Calculations**

This study employs the ReCiPe Midpoint (H) analysis method in Simapro software, combined with the Intergovernmental Panel on Climate Change (IPCC) analysis method, to systematically quantify the environmental emissions generated by different Fenton-like systems throughout their life cycles. The ReCiPe Midpoint (H) method covers key environmental impact indicators, including climate change, stratospheric ozone depletion, human toxicity, fine particulate matter formation, ionizing radiation, photochemical ozone formation, terrestrial and aquatic eutrophication, terrestrial acidification, water resource depletion, mineral and fossil resource depletion, and land use change. Additionally, this study utilizes the IPCC analysis method, which focuses on the contribution of greenhouse gas emissions to global warming potential. The synergistic application of these two methods enables a comprehensive investigation of the environmental impacts of Fenton-like reactions, providing data and theoretical support for subsequent optimization.


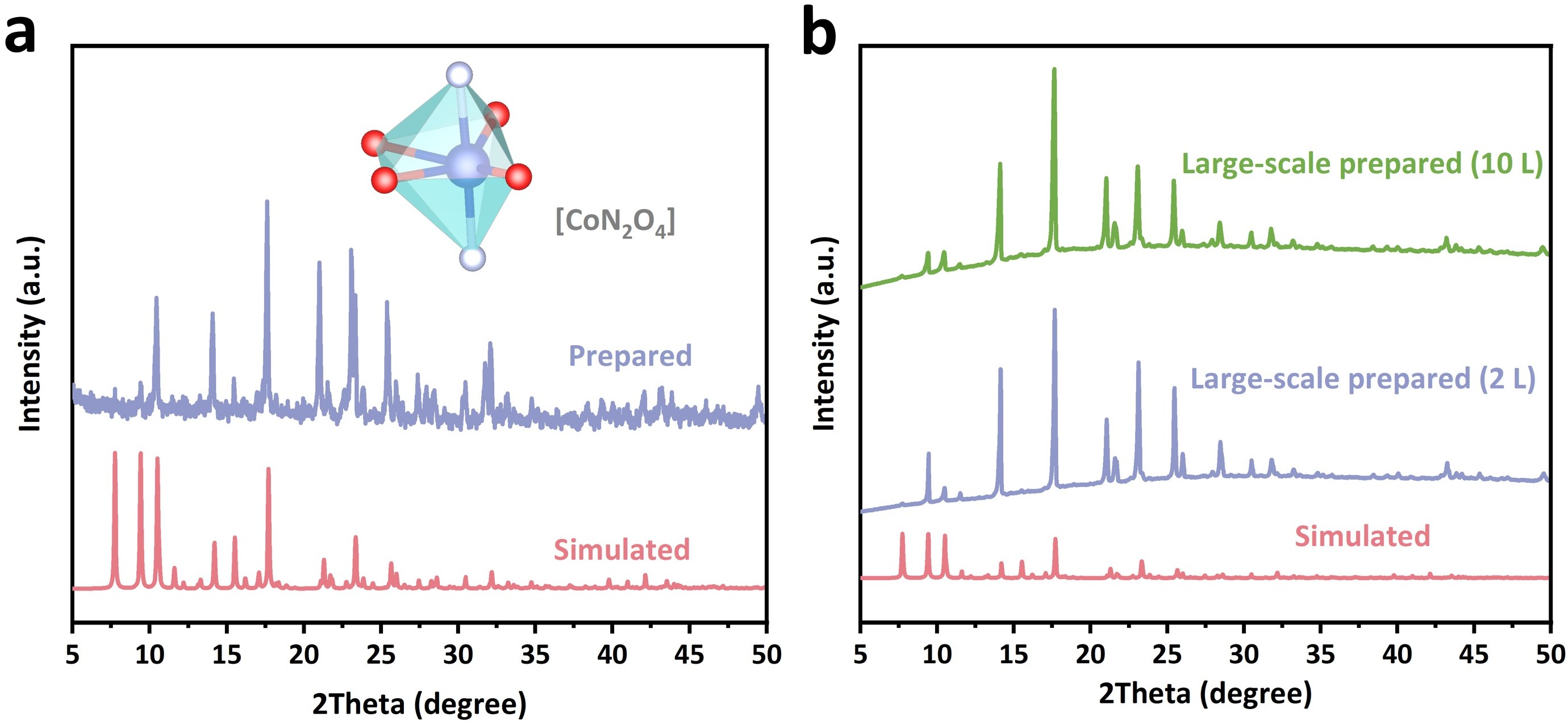


**Figure S1.** PXRD pattern of (a) pure PKU-24 and (b) large-scale synthesized PKU-24 in Teflon lined autoclave (2 L) or high borosilicate glass container (10 L).


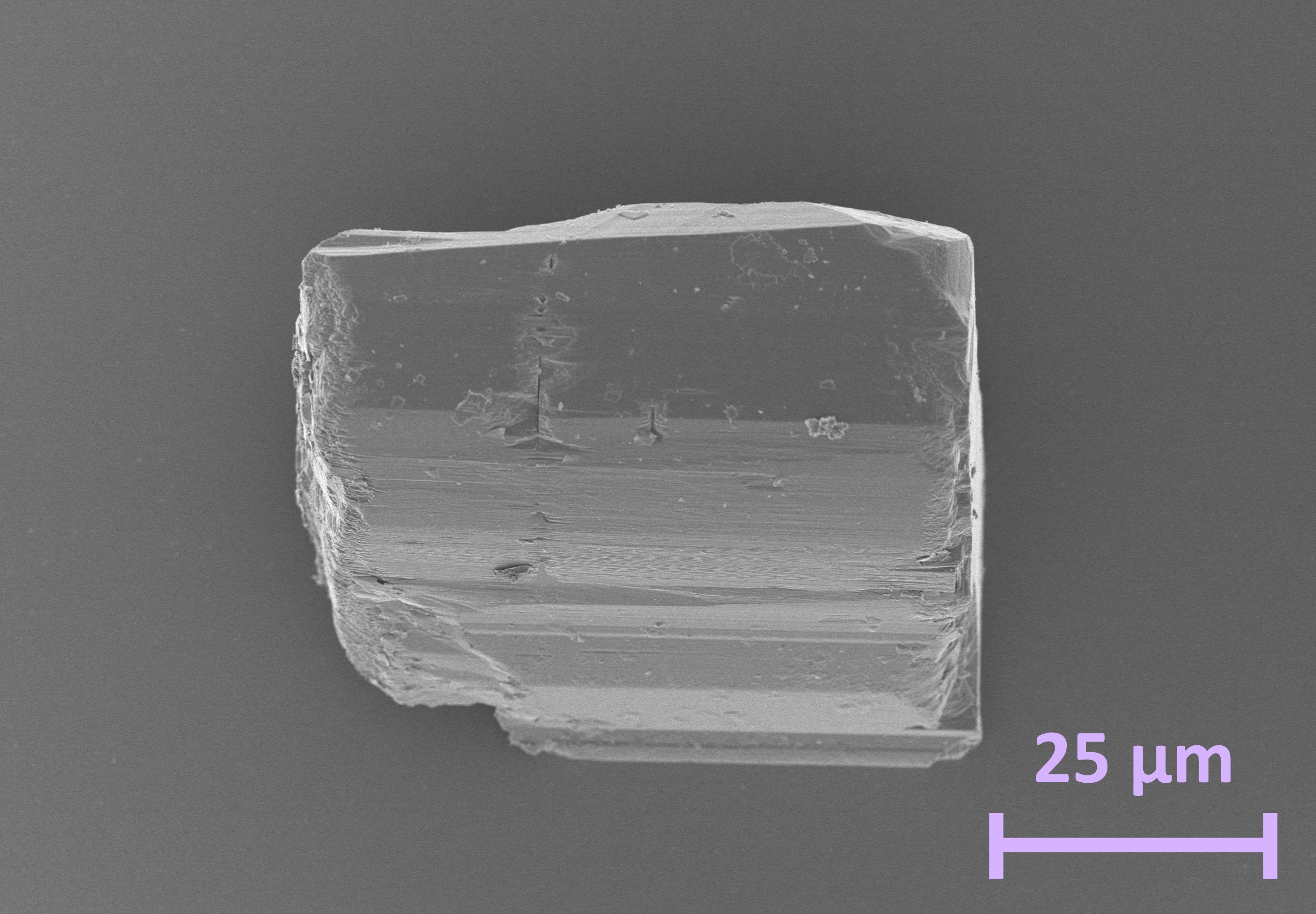


**Figure S2.** The SEM image of pure PKU-24.


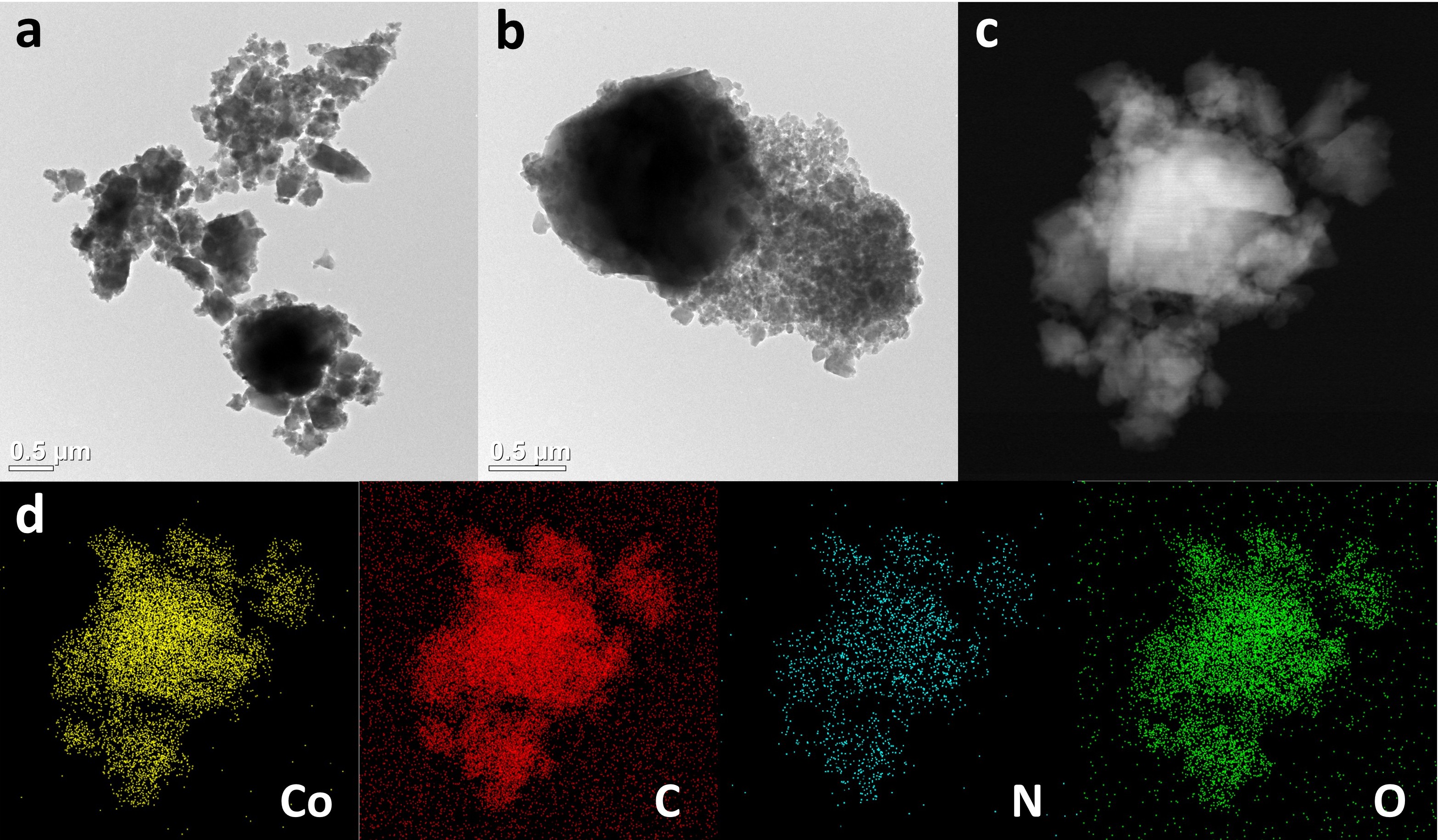


**Figure S3.** (a-b) HR-TEM image and (c-d) corresponding elemental mapping of pure PKU-24.


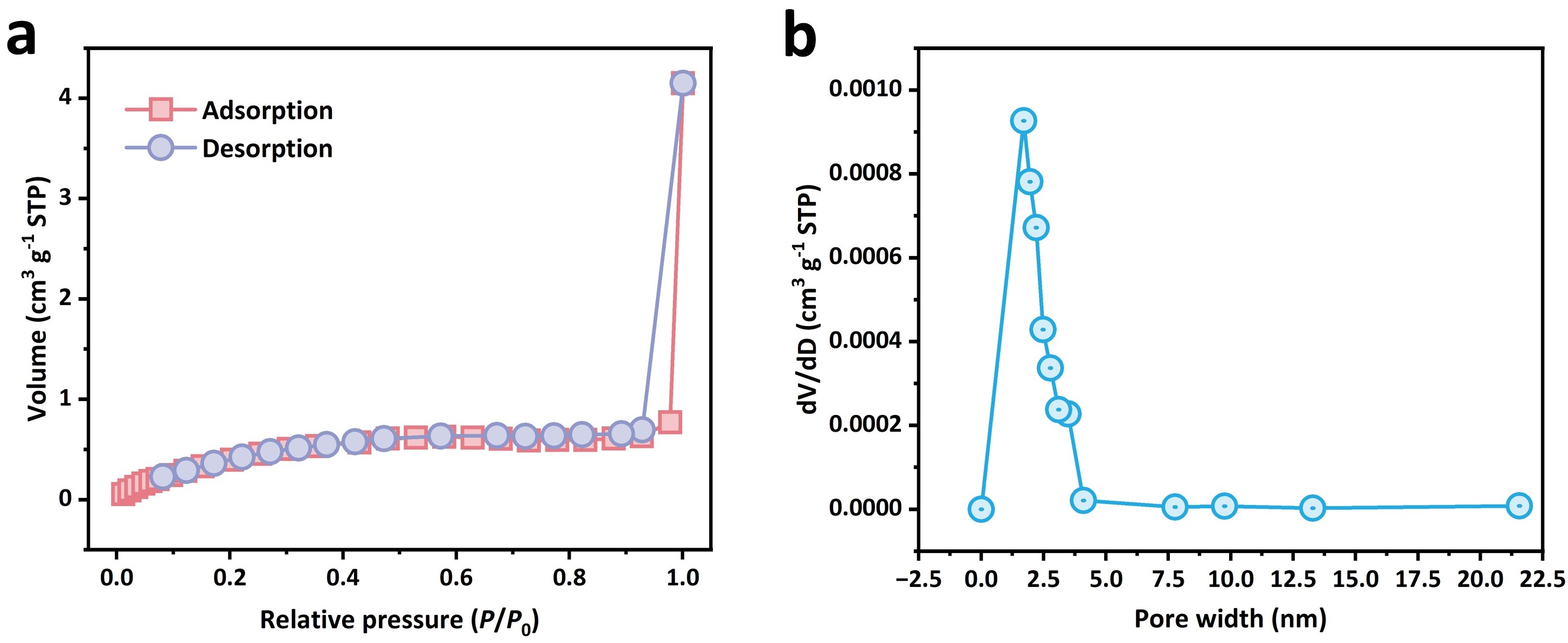


**Figure S4.** (a) The N_2_ adsorption-desorption isotherm and (b) the pore size distribution curves (the BJH desorption model) of pure PKU-24.


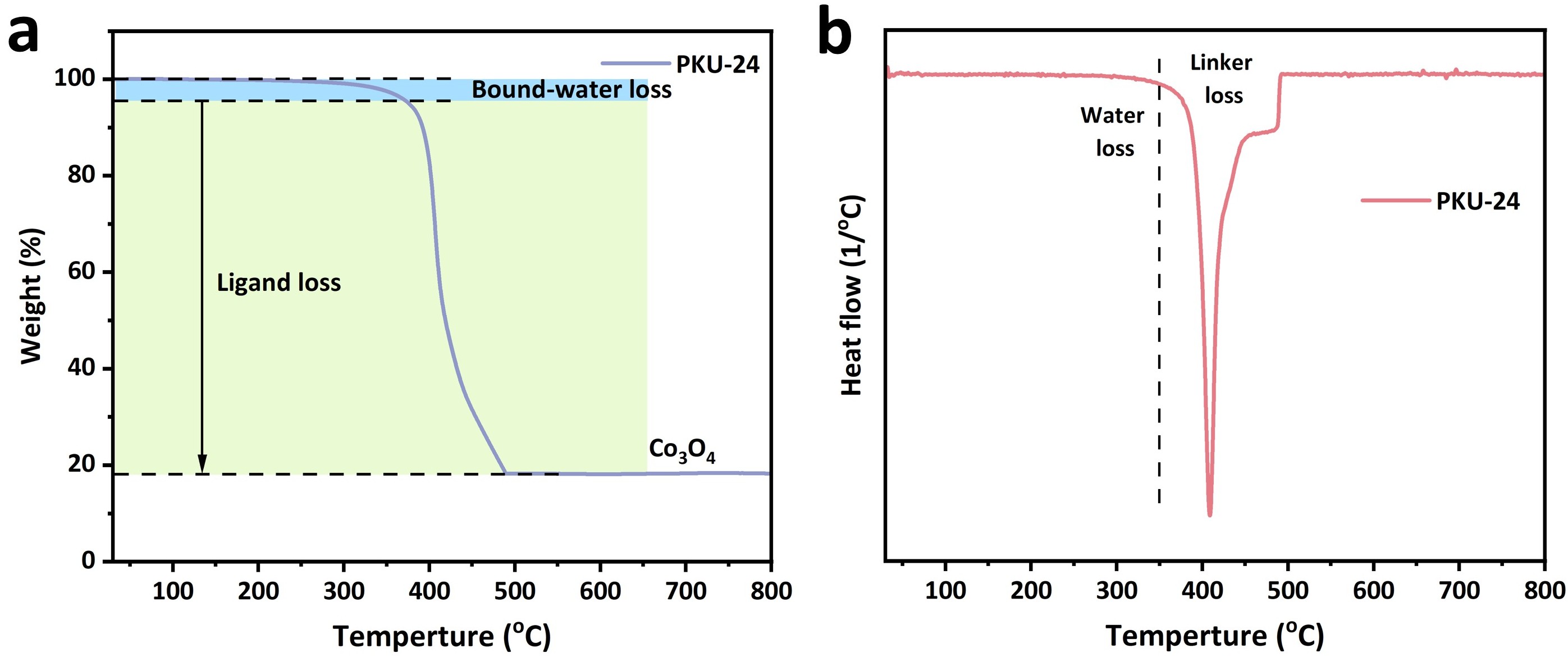


**Figure S5.** (a) The TGA curves and (b) the corresponding DSC thermogram of pure PKU-24.


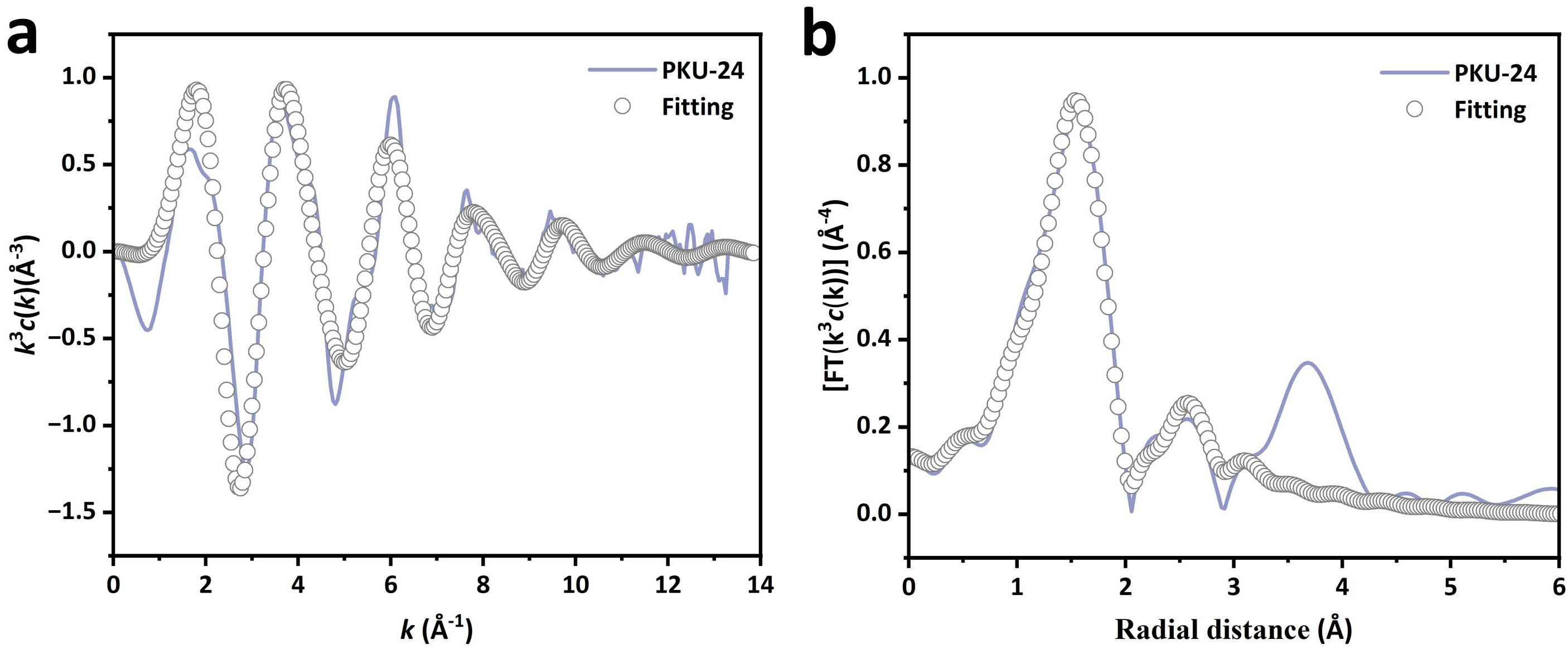


**Figure S6.** Fourier-filtered in (a) the k space and (b) the R space of pure PKU-24.


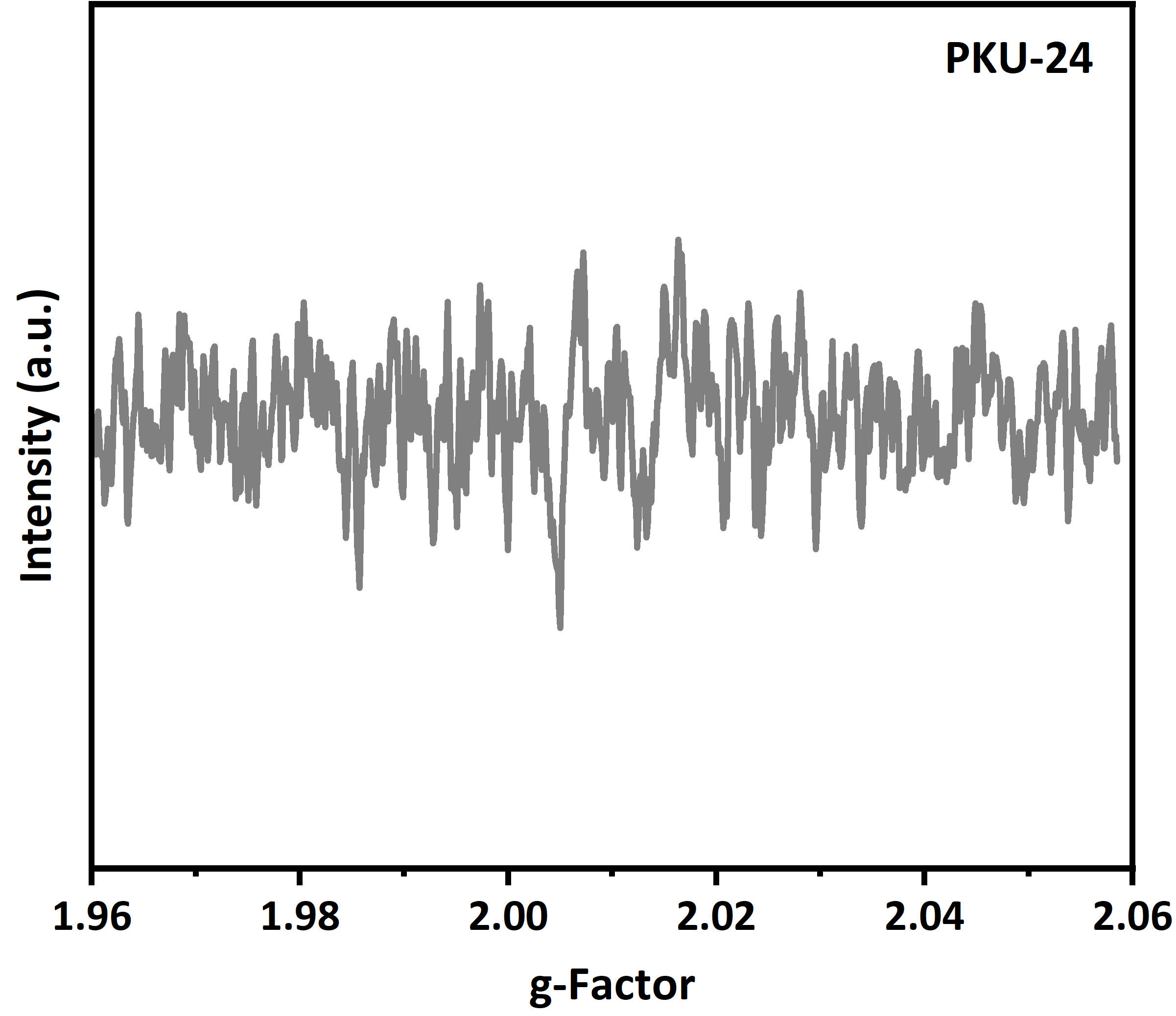


**Figure S7.** EPR spectrum of PKU-24.


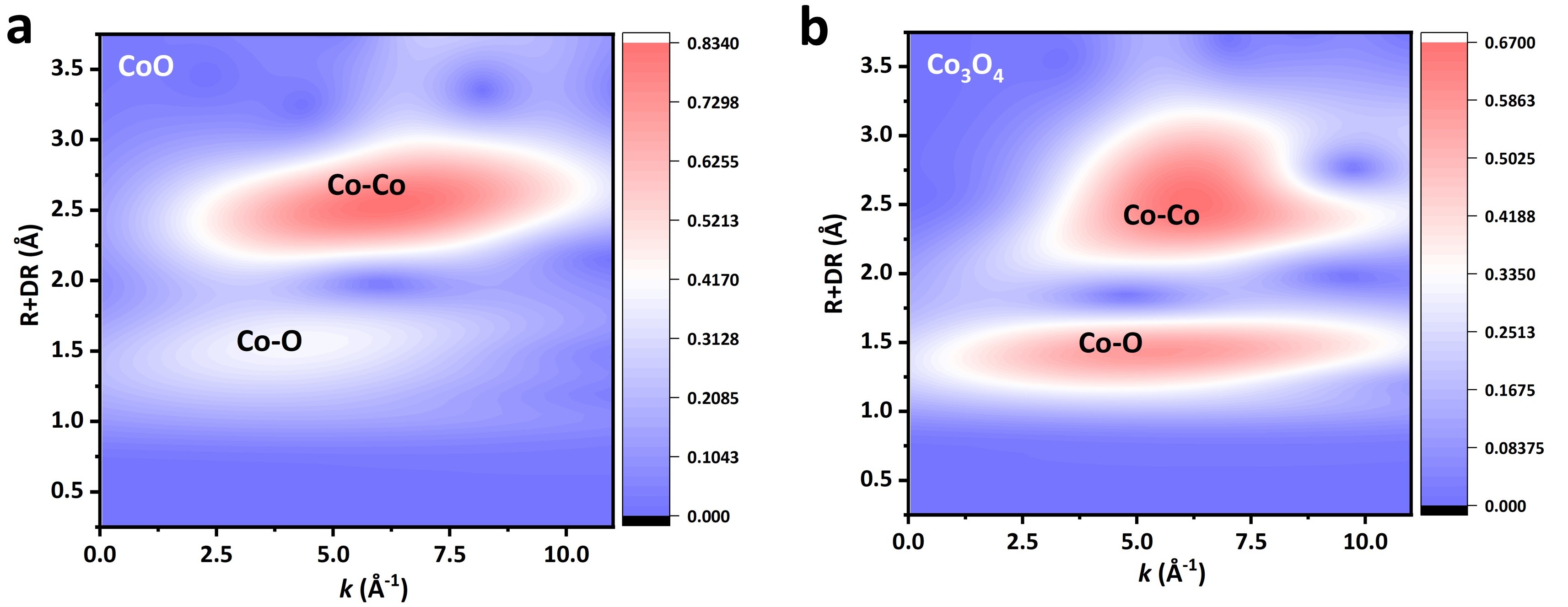


**Figure S8.** The WT plots of the Co K-edge EXAFS for (a) CoO and (b) Co_3_O_4_.


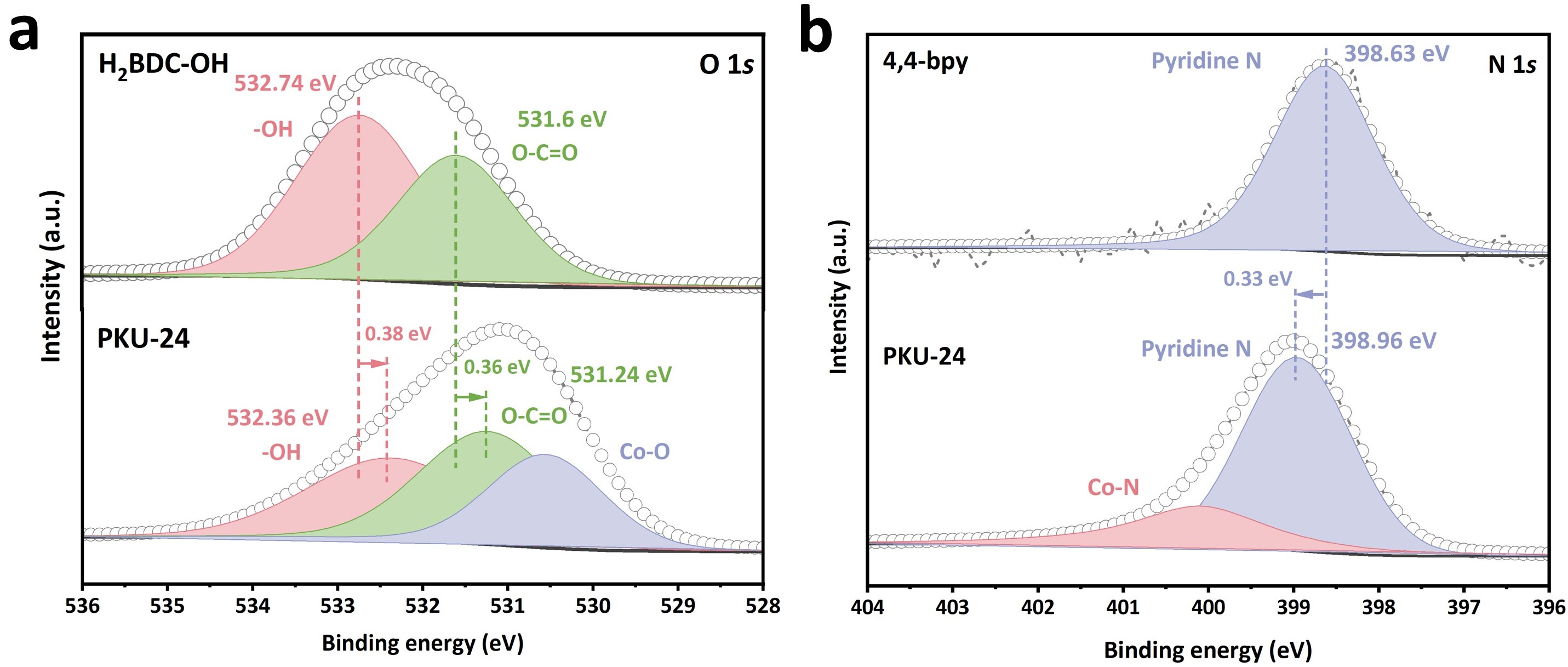


**Figure S9.** The XPS of (a) O 1*s* and (b) N 1*s* in pristine ligands and pure PKU-24.

As shown in **Figure S9b**, a shift of 0.33 eV to higher binding energy happened, indicating that partial electrons from pyridine N of 4,4-bpy were transferred to coordinate with Co atoms. And the Co-N bond could be observed in N 1*s* of PKU-24.


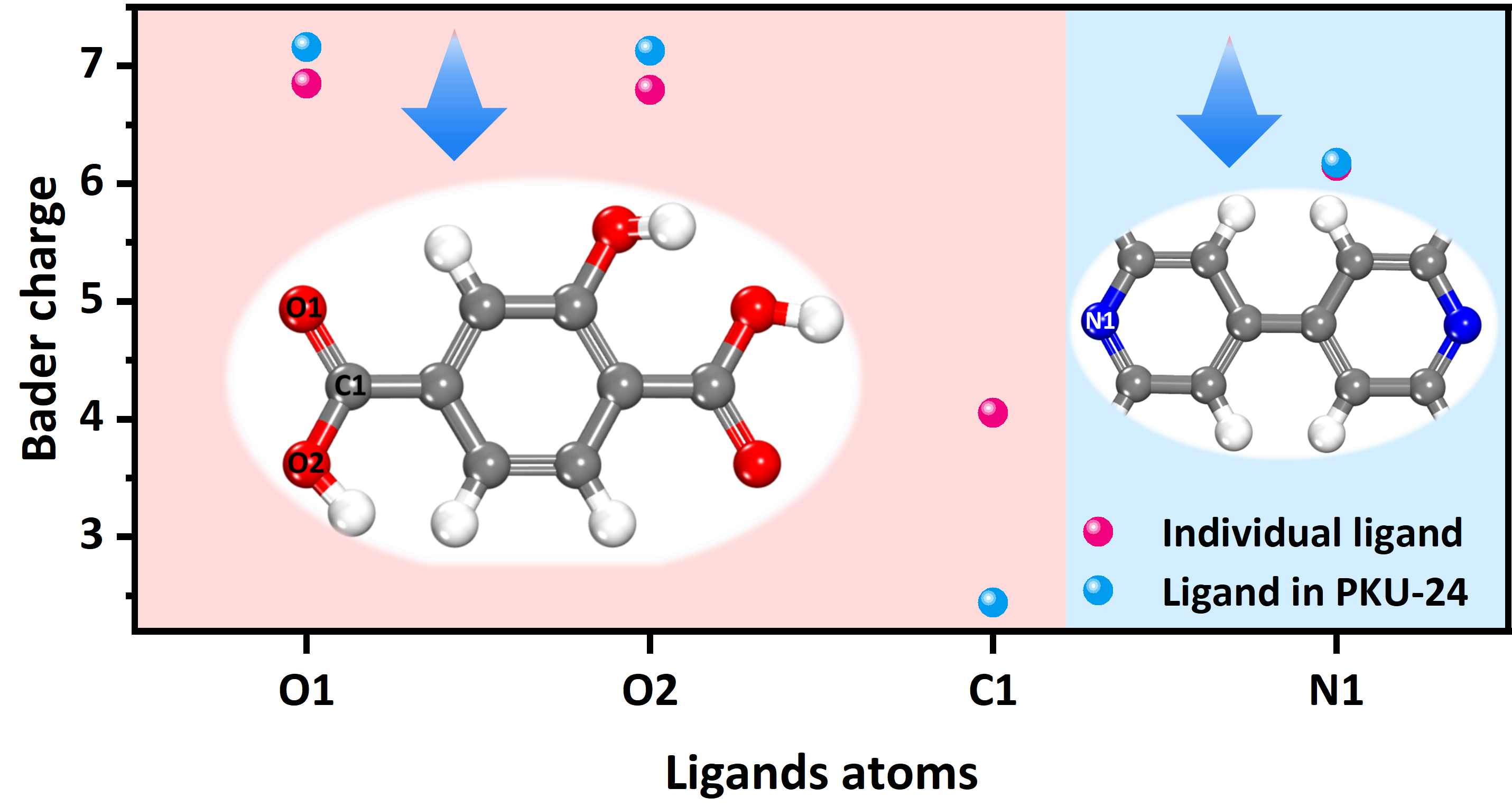


**Figure S10.** The change of the Bader charge of organic ligands before and after coordination in PKU-24.


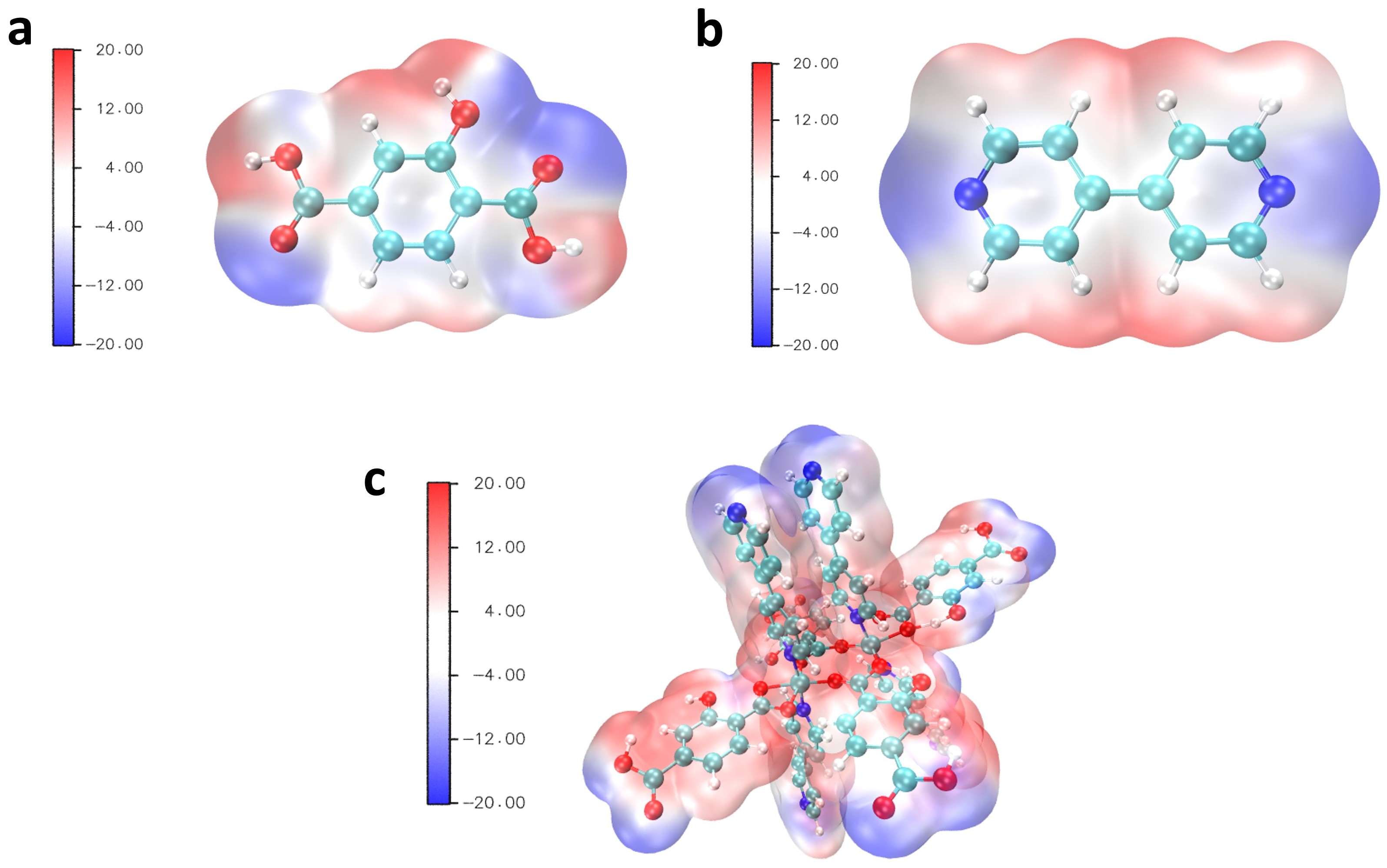


**Figure S11.** The electrostatic potential spectra of (a) 2-hydroxyterephthalic acid, (b) 4,4-bipyridine and (c) PKU-24 unit cell.


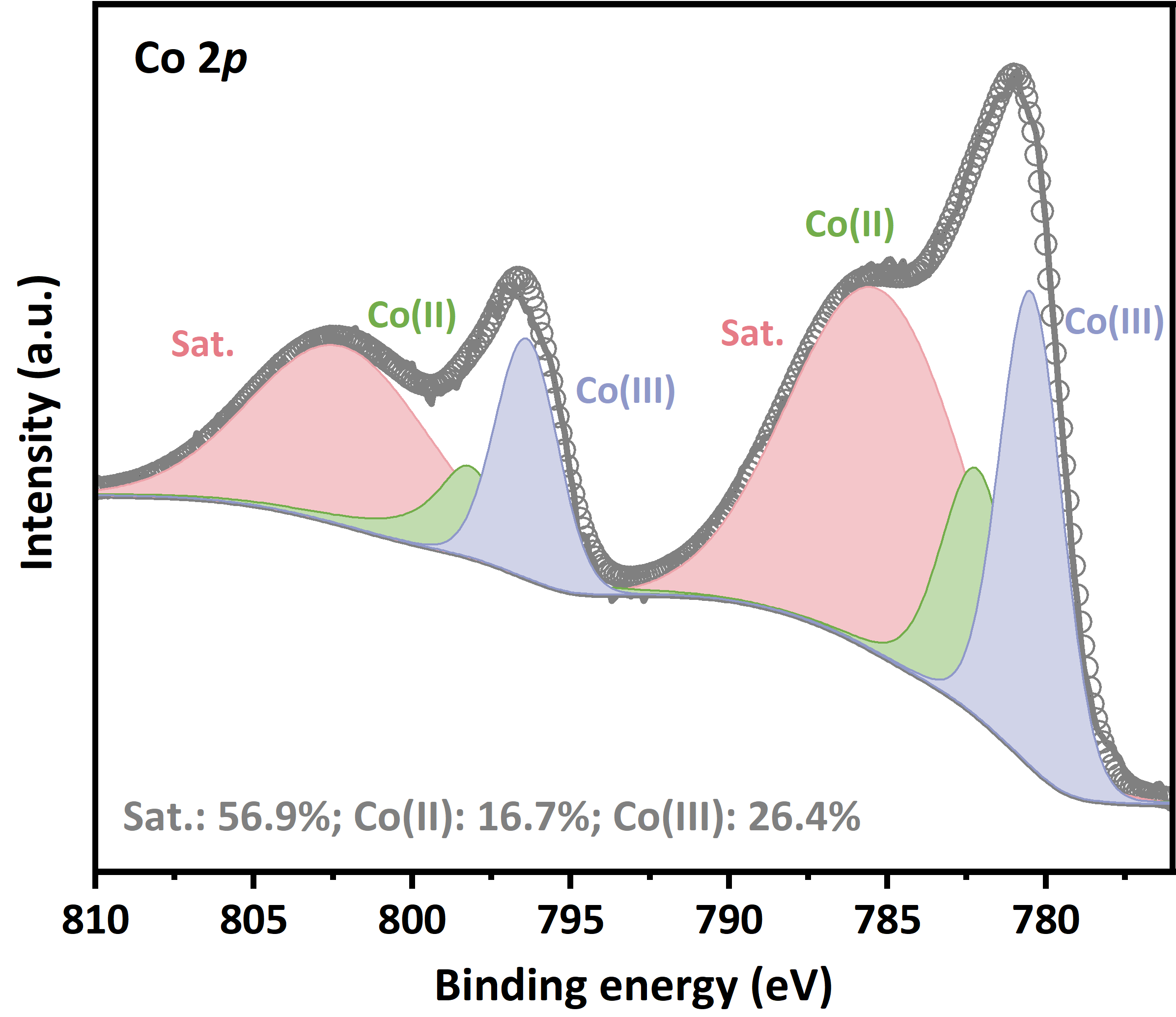


**Figure S12.** The XPS Co 2*p* spectrum analysis of pure PKU-24.


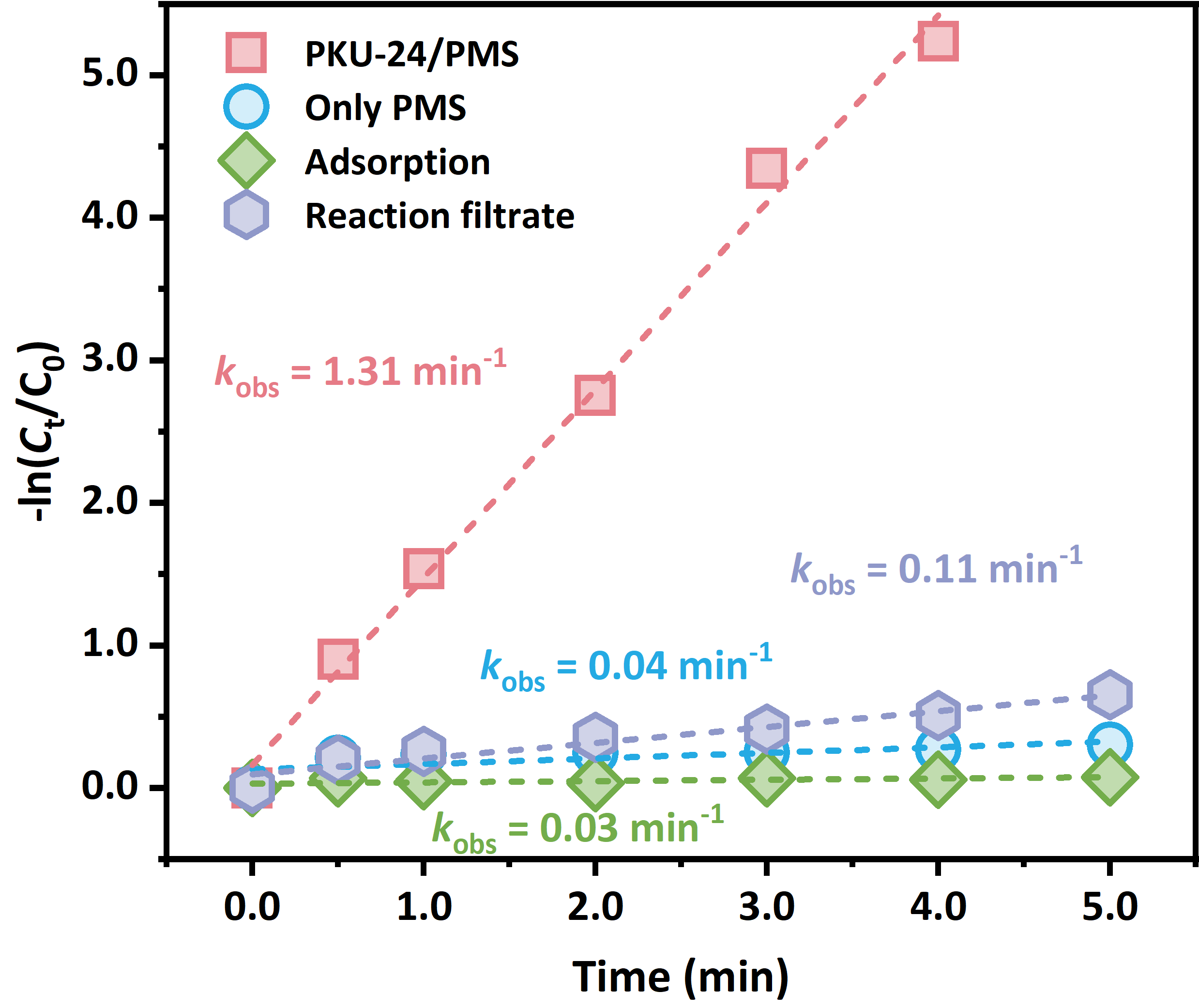


**Figure S13.** The first-order rate constants in various catalytic systems.

**Experimental conditions:** [Catalyst] = 0.2 g L^–1^, [PMS] = 0.2 mM, [TC] = 10.0 mg L^–1^, [Initial pH] = 6.03.


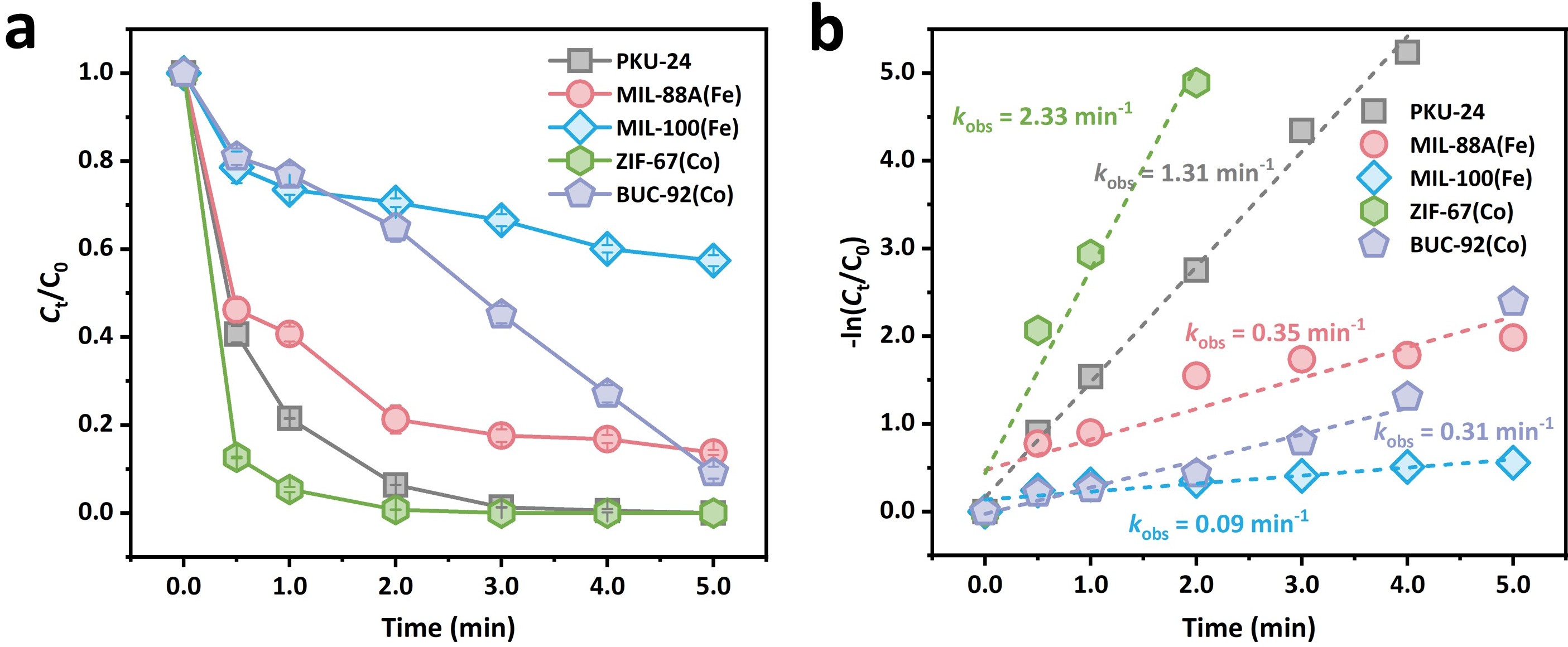


**Figure S14.** (a) The TC degradation activity tests over different MOFs and (b) corresponding first-order rate constants. The error bars in the figures represented the standard deviations from triplicate tests.

**Experimental conditions:** [Catalyst] = 0.2 g L^–1^, [PMS] = 0.2 mM, [TC] = 10.0 mg L^–1^, [Initial pH] = 6.03 (in a-b).

**Note:**

As shown in **Figure S14**, the *k*_obs_ of PKU-24/PMS was higher than that of MIL-88A(Fe), MIL-100(Fe) and BUC-92(Co), confirming its outstanding catalytic activity. Although the *k*_obs_ displayed by ZIF-67/PMS were faster than those of PKU-24/PMS, the leached Co ions (1.66 mg L^−1^) made a significant contribution to TC removal. Moreover, the water stability of ZIF-67 was poor in a weakly acidic environment, because the soft acid (Co^2+^)-soft base (2-MI) coordination could cause the H^+^ to compete with the ligand for coordination, leading to the collapse of the framework.


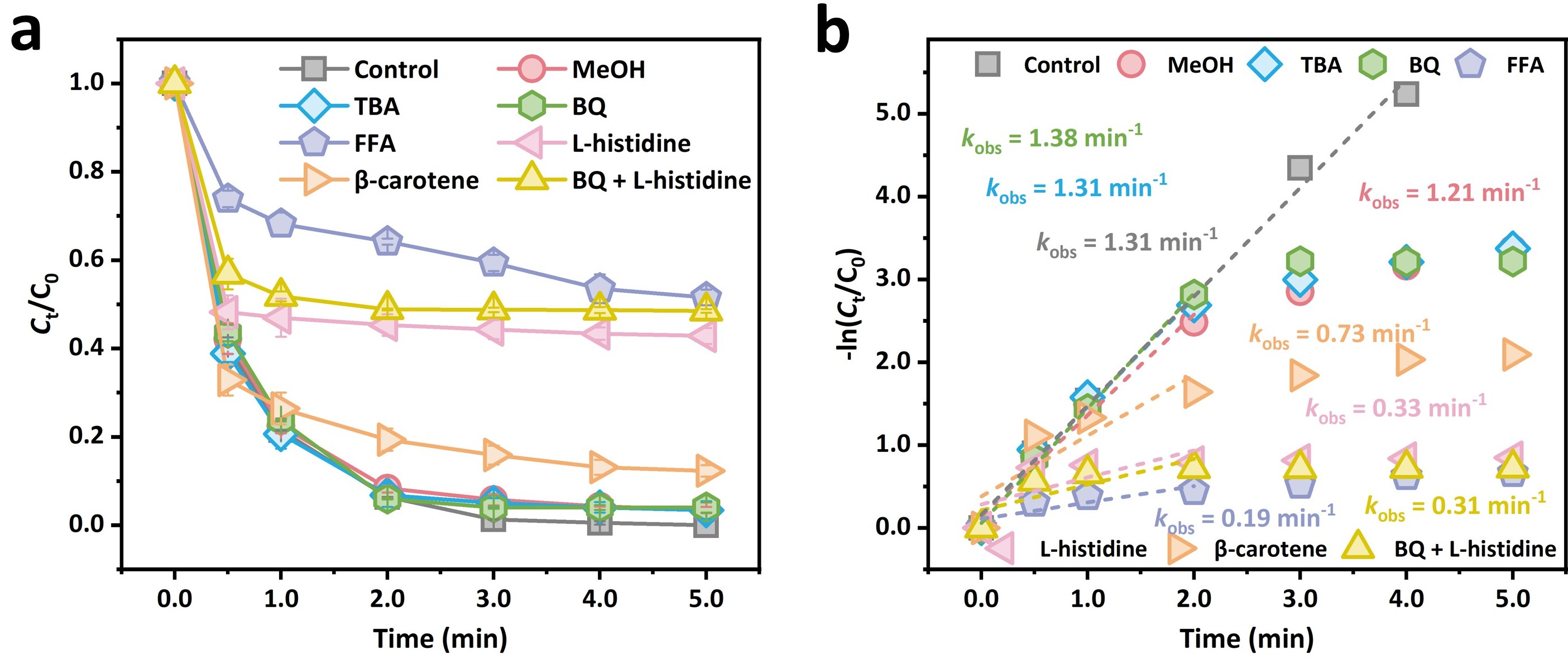


**Figure S15.** (a) The quenching experiments and (b) corresponding first-order rate constants in PKU-24/PMS system. The error bars in the figures represented the standard deviations from triplicate tests.

**Experimental conditions:** [Catalyst] = 0.2 g L^–1^, [PMS] = 0.2 mM, [TC] = 10.0 mg L^–1^, [MeOH] = [TBA] = 40.0 mM, [FFA] = [L-histidine] = 10.0 mM, [BQ] = [β-carotene] = 5.0 mM (in a-b).


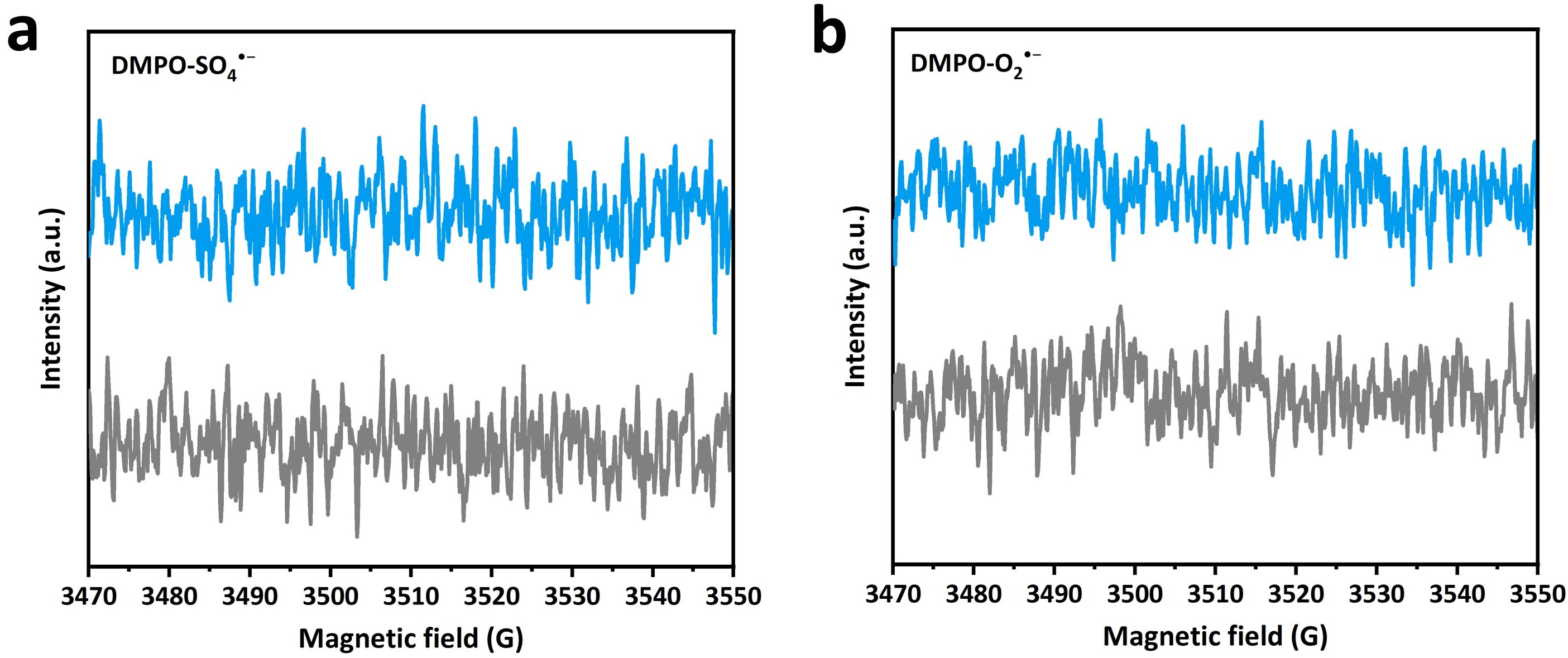


**Figure S16.** The EPR spectra of (a) SO_4_^•−^ and (b) O_2_^•−^ using DMPO as the trapping agent under different conditions.


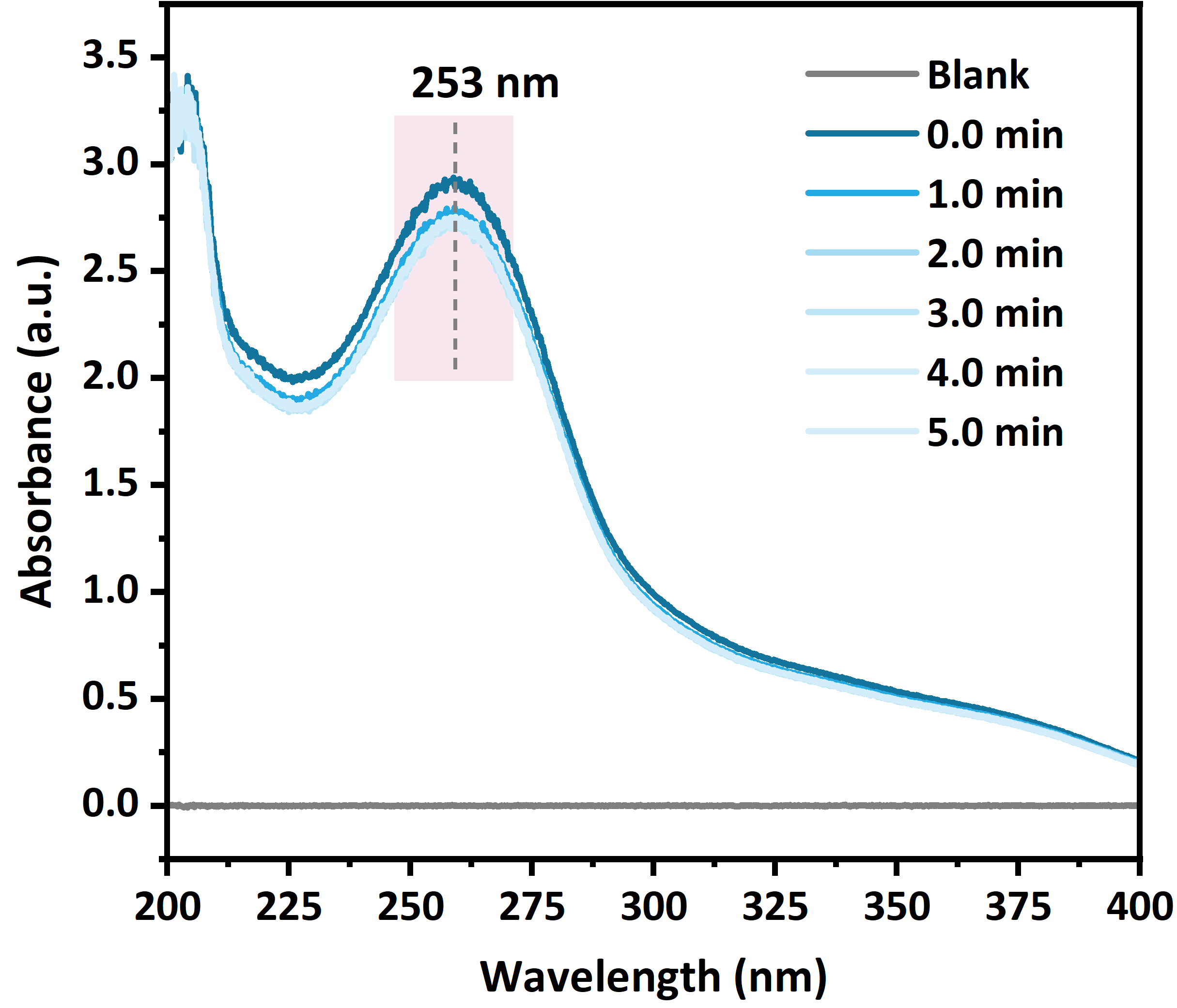


**Figure S17.** The UV absorption spectra of the O_2_^•−^ measurement system (259 nm was the measurement wavelength of NBT).


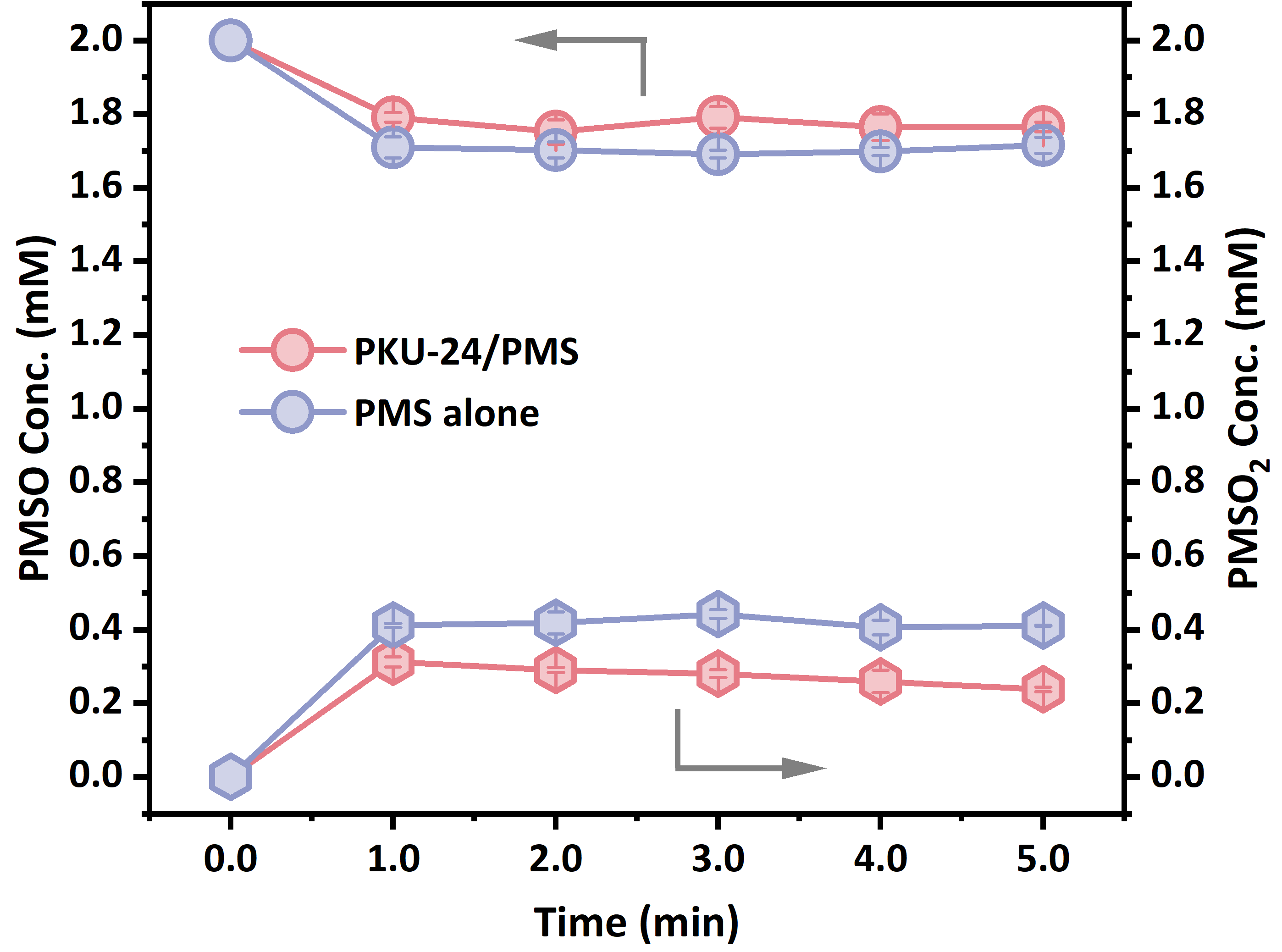


**Figure S18.** The elimination of PMSO and generation of PMSO_2_ in PKU-24/PMS and individual PMS systems. The error bars in the figures represented the standard deviations from triplicate tests.

**Experimental conditions:** [Catalyst] = 0.2 g L^–1^, [PMS] = 0.2 mM, [PMSO] = 2.0 mM, [Initial pH] = 6.03.


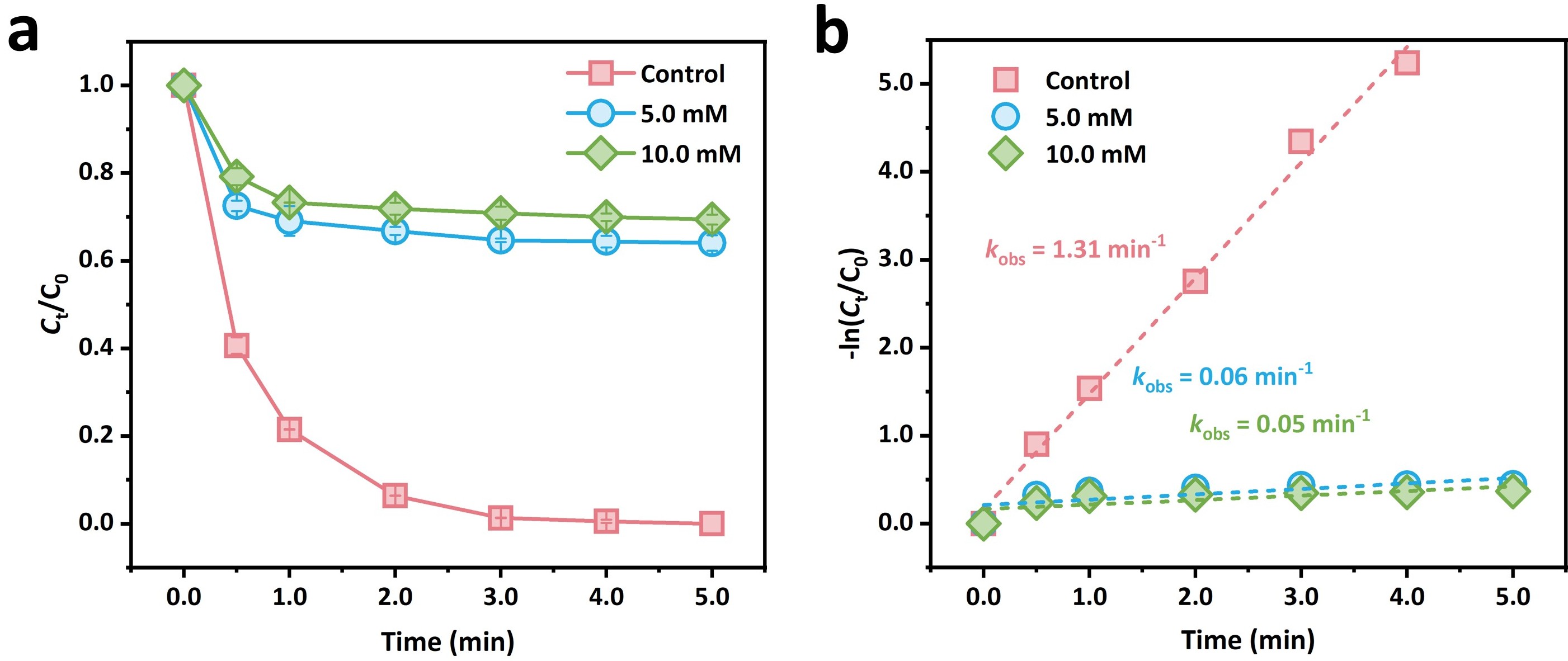


**Figure S19.** Influences of NO_2_^−^ with different concentrations on (a) TC removal efficiencies and (b) corresponding first-order rate constants in PKU-24/PMS system. The error bars in the figures represented the standard deviations from triplicate tests.

**Experimental conditions:** [Catalyst] = 0.2 g L^–1^, [PMS] = 0.2 mM, [TC] = 10.0 mg L^–1^ (in a-b).


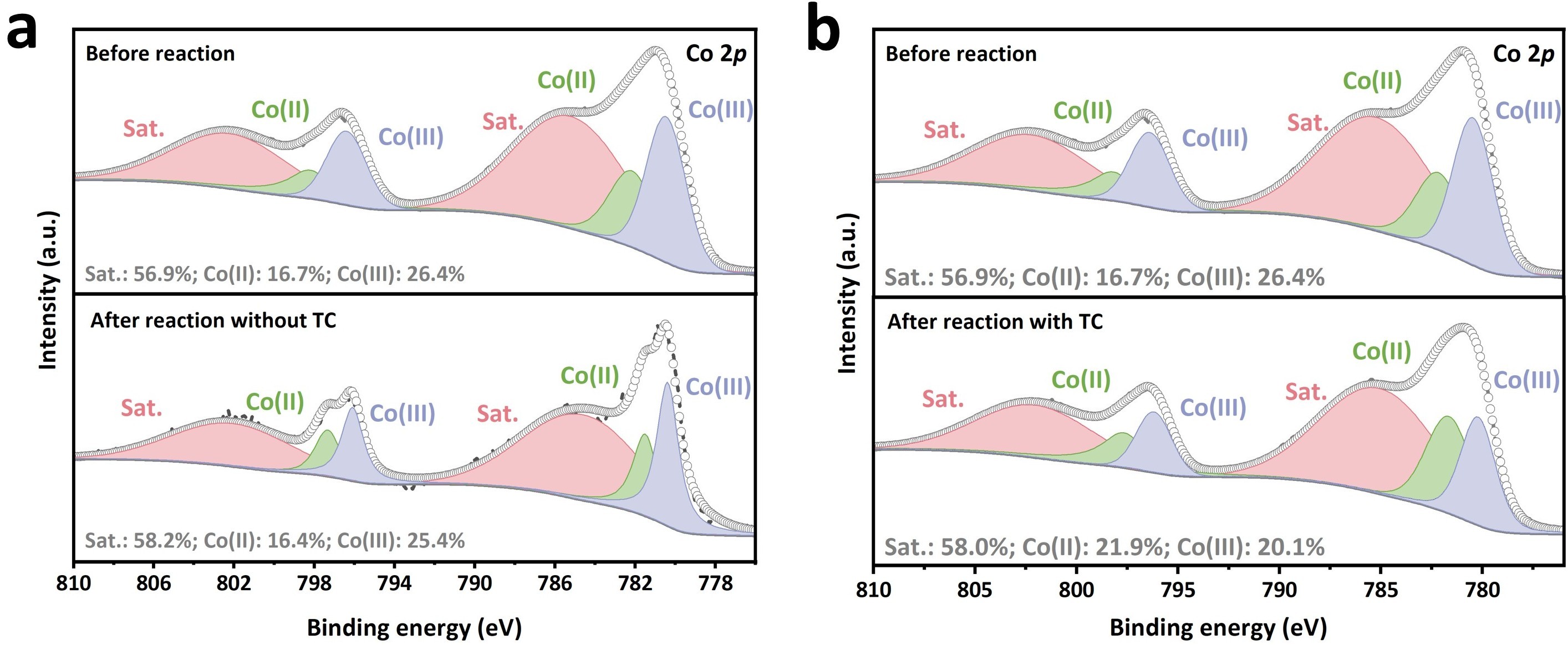


**Figure S20.** The XPS of Co 2*p* in PKU-24 before and after reaction (a) without TC or (b) with TC.


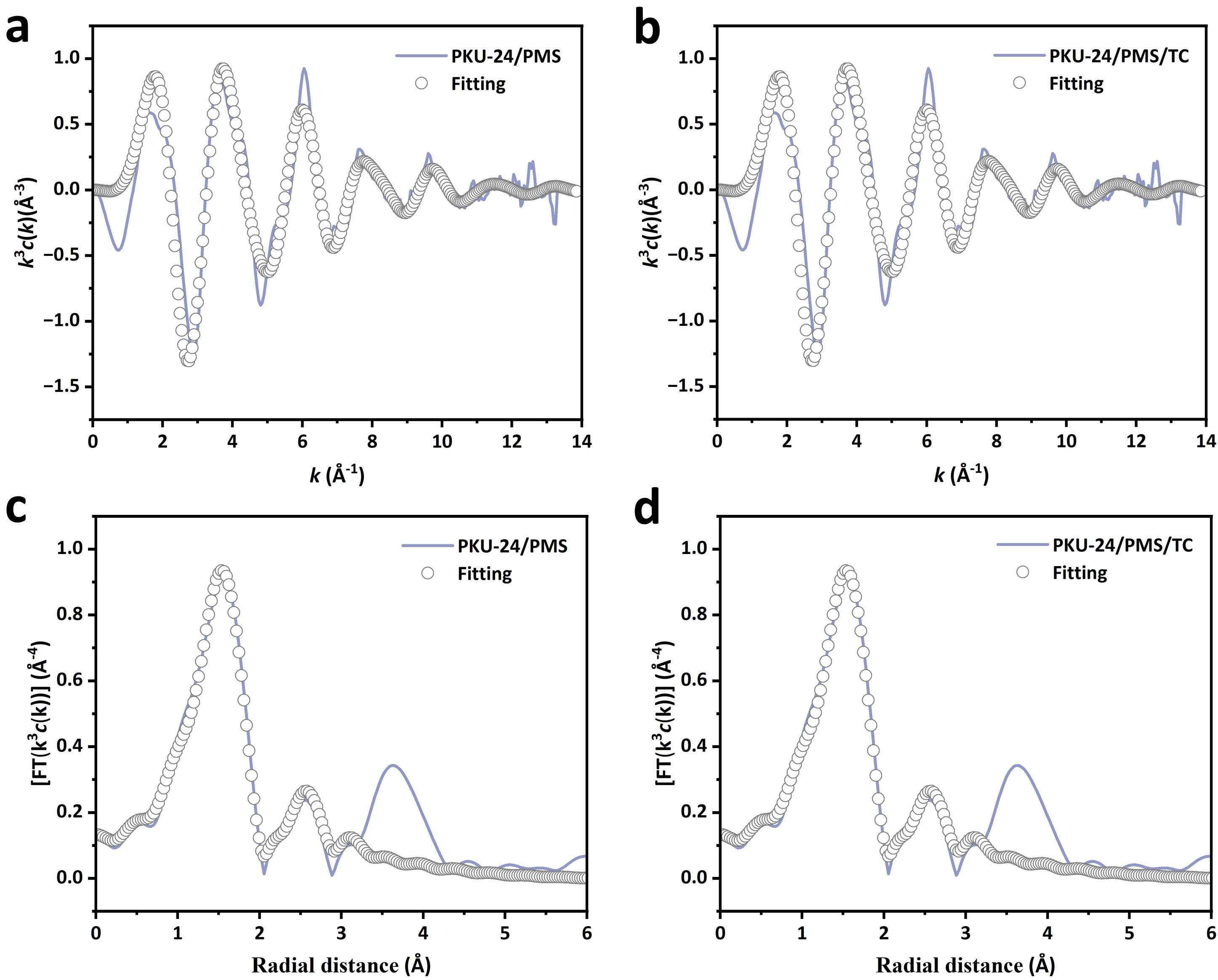


**Figure S21.** Fourier-filtered in (a-b) the k space and (c-d) the R space of PKU-24/PMS and PKU-24/PMS/TC.


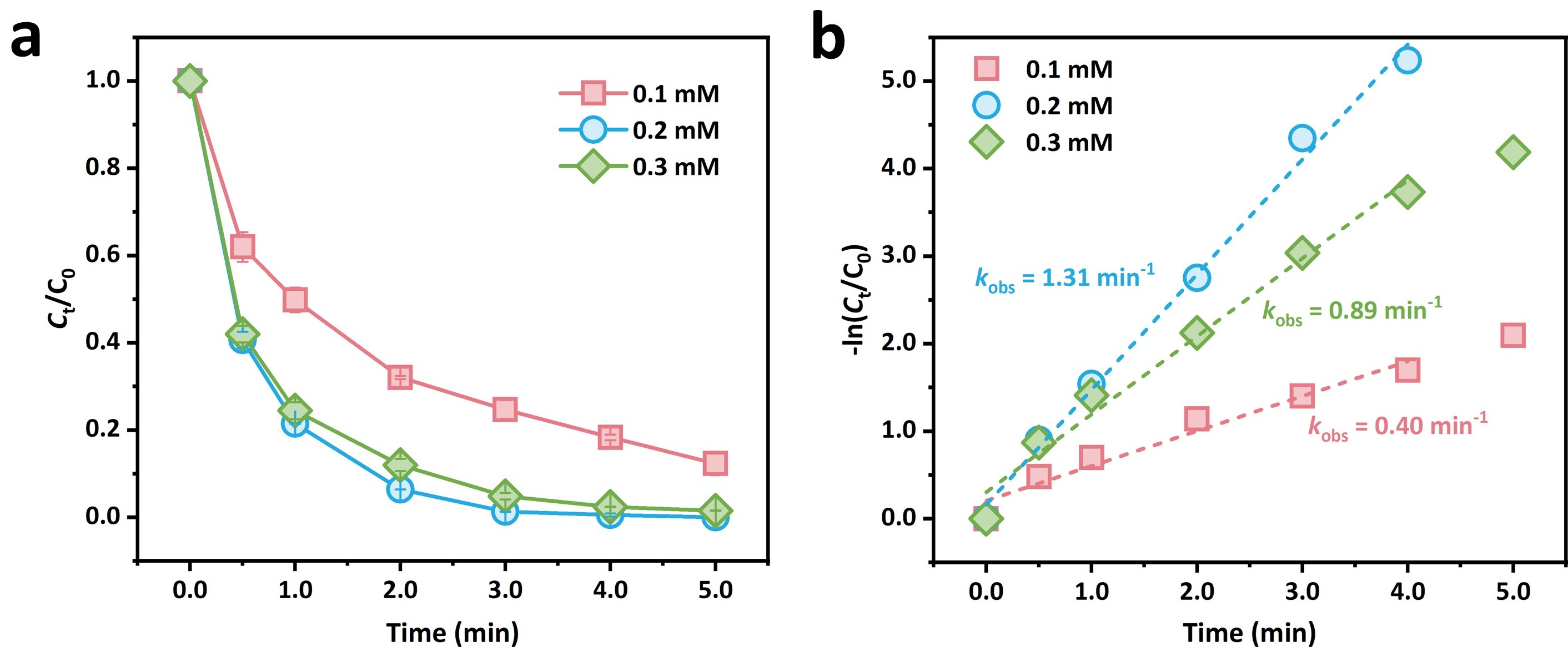


**Figure S22.** (a) Influence of PMS concentrations on TC elimination efficiencies and (b) corresponding first-order rate constants in PKU-24/PMS system. The error bars in the figures represented the standard deviations from triplicate tests.

**Experimental conditions:** [Catalyst] = 0.2 g L^–1^, [PMS] = 0.2 mM, [TC] = 10.0 mg L^–1^, [Initial pH] = 6.03 (in a-b).


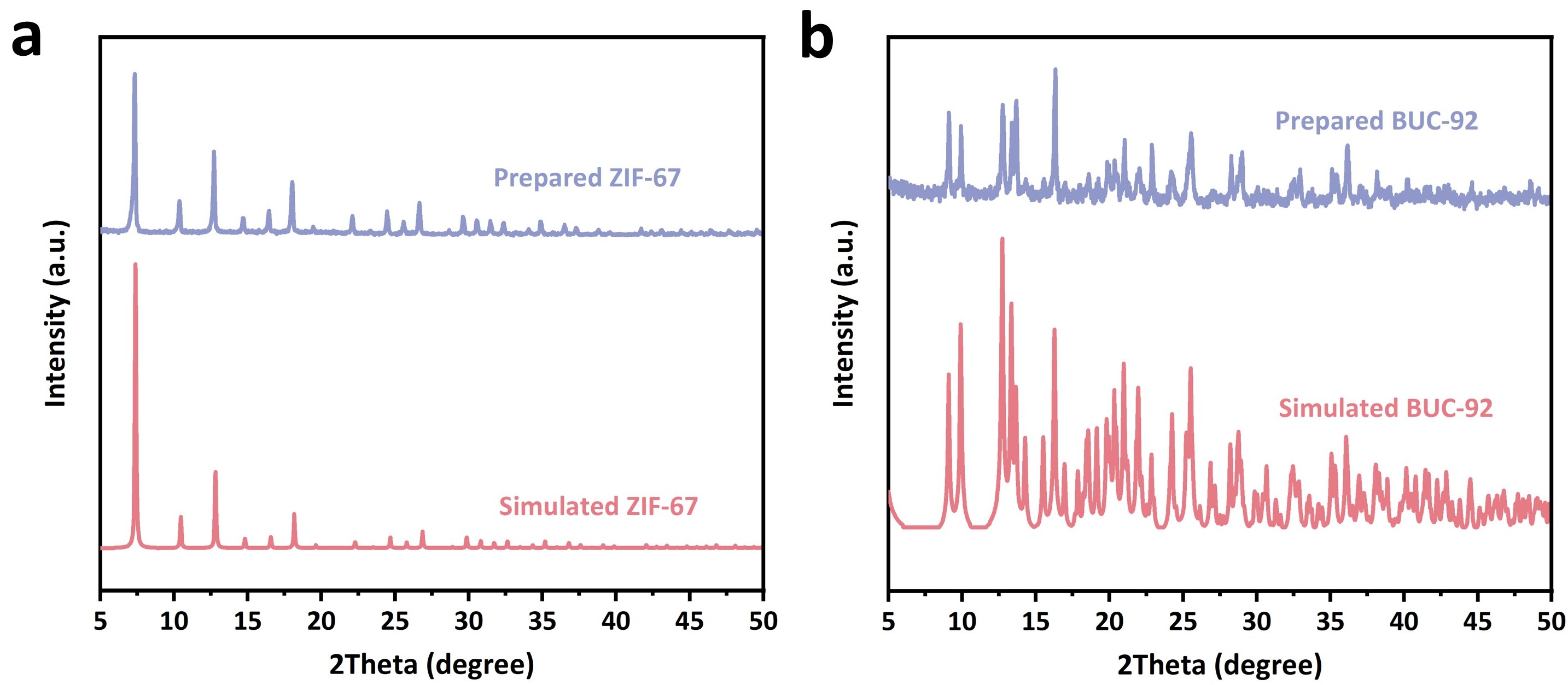


**Figure S23.** PXRD patterns of (a) ZIF-67, (b) BUC-92 and their simulated samples.


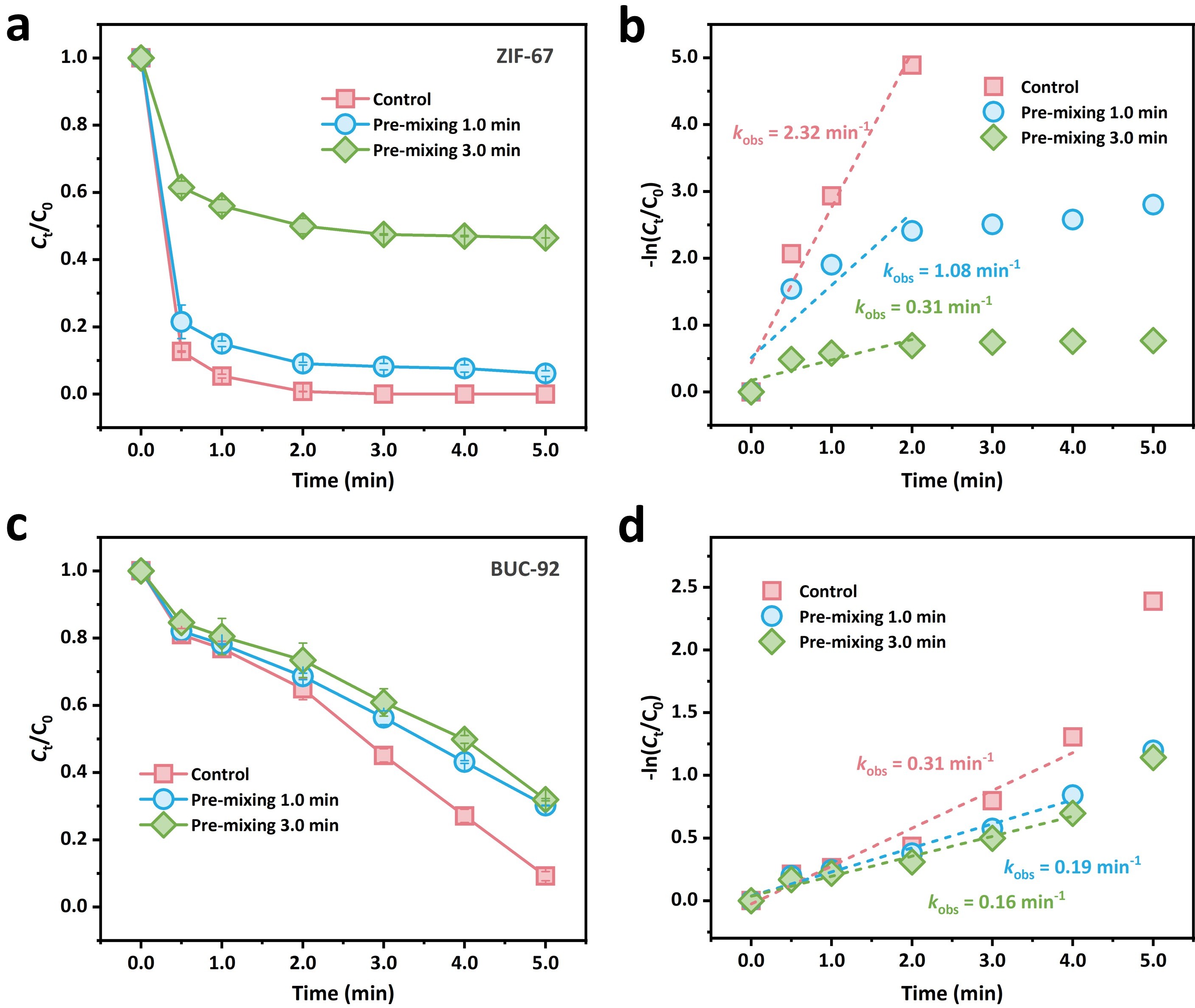


**Figure S24.** The TC removal efficiencies after PMS mixing with (a, b) ZIF-67 and (c, d) BUC-92 in advance (1.0 min or 3.0 min) as well as corresponding first-order rate constants. The error bars in the figures represented the standard deviations from triplicate tests.

**Experimental conditions:** [Catalyst] = 0.2 g L^–1^, [TC] = 10.0 mg L^–1^, [PMS] = 0.2 mM (in a-d).

**Note:**

**Figure S24** demonstrated that the pre-mixing of PMS and catalysts significantly influenced the TC degradation efficiencies and *k*_obs_ in ZIF-67/PMS and BUC-92/PMS, indicating that they could all directly activate PMS.


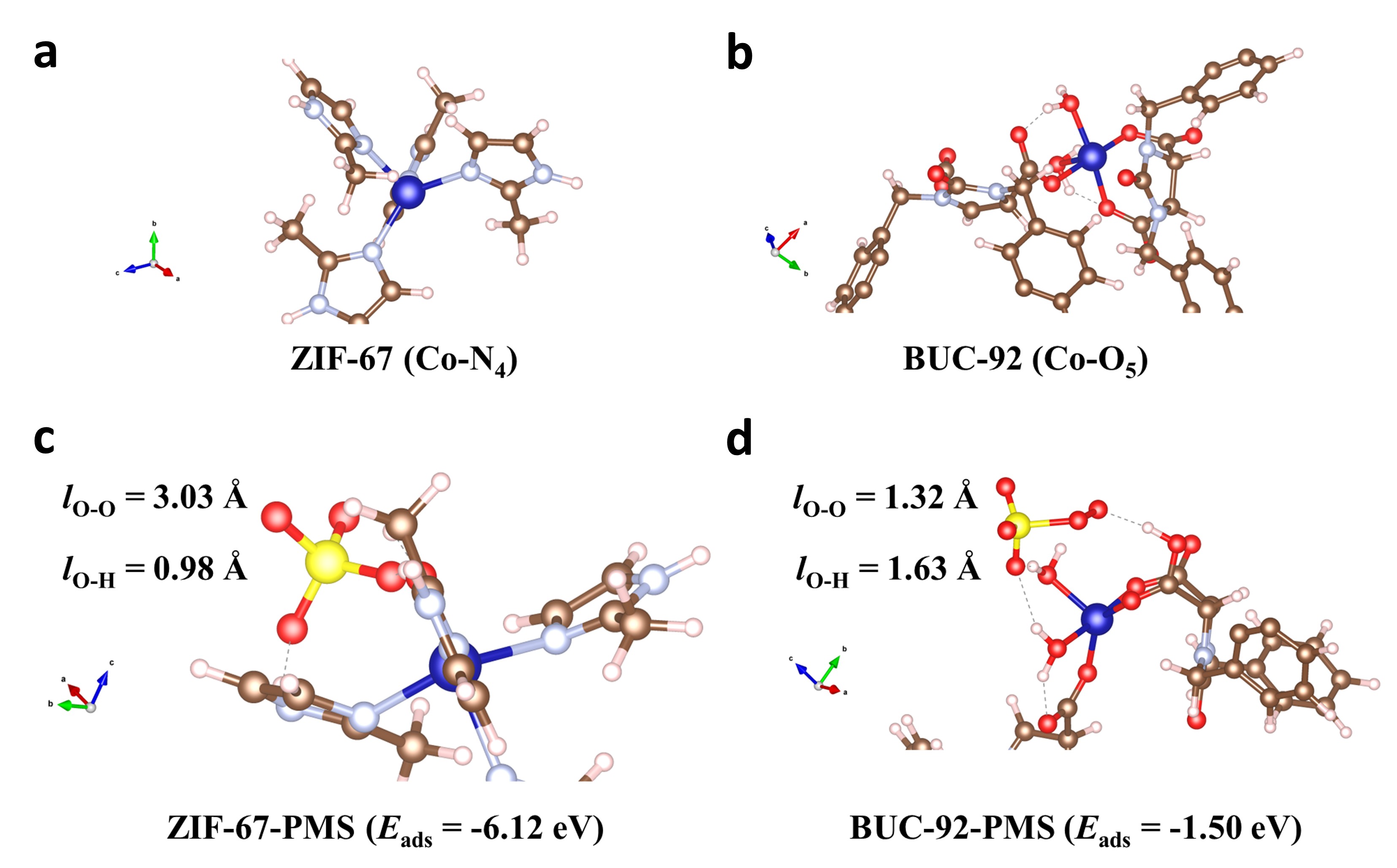


**Figure S25.** (a) ZIF-67 and (b) BUC-92 calculation models and (c-d) corresponding adsorption energy calculations.


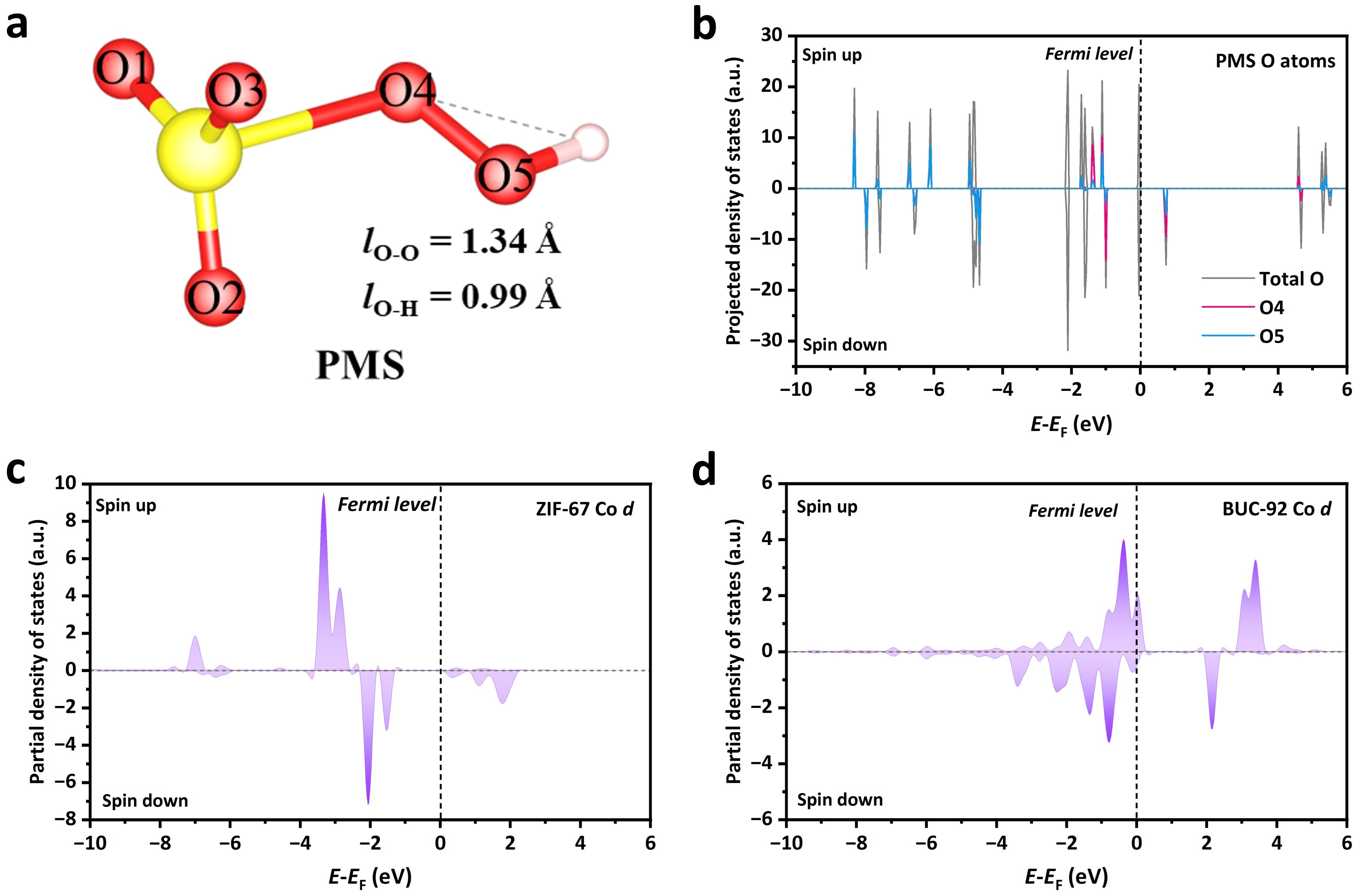


**Figure S26.** (a) PMS structural model. PDOS spectra of (b) PMS O *p*, (c) ZIF-67 Co *d* and (d) BUC-92 Co *d* orbitals.

**Note:**

As shown in **Figure S26**, BUC-92 preferentially coordinated with O atoms (O1, O2 or O3) from S-O bond. While ZIF-67 tended to interact with terminal O (O5) from PMS, attributing to that the matched energy level between Co 3*d* and O 2*p* orbitals would induce strong interaction.


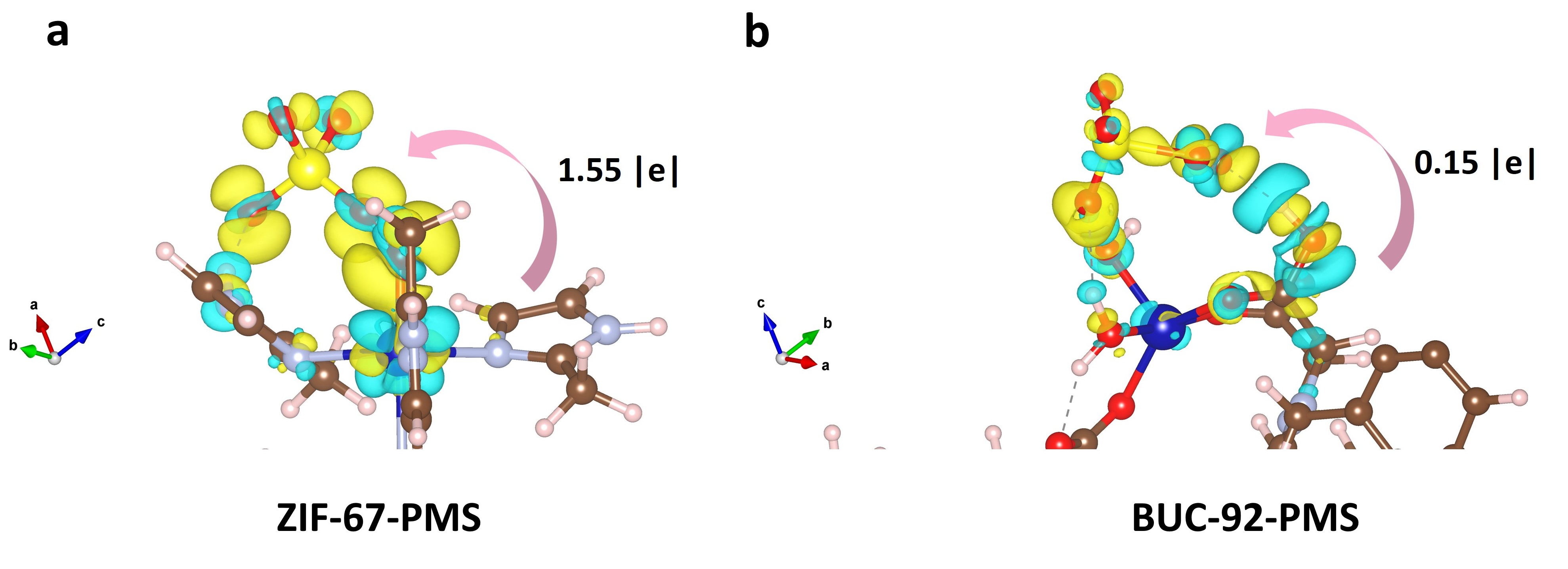


**Figure S27.** The electron density difference of (a) ZIF-67-PMS and (b) BUC-92-PMS models (yellow and blue represented the accumulation and dissipation of electron clouds, respectively).


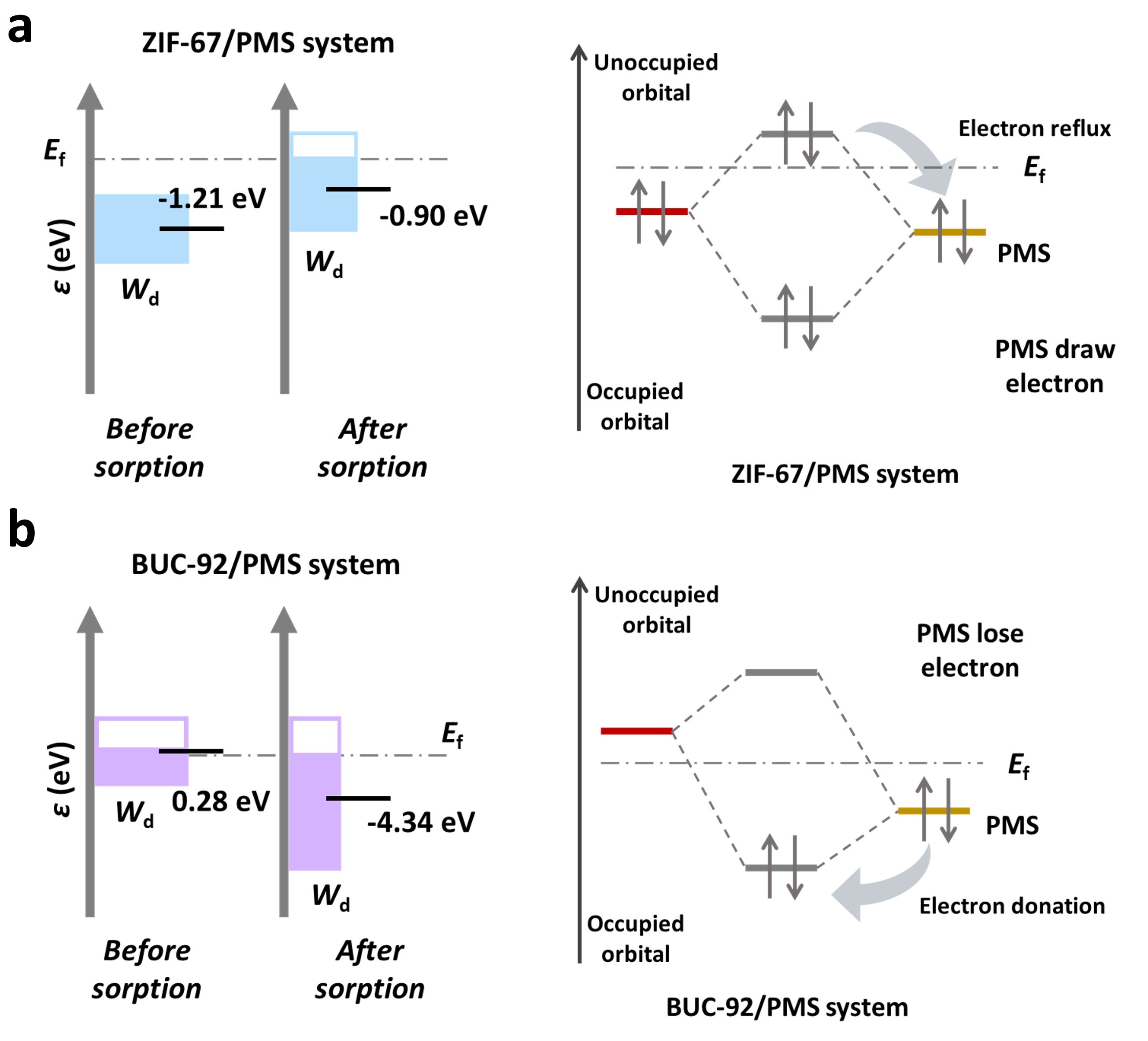


**Figure S28.** The *d*-band center of Co atom from (a) ZIF-67 and (b) BUC-92 before and after PMS adsorption and schematic illustration of bonding and anti-bonding orbitals between Co atom and O atom from PMS.

**Note:**

The *d*-band centers of ZIF-67 and BUC-92 were calculated to -1.21 eV and 0.28 eV, respectively (**Figure S28**). When the *d*-band center lies below the Fermi level, if the formed anti-bonding orbitals between catalyst and PMS could exceed the Fermi level, electrons could be refluxed from anti-bonding orbitals to PMS, further generating radicals. Therefore, ZIF-67 could provide electrons for PMS to generate radicals. If the *d*-band center was below the Fermi level (like BUC-92), electron-occupied O 2*p* orbitals will donate electrons to empty orbitals from Co 3*d*, further generating SO_5_^•−^ and triggering ^1^O_2_ oxidation [3]. As shown in **Figure S29**, the increase in the spin-down empty orbitals indicated that ZIF-67 acted as the electron donor for PMS activation. The decrease in the spin-up empty orbitals indicated that BUC-92 was the electron acceptor.


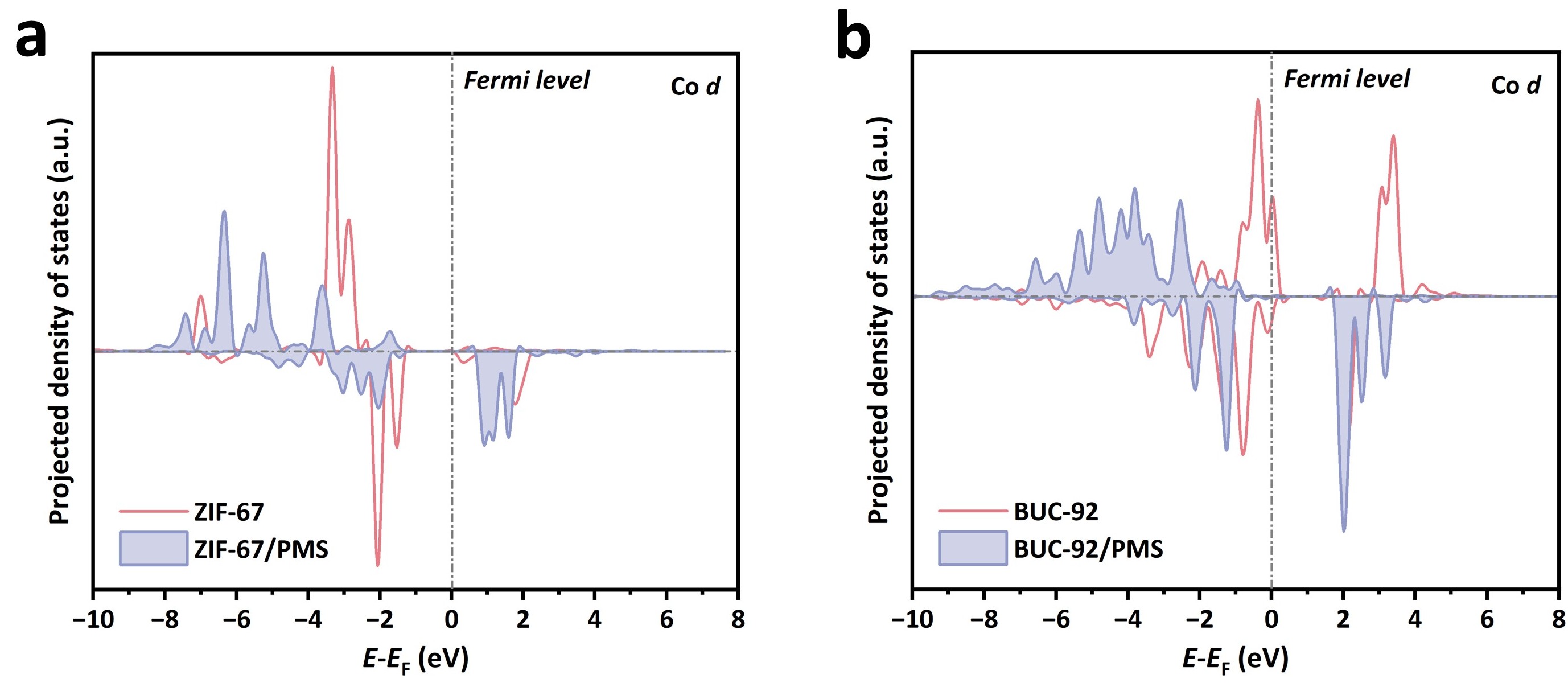


**Figure S29.** PDOS of Co *d* orbitals in (a) ZIF-67 and (b) BUC-92 before and after PMS adsorption.


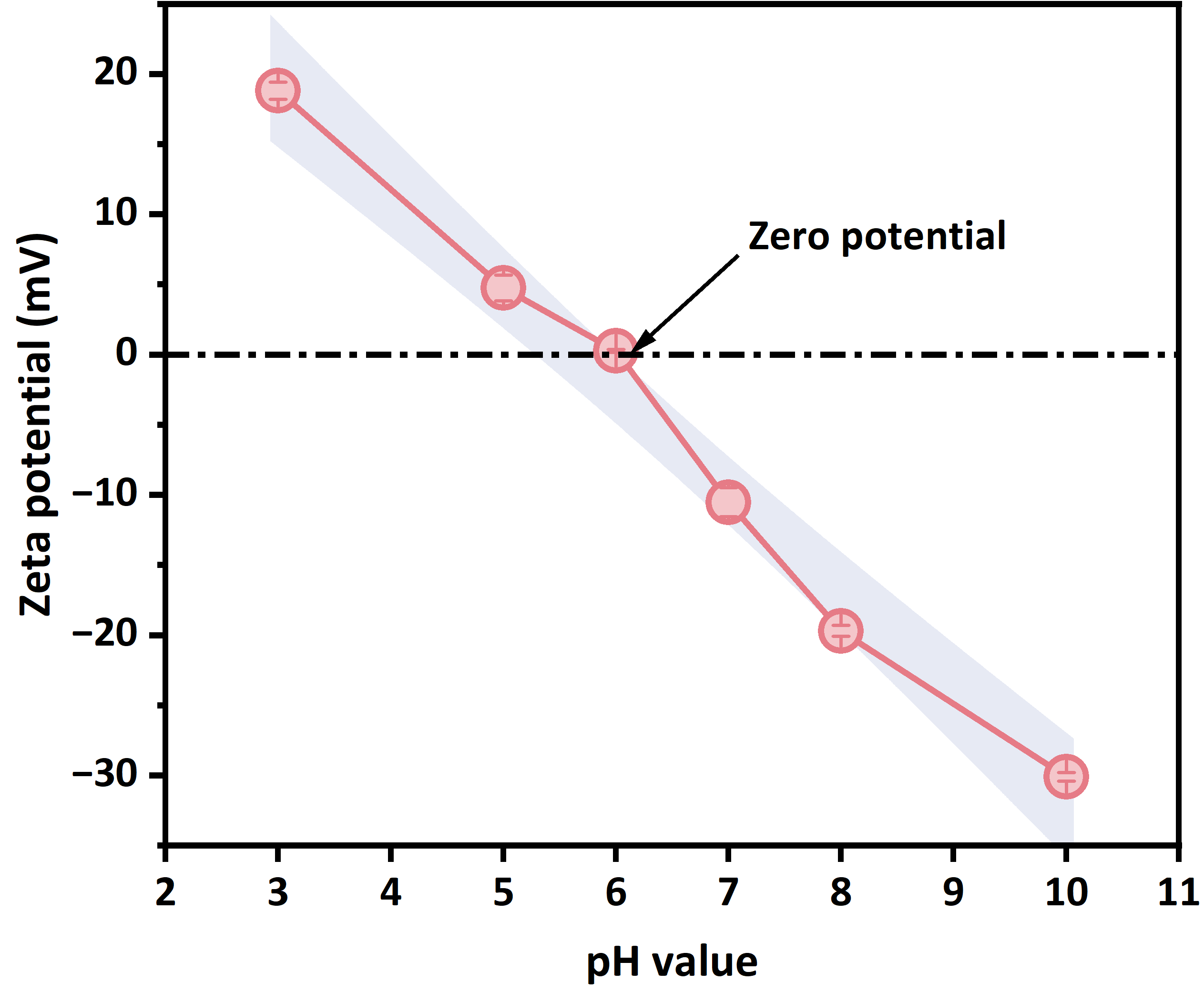


**Figure S30.** Zeta potentials of PKU-24 in different pH aqueous solutions. The error bars in the figures represented the standard deviations from triplicate tests.


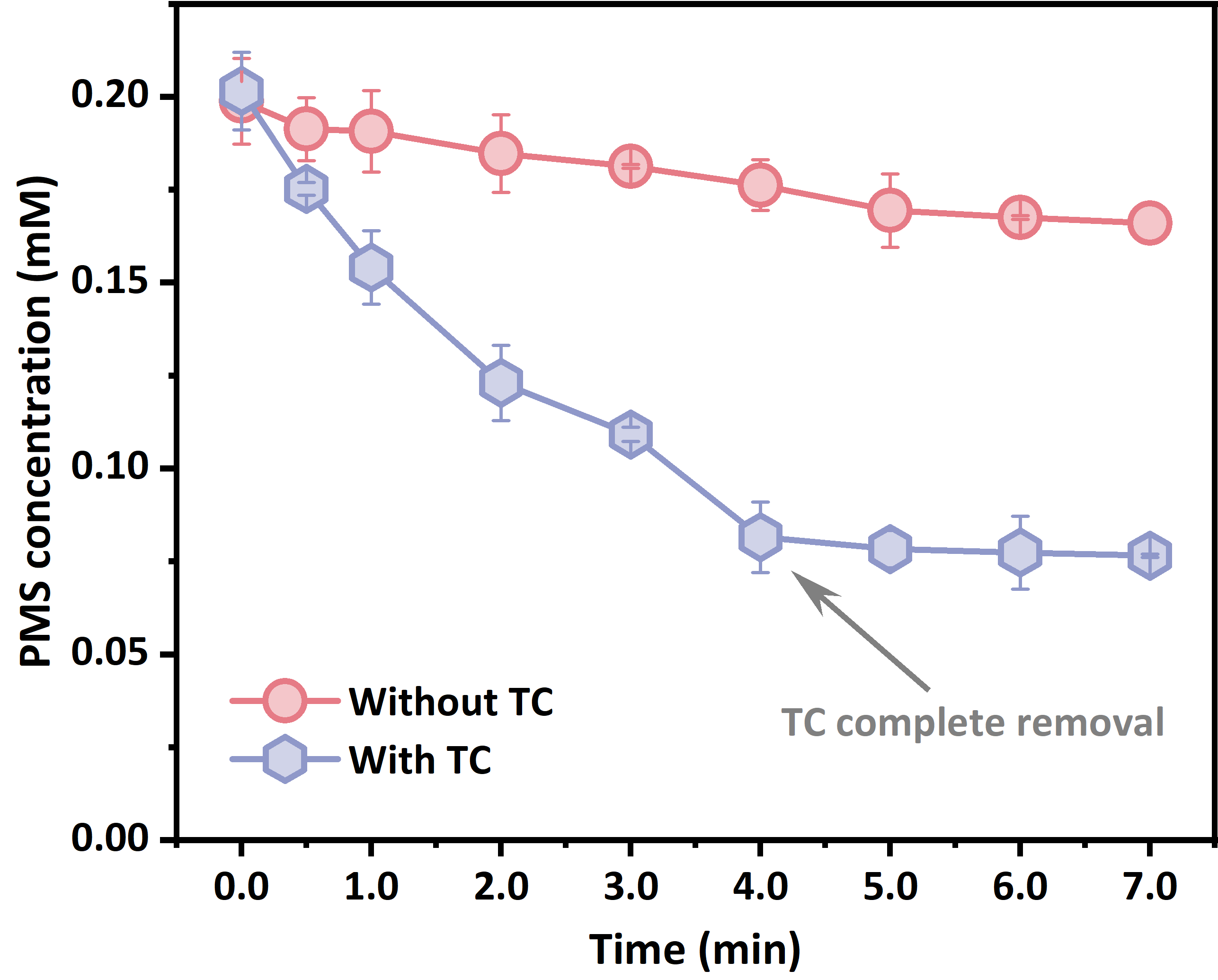


**Figure S31.** The change of PMS concentrations during Fenton-like reaction with or without TC. The error bars in the figures represented the standard deviations from triplicate tests.

**Experimental conditions:** [Catalyst] = 0.2 g L^–1^, [TC] = 10.0 mg L^–1^, [PMS] = 0.2 mM.


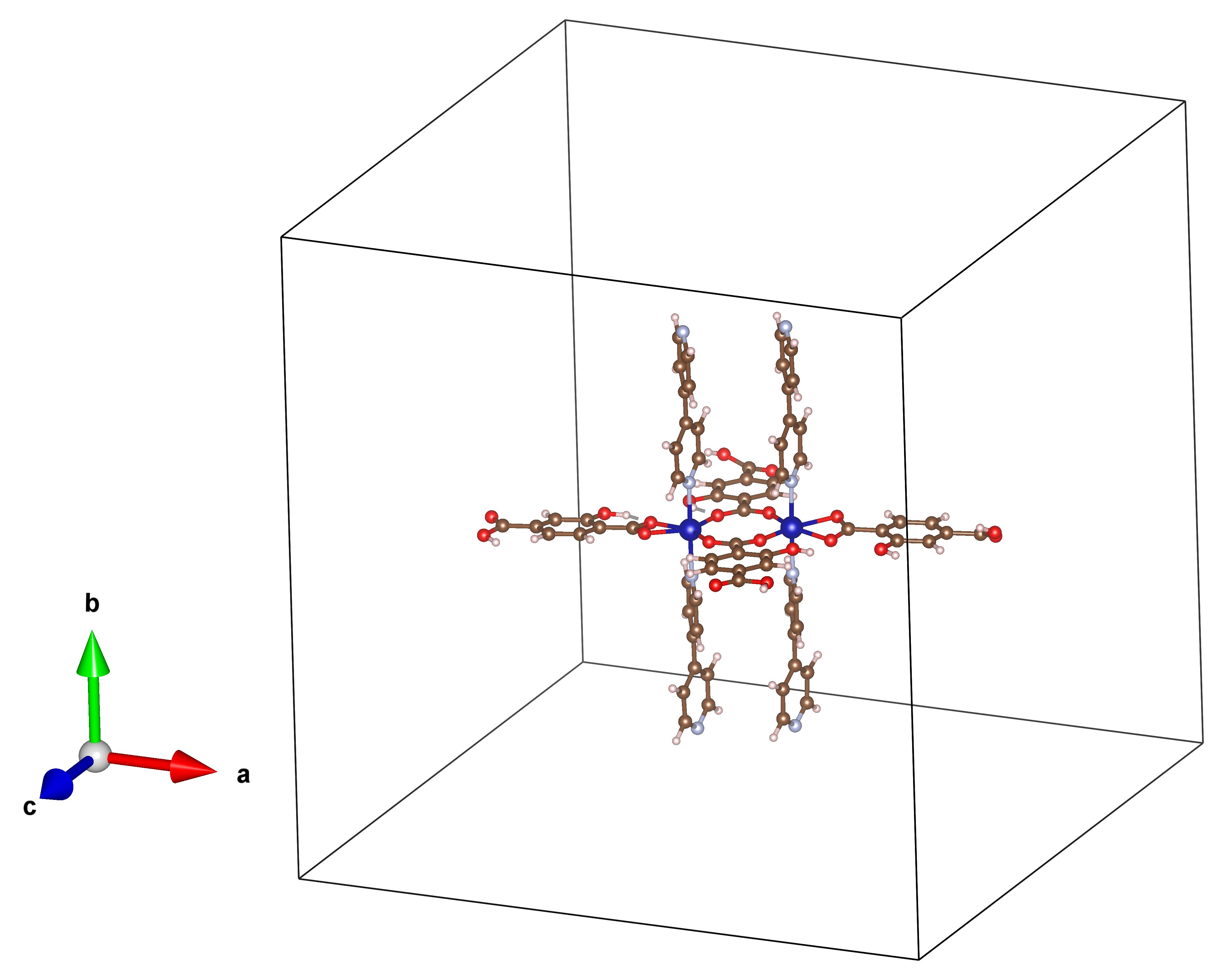


**Figure S32.** The PKU-24 calculation model after structure optimization.


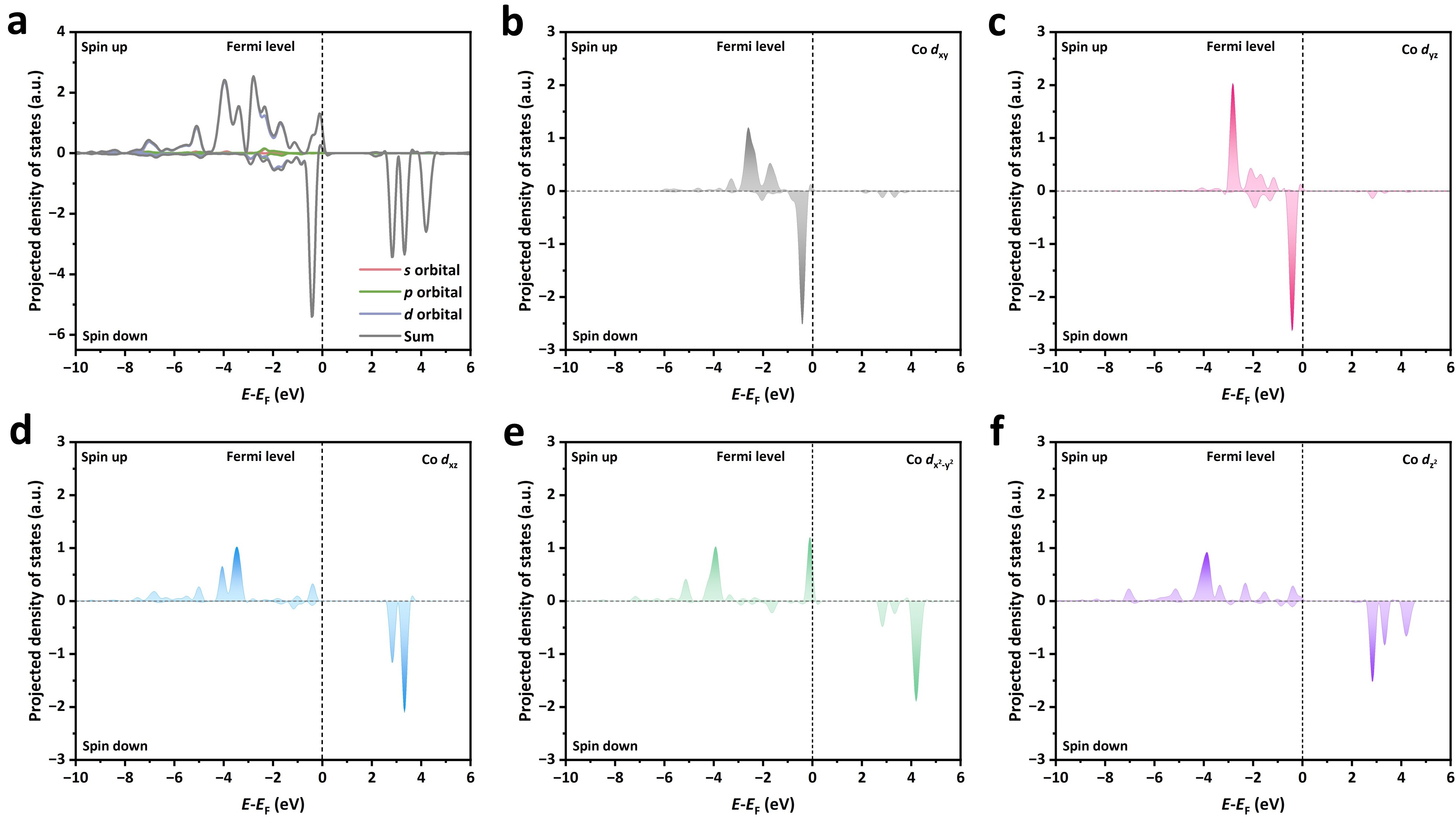


**Figure S33.** The PDOS of (a) Co atom in PKU-24 and (b-f) the corresponding projected orbitals.


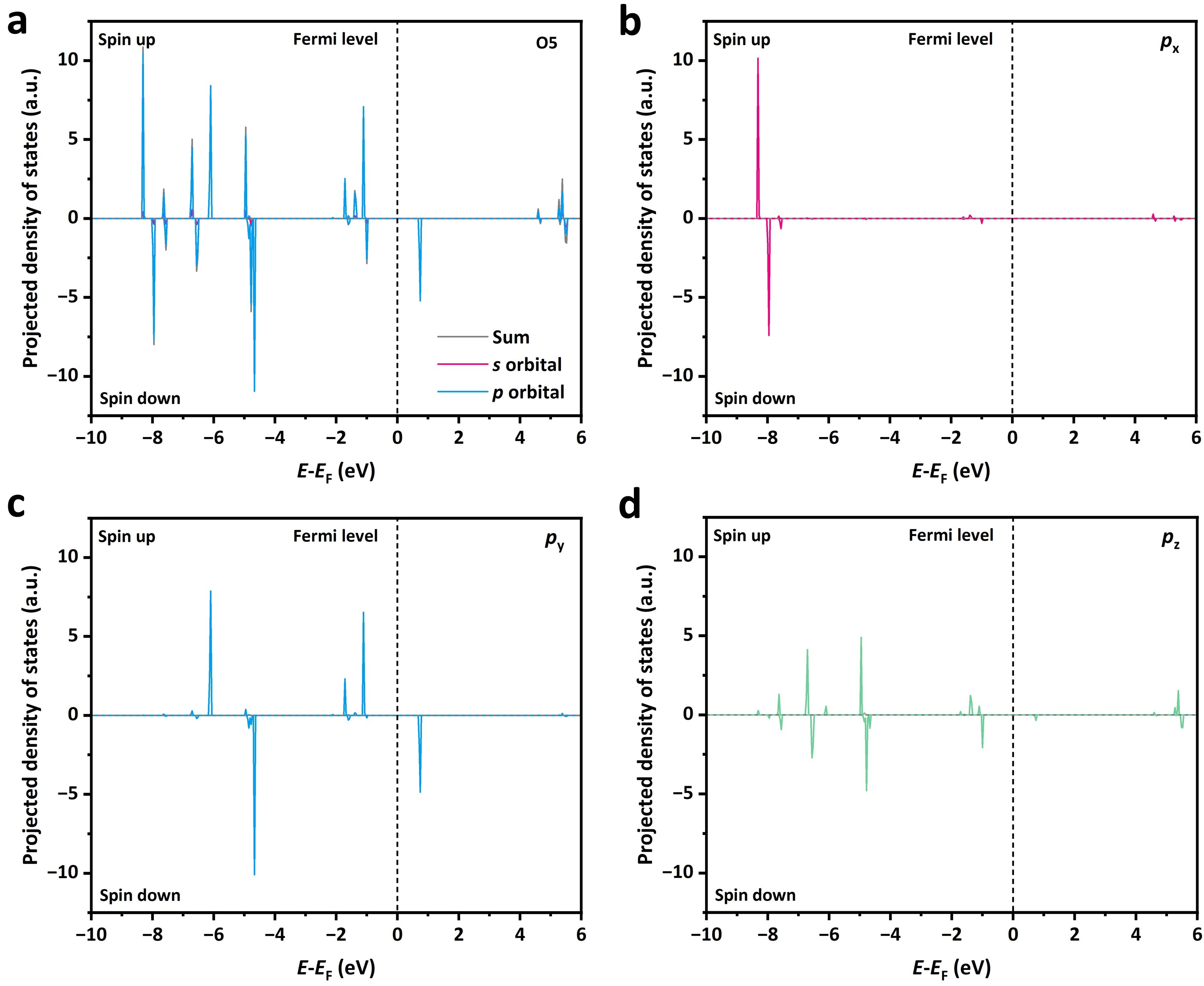


**Figure S34.** The PDOS of (a) O5 atom in PMS and (b-d) the corresponding projected orbitals.


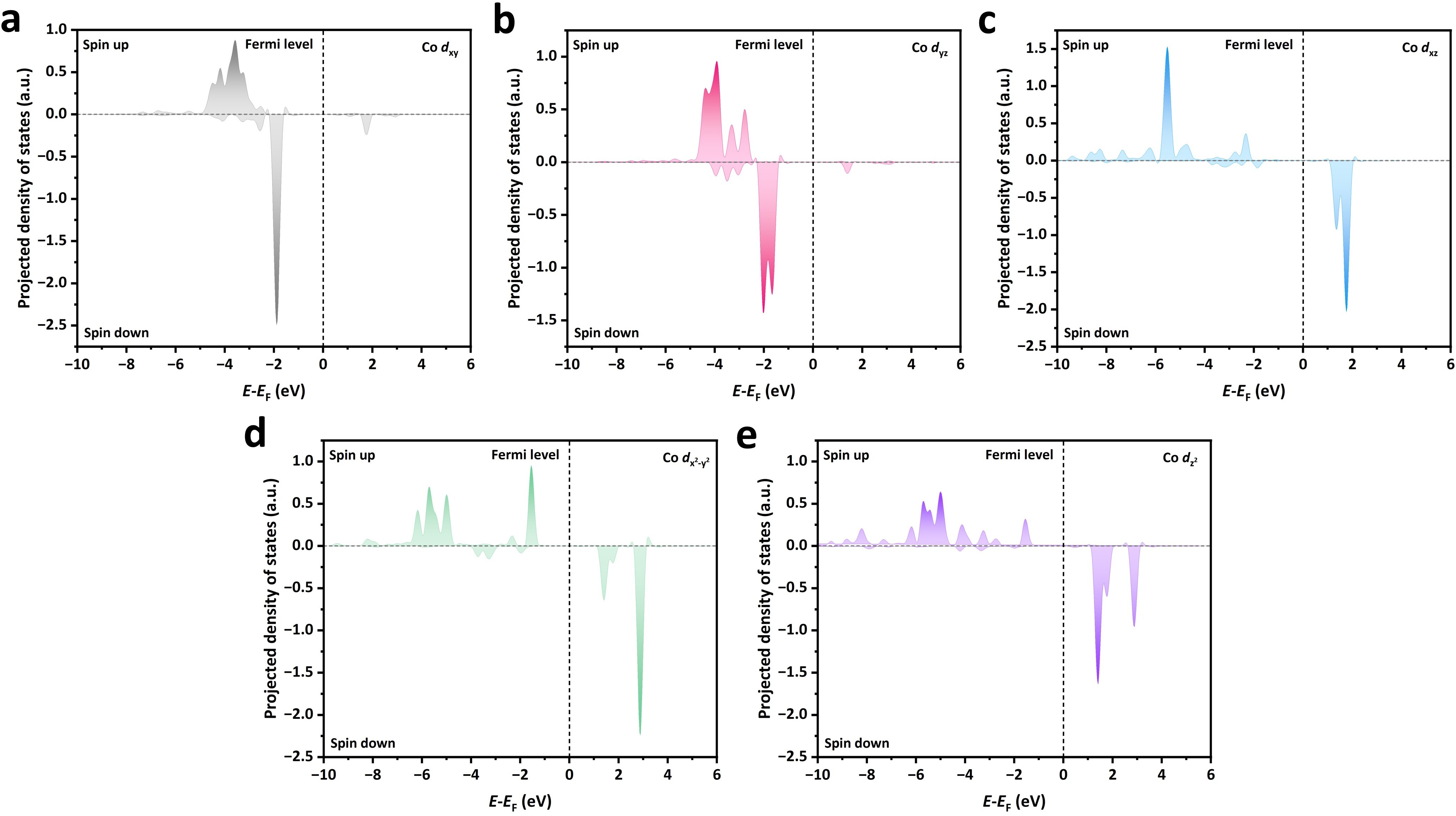


**Figure S35.** (a-e) The corresponding projected orbitals of Co atom in PKU-24 after PMS adsorption.


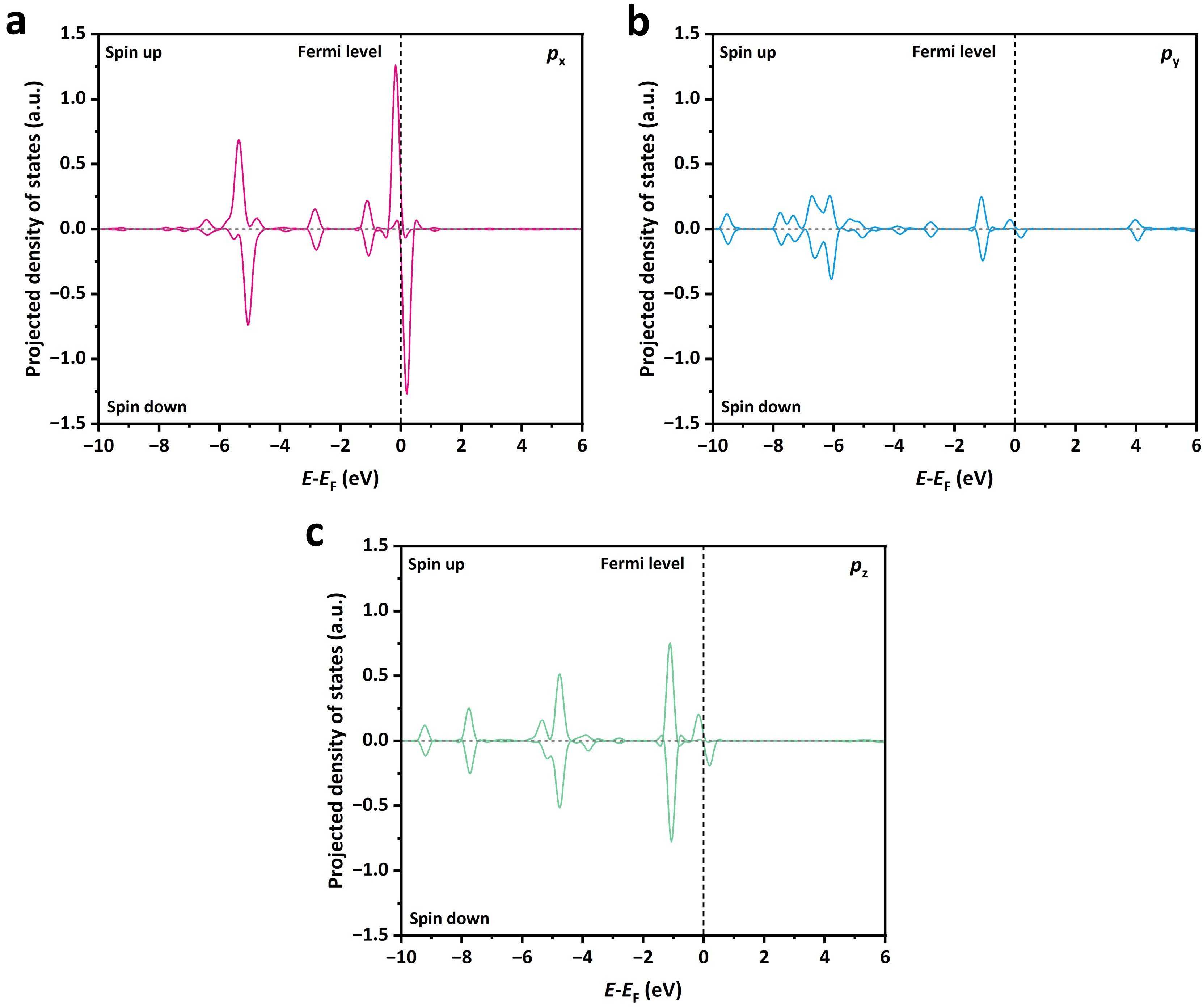


**Figure S36.** (a-c) The corresponding projected orbitals of O5 atom in the adsorbed PMS.


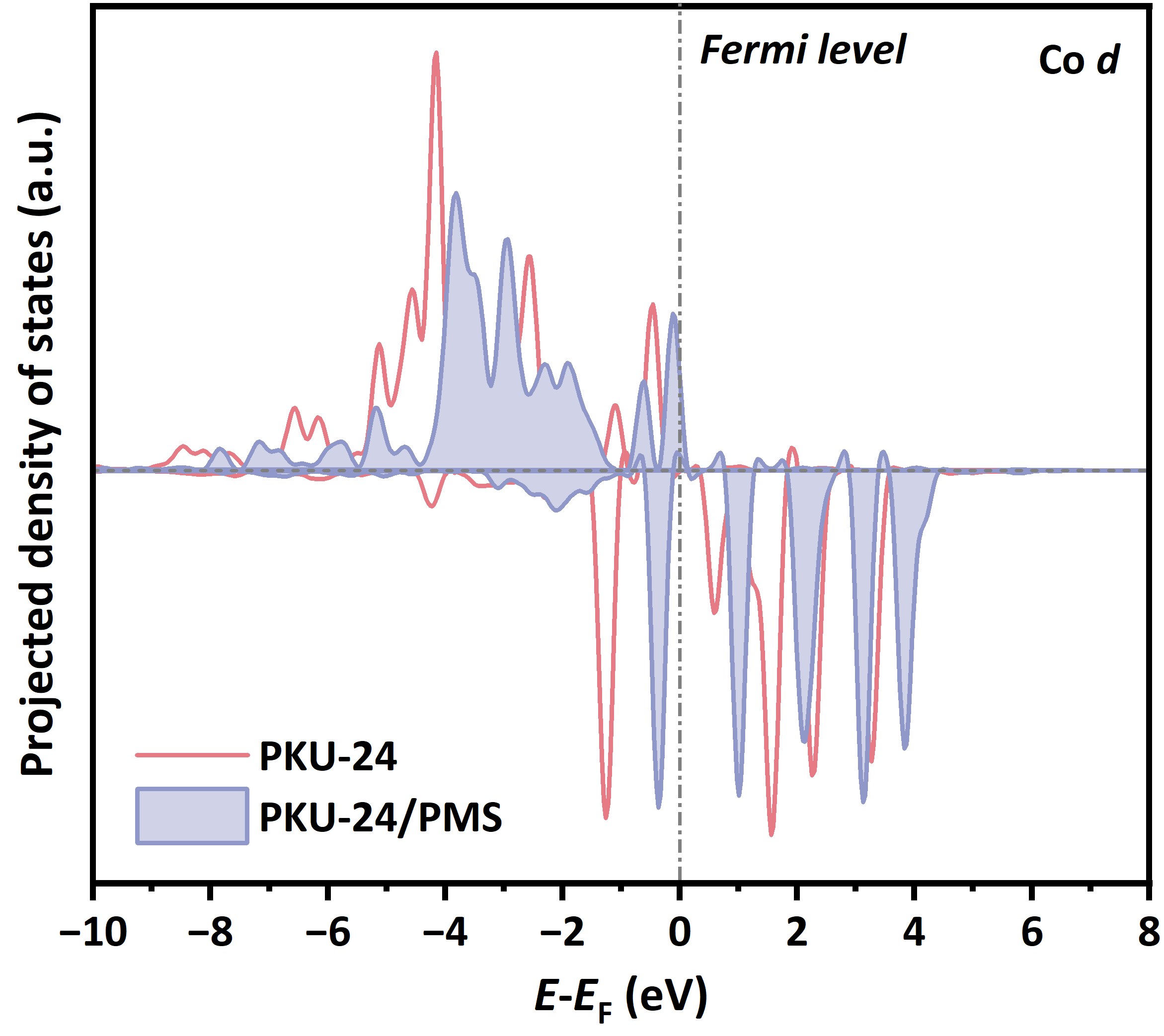


**Figure S37.** PDOS of Co *d* orbital in PKU-24 before and after PMS adsorption.


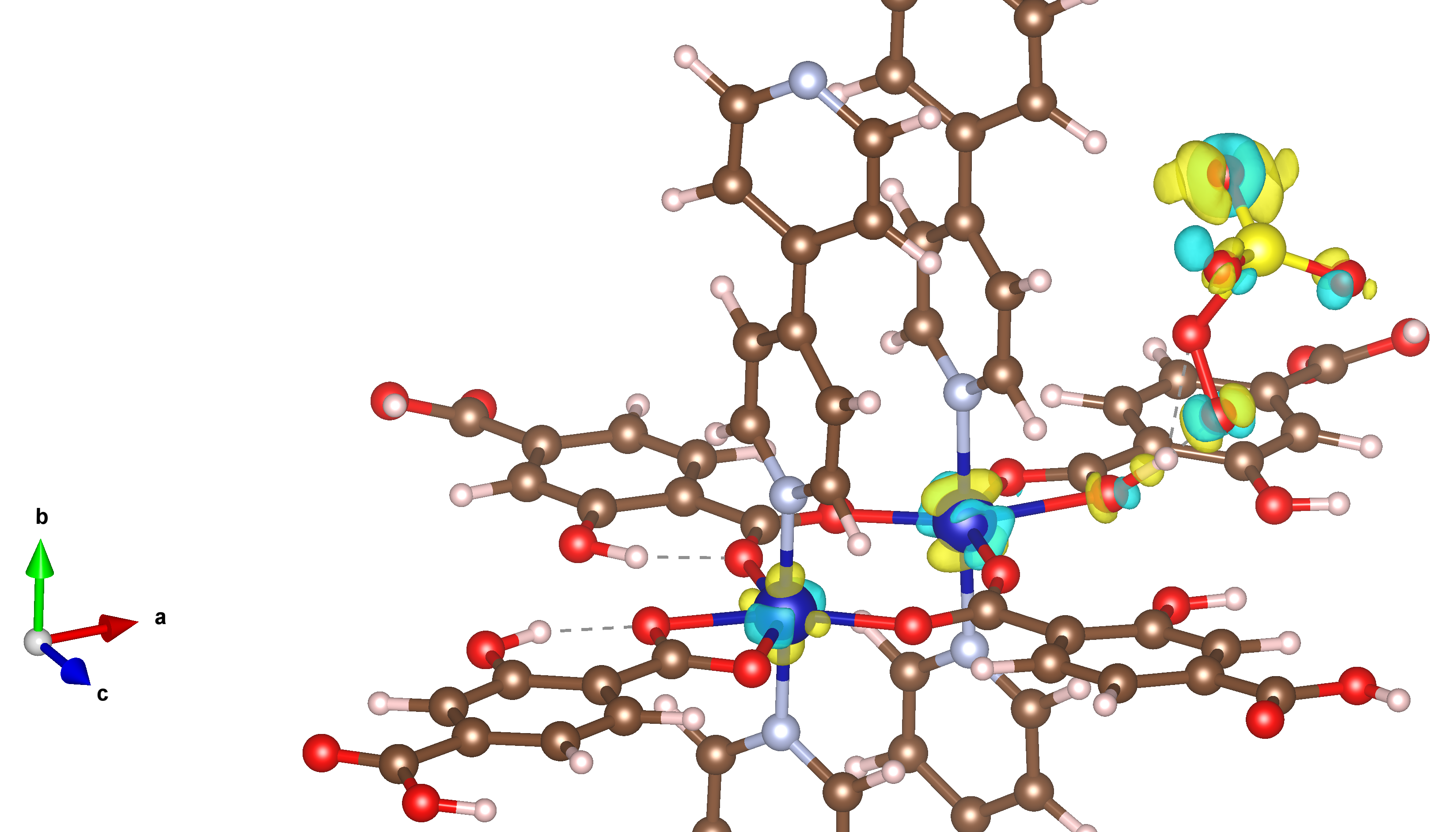


**Figure S38.** The electron density difference of PKU-24-PMS model (yellow and blue represented the accumulation and dissipation of electron clouds, respectively).


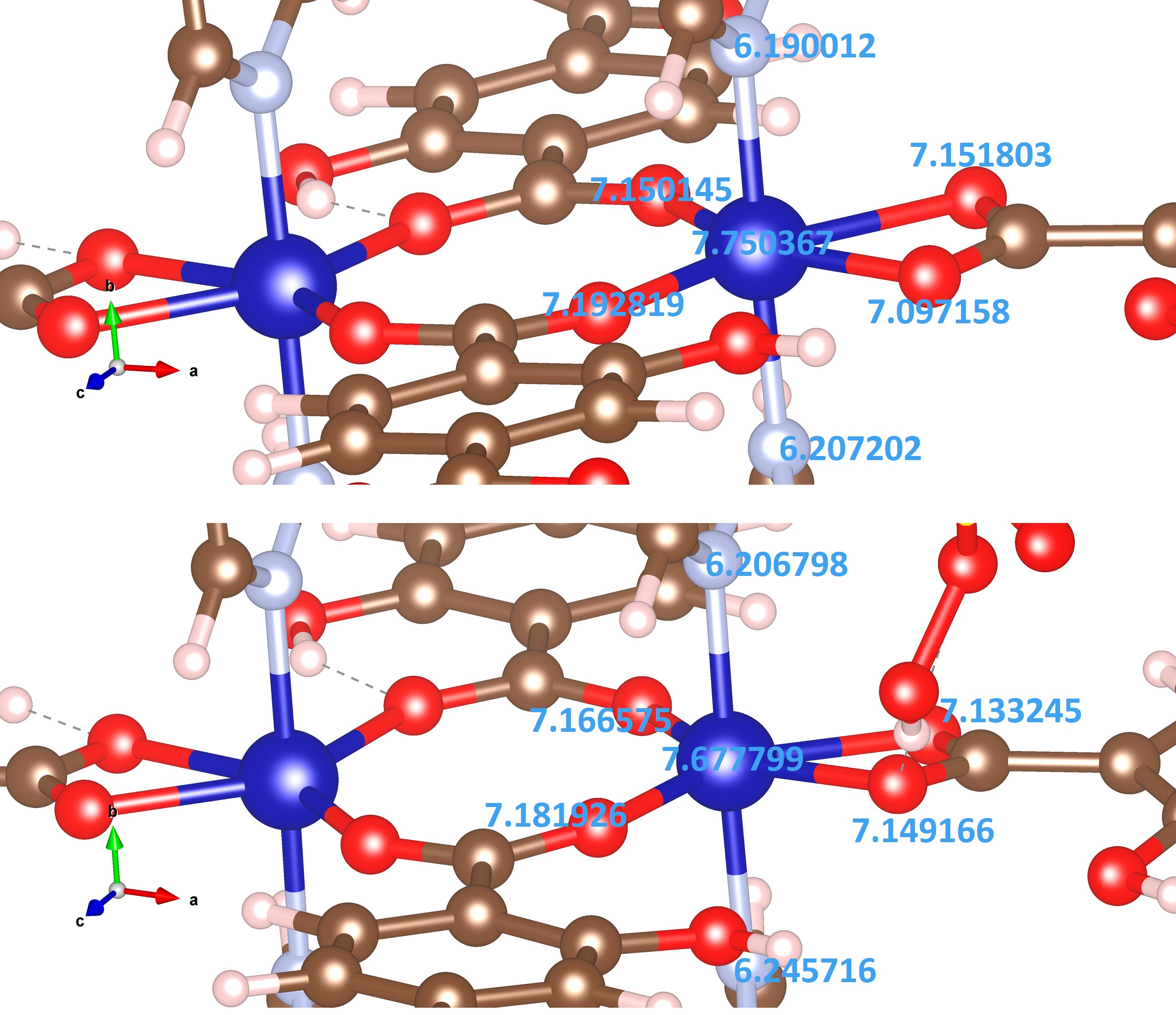


**Figure S39.** The change of the Bader charge of [Co-N_2_O_4_] center before and after PMS adsorption.


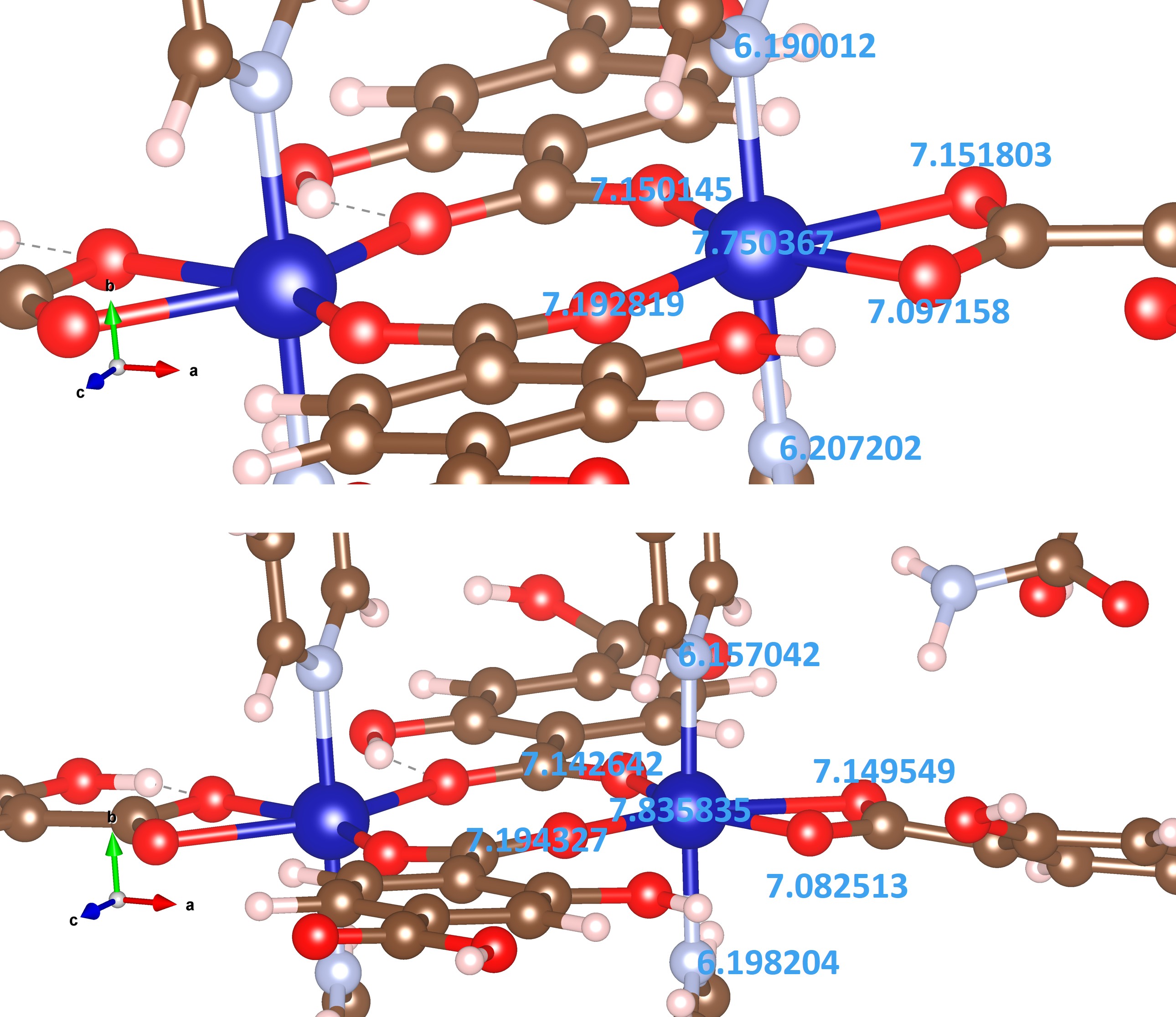


**Figure S40.** The change of the Bader charge of [Co-N_2_O_4_] center before and after TC adsorption.


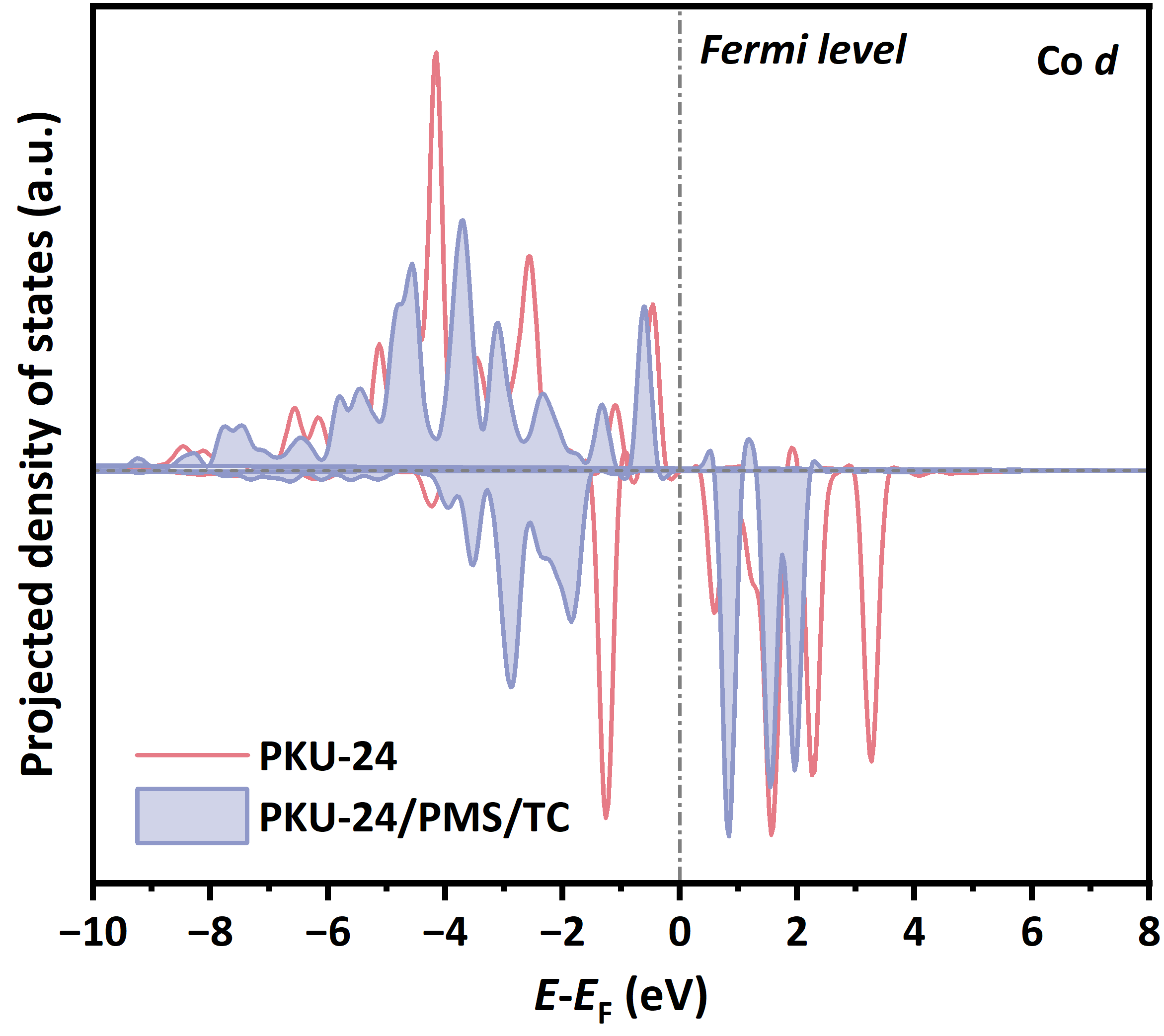


**Figure S41.** PDOS of Co *d* orbital in PKU-24 before and after PMS/TC co-adsorption.


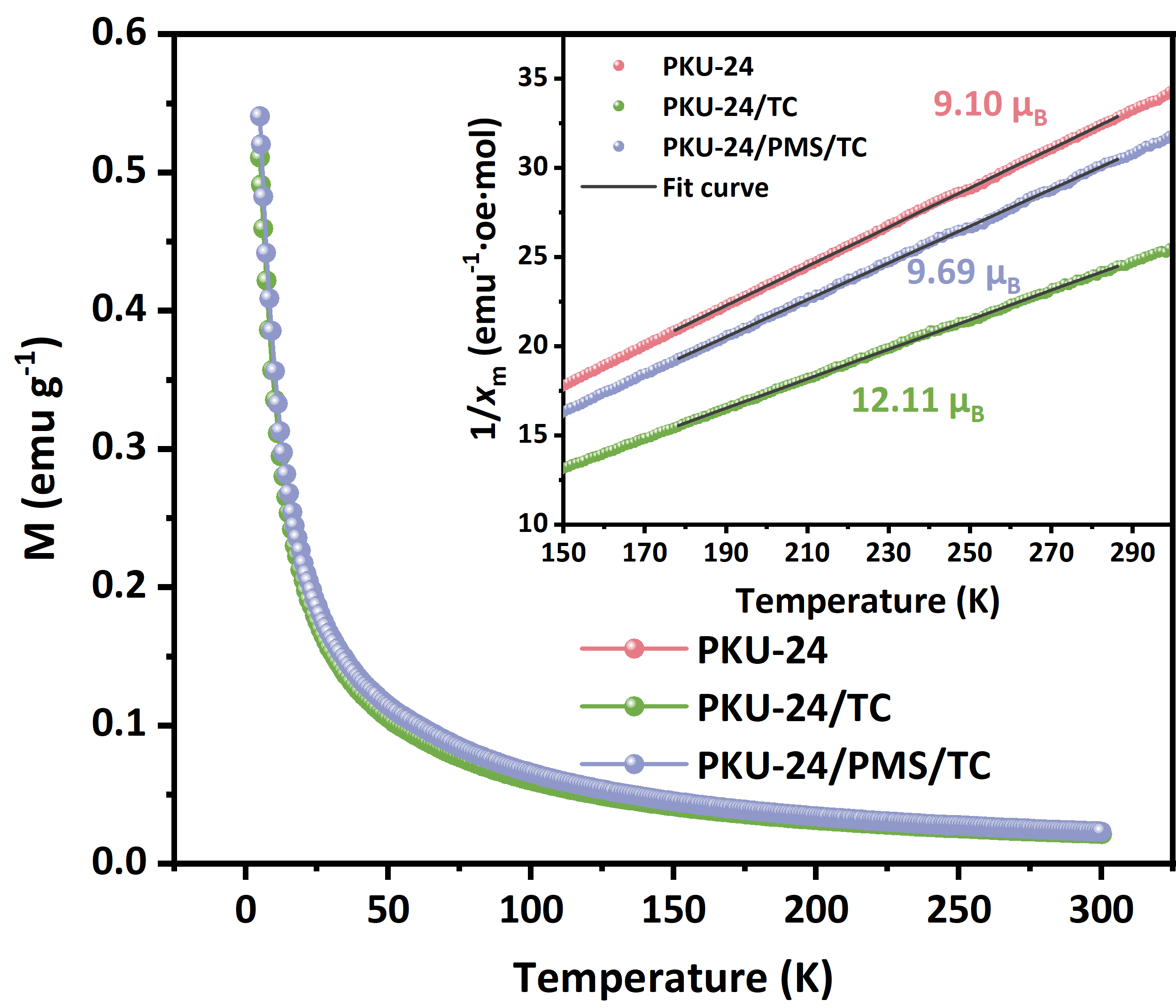


**Figure S42.** The M-T curves of PKU-24 before and after reaction.


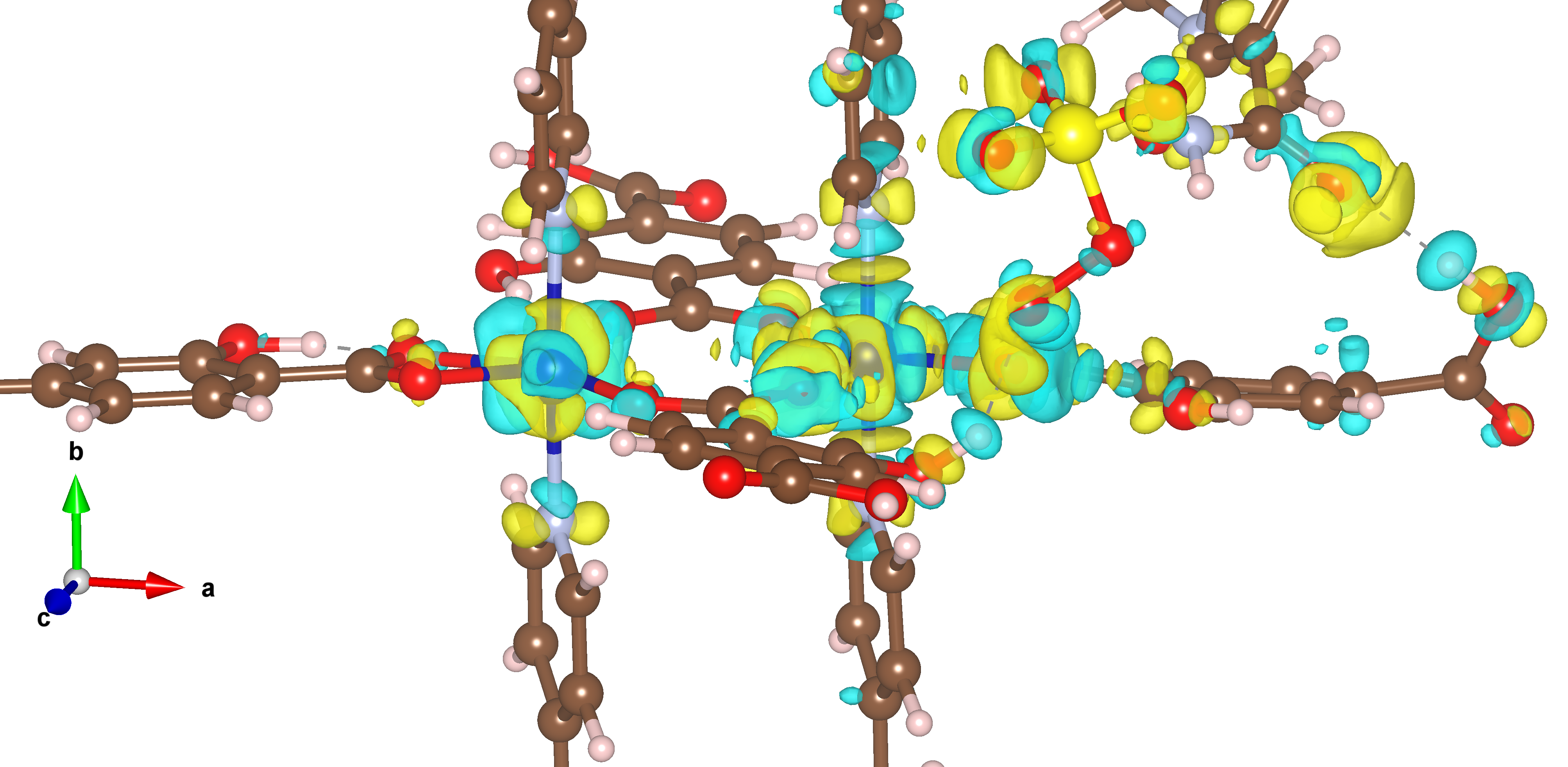


**Figure S43.** The electron density difference of PKU-24-PMS-TC model (yellow and blue represented the accumulation and dissipation of electron clouds, respectively).


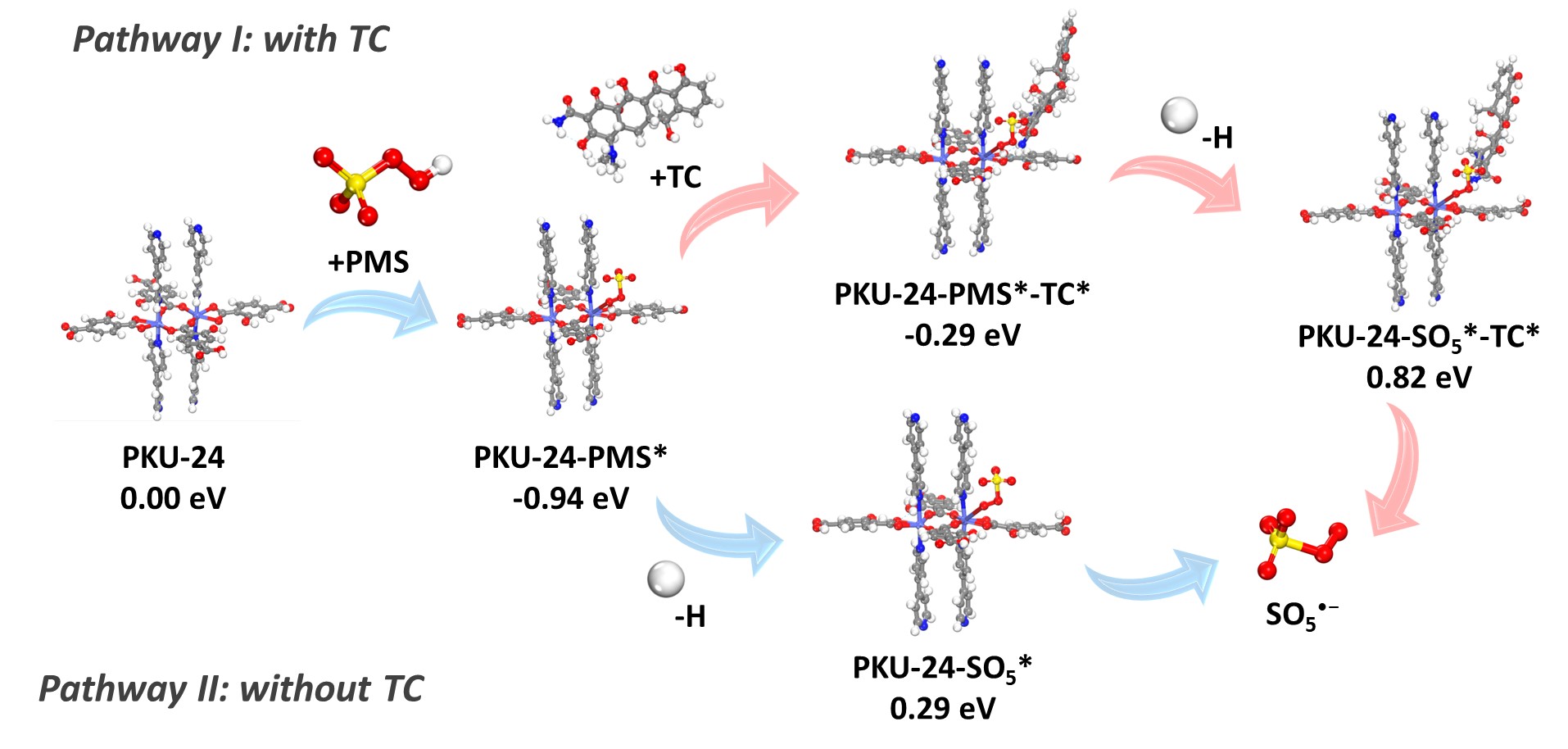


**Figure S44.** The key intermediates of reaction pathways and Gibbs free energy diagrams for PMS activation.


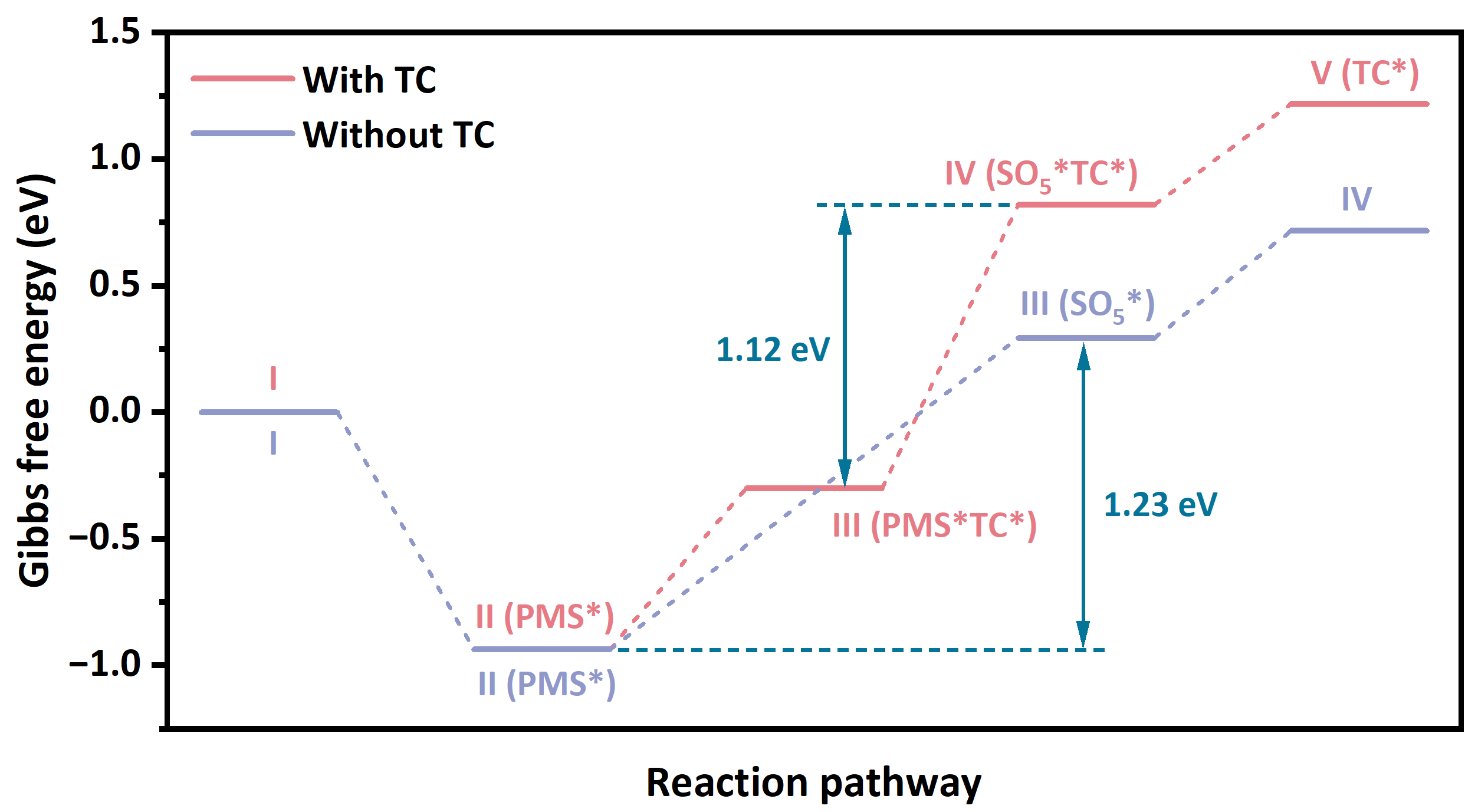


**Figure S45.** Gibbs free energy diagrams for PMS decomposition into two key reaction intermediates.


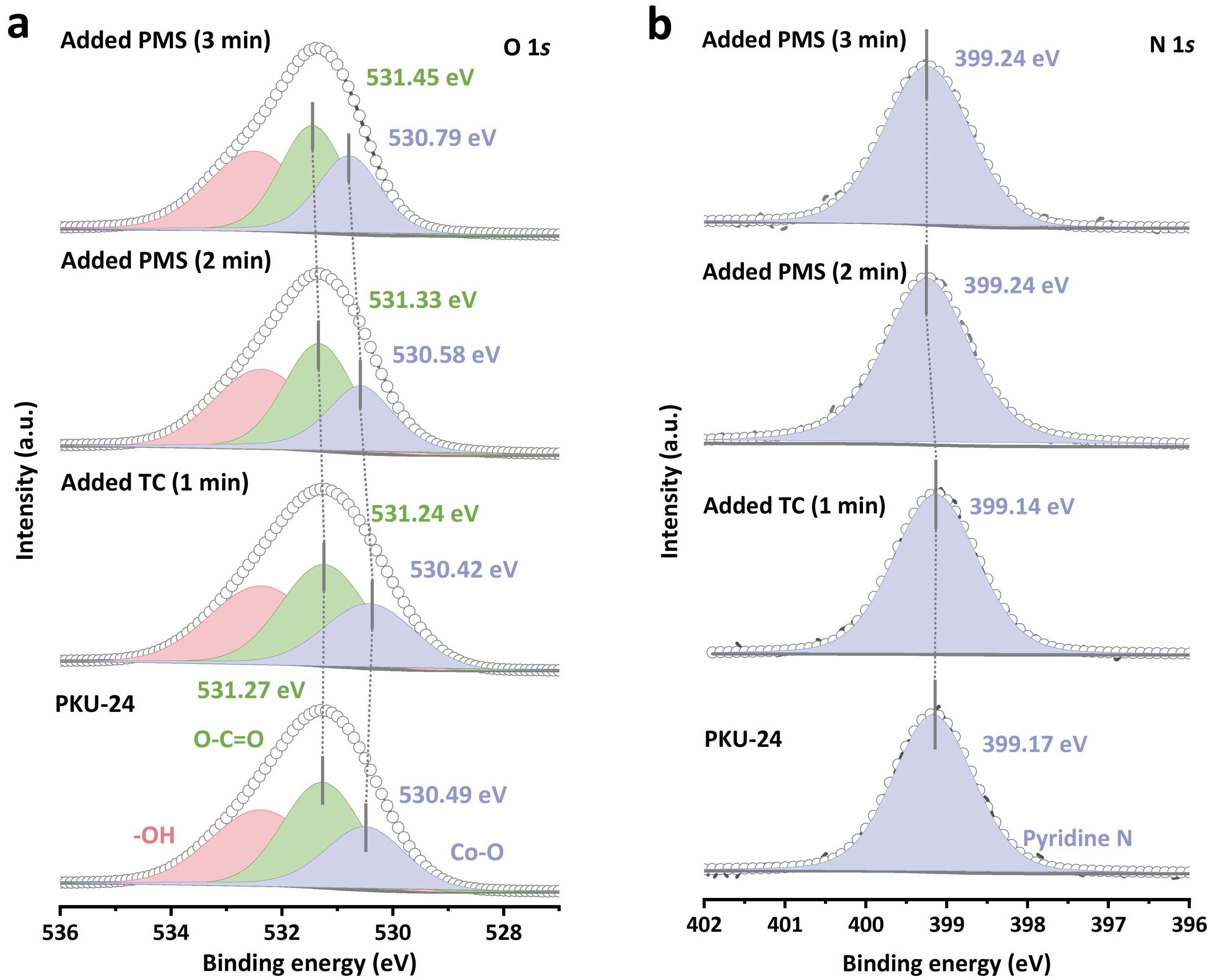


**Figure S46.** In-situ XPS (a) O 1*s* and (b) N 1*s* spectra of PKU-24/PMS/TC.


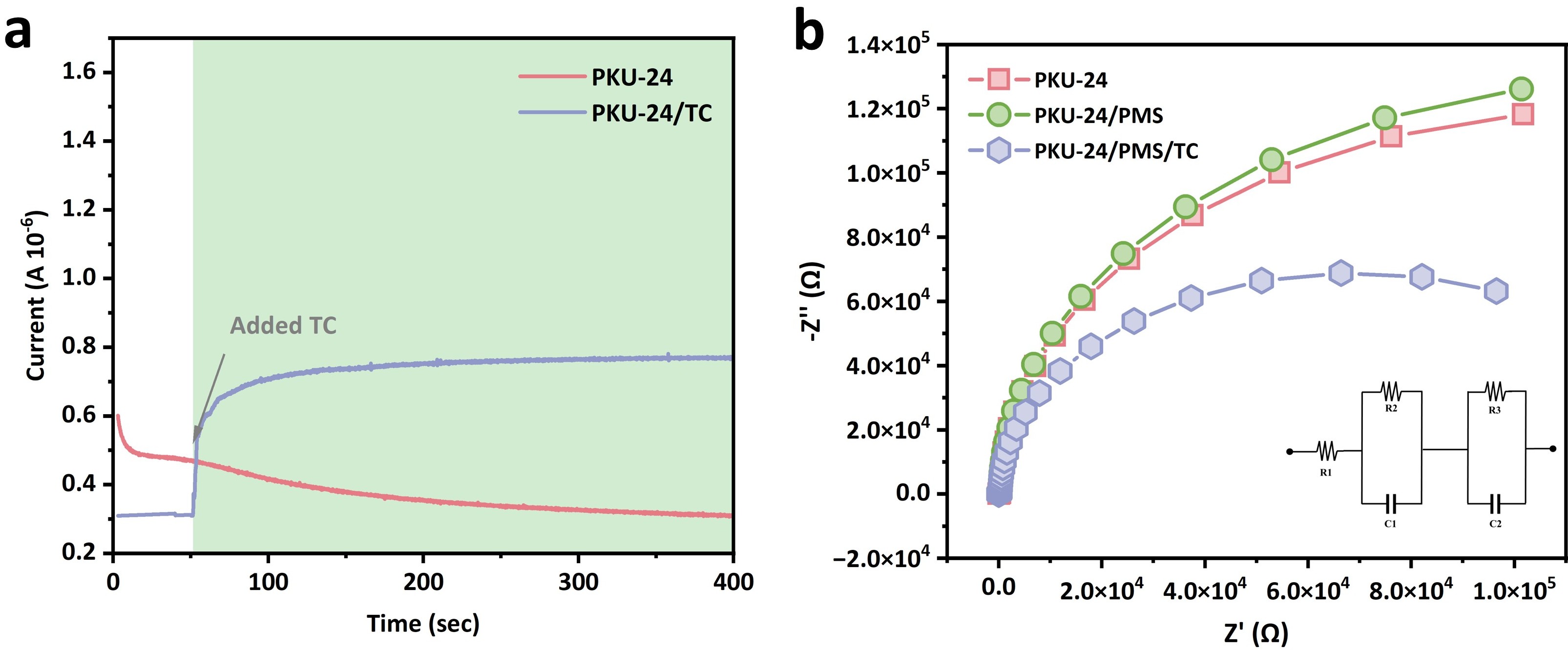


**Figure S47.** (a) Amperometric *i*-*t* curves and (b) EIS curves in different systems using PKU-24 as working electrode.


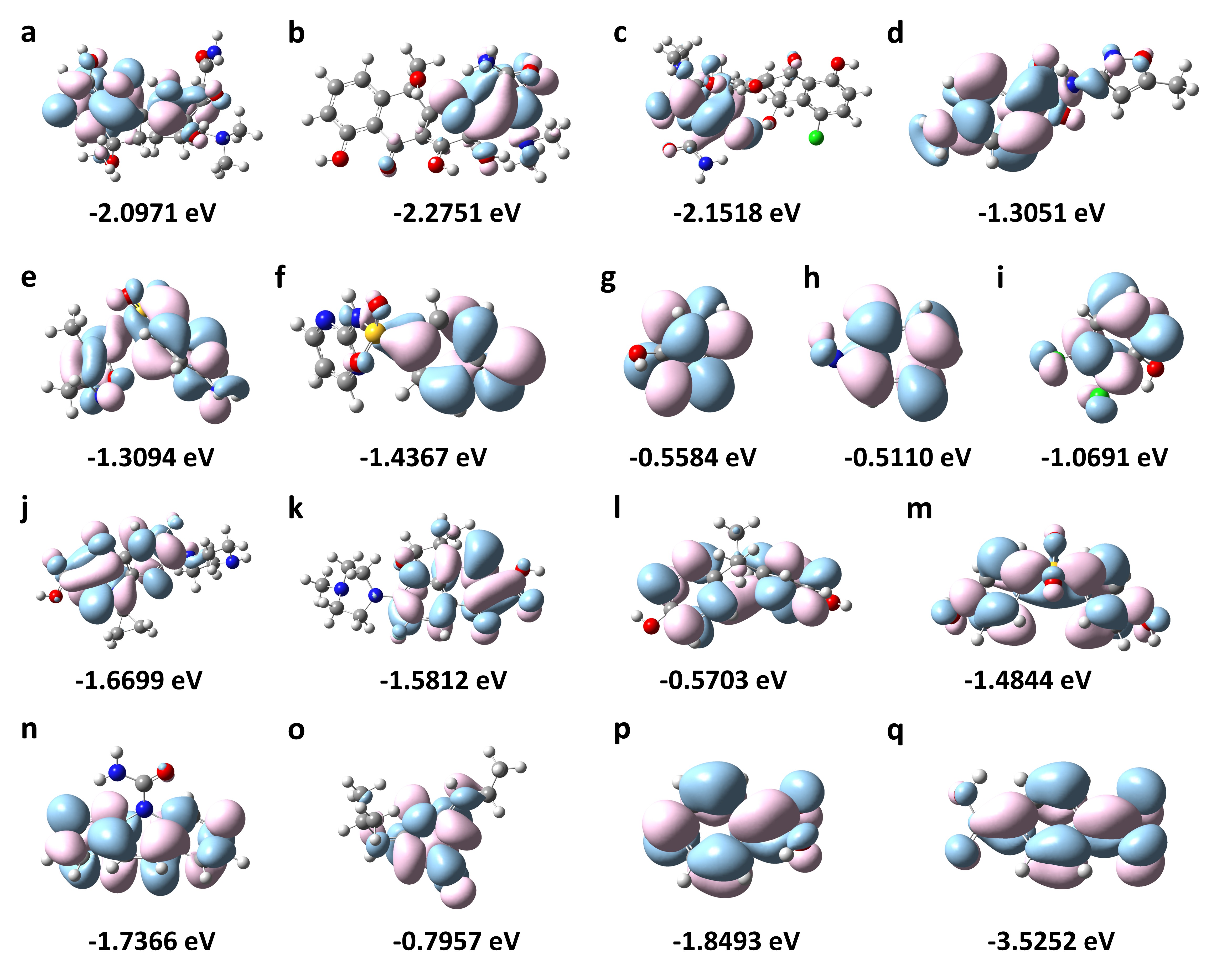


**Figure S48.** The LUMO of (a) TC, (b) CTC, (c) OTC, (d) SMX, (e) SIX, (f) SDZ, (g) PN, (h) AN, (i) DCP, (j) CIP, (k) OFX, (l) BPA, (m) BPS, (n) CBZ, (o) ATZ, (p) BA and (q) NBA.


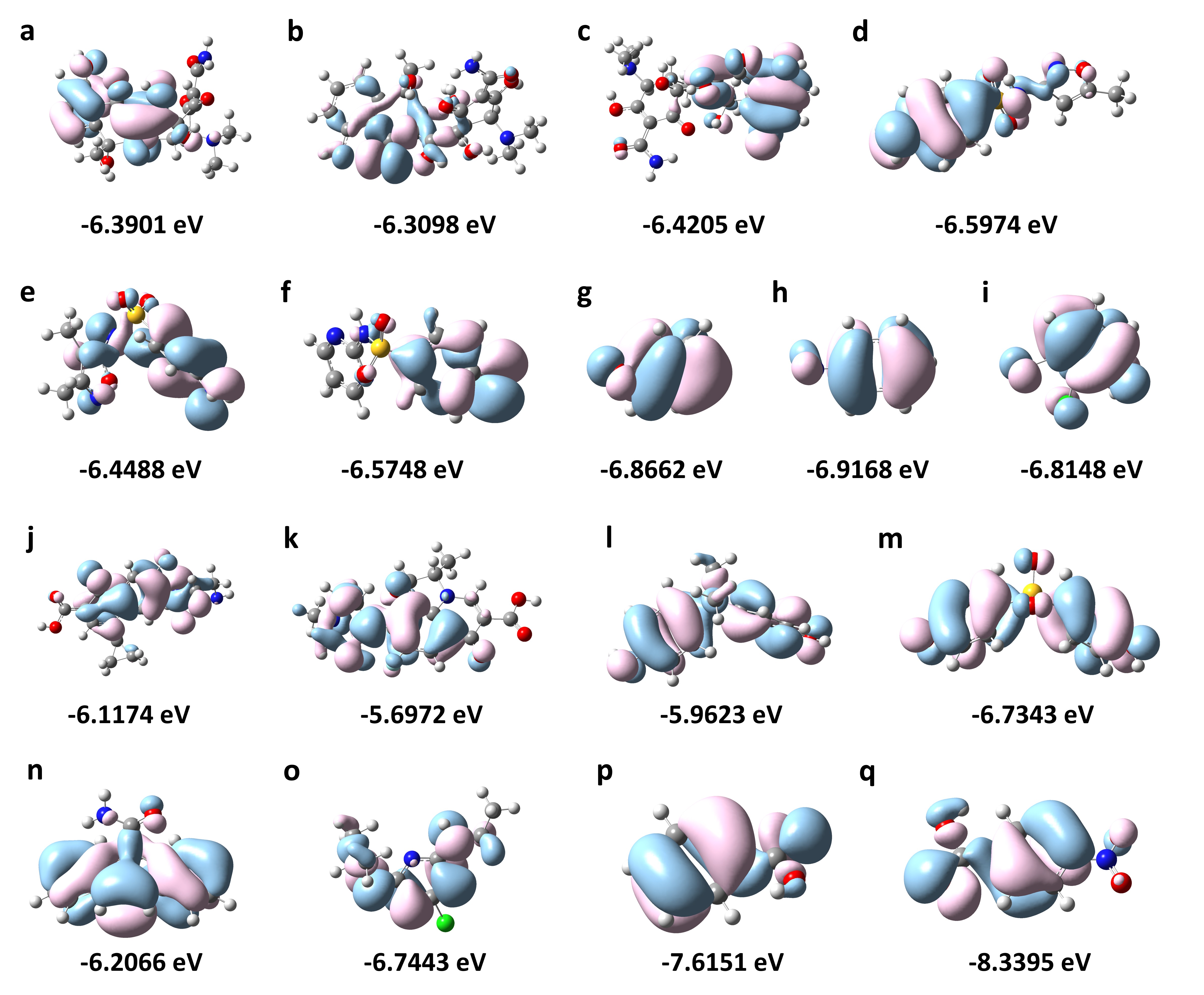


**Figure S49.** The HOMO of (a) TC, (b) CTC, (c) OTC, (d) SMX, (e) SIX, (f) SDZ, (g) PN, (h) AN, (i) DCP, (j) CIP, (k) OFX, (l) BPA, (m) BPS, (n) CBZ, (o) ATZ, (p) BA and (q) NBA.


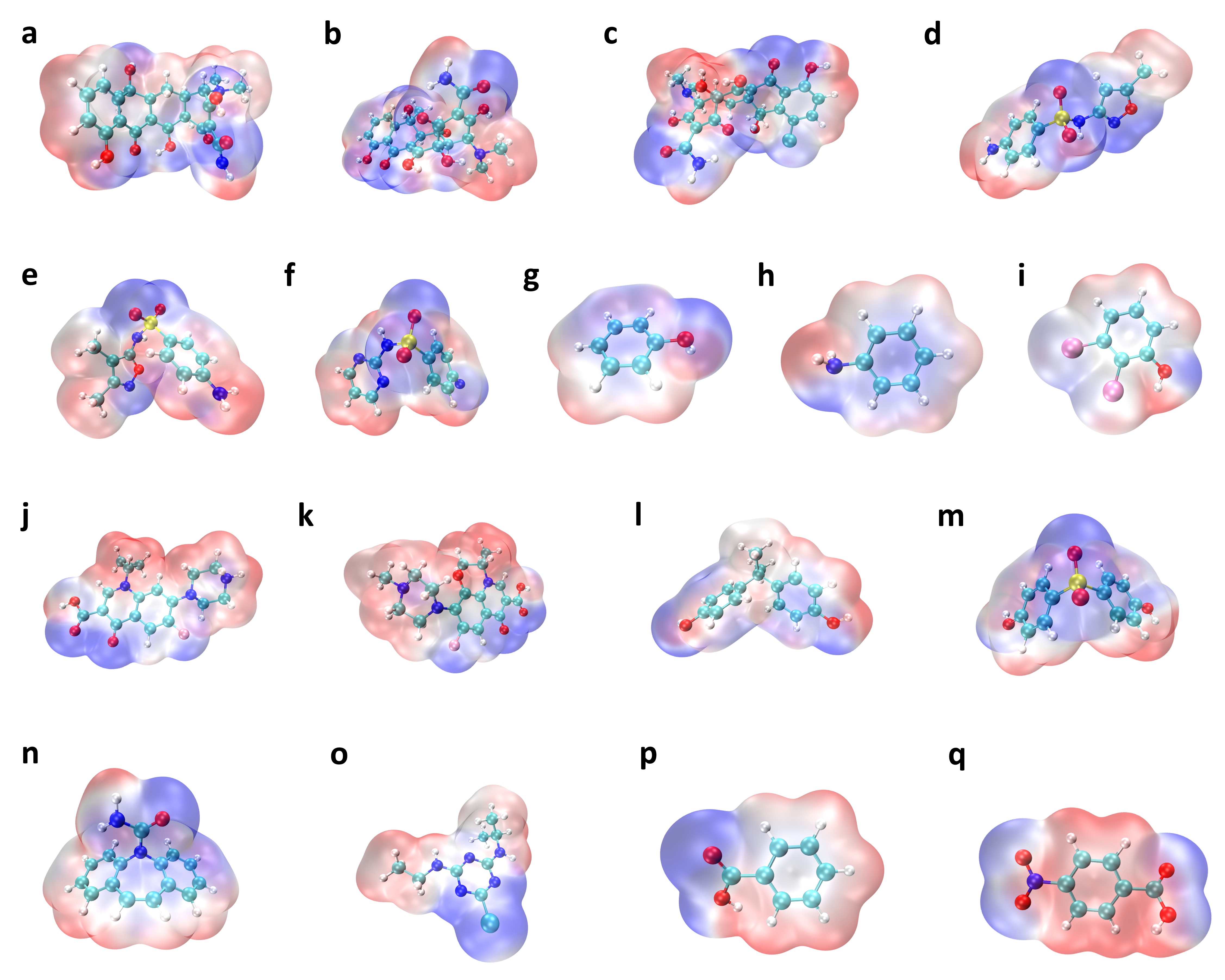


**Figure S50.** The ESP of (a) TC, (b) CTC, (c) OTC, (d) SMX, (e) SIX, (f) SDZ, (g) PN, (h) AN, (i) DCP, (j) CIP, (k) OFX, (l) BPA, (m) BPS, (n) CBZ, (o) ATZ, (p) BA and (q) NBA.


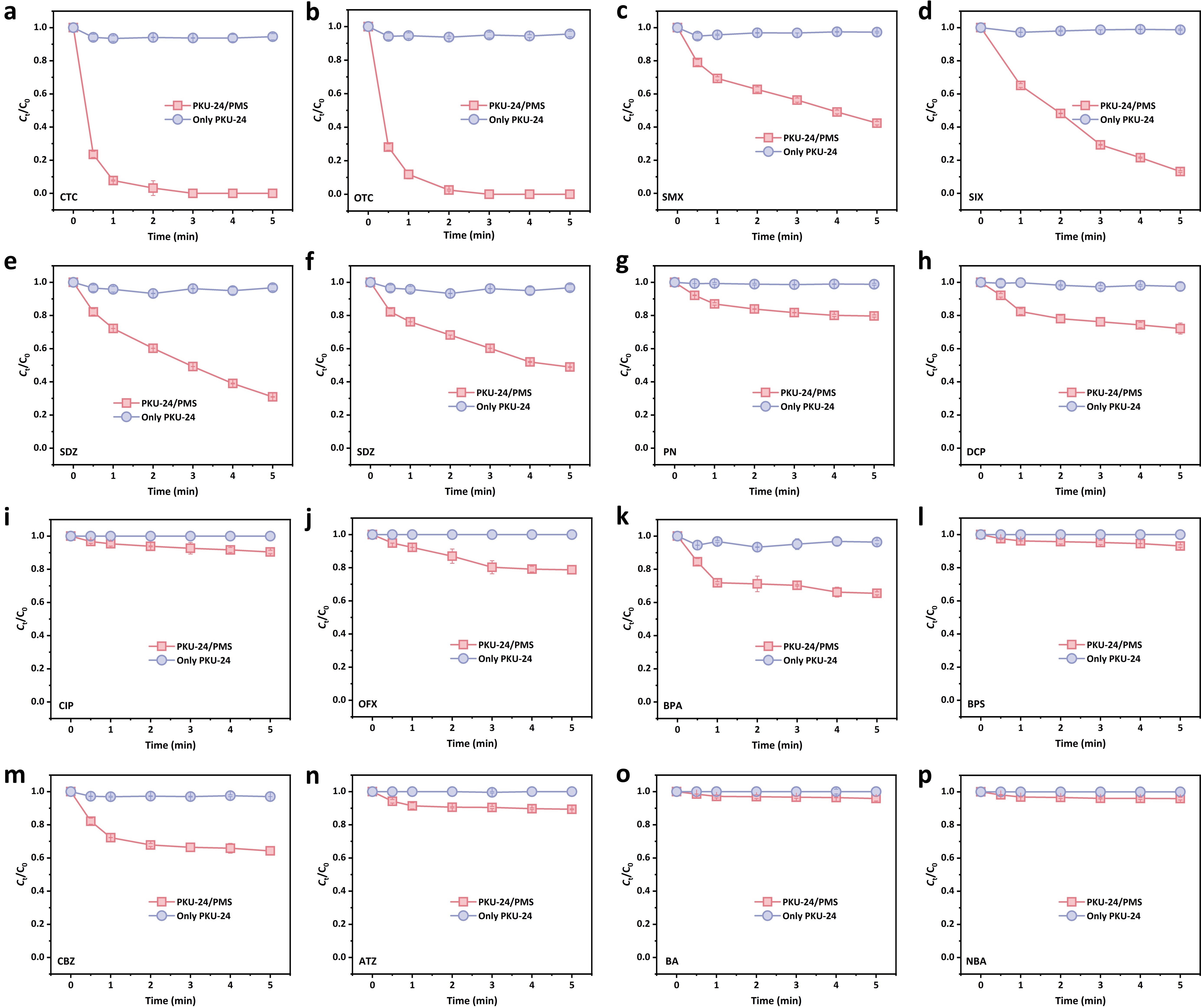


**Figure S51.** The catalytic degradation performances toward (a) CTC, (b) OTC, (c) SMX, (d) SIX, (e) SDZ, (f) PN, (g) AN, (h) DCP, (i) CIP, (j) OFX, (k) BPA, (l) BPS, (m) CBZ, (n) ATZ, (o) BA and (p) NBA. The error bars in the figures represented the standard deviations from triplicate tests.

**Experimental conditions:** [Catalyst] = 0.2 g L^–1^, [Pollutant] = 10.0 mg L^–1^, [PMS] = 0.2 mM.


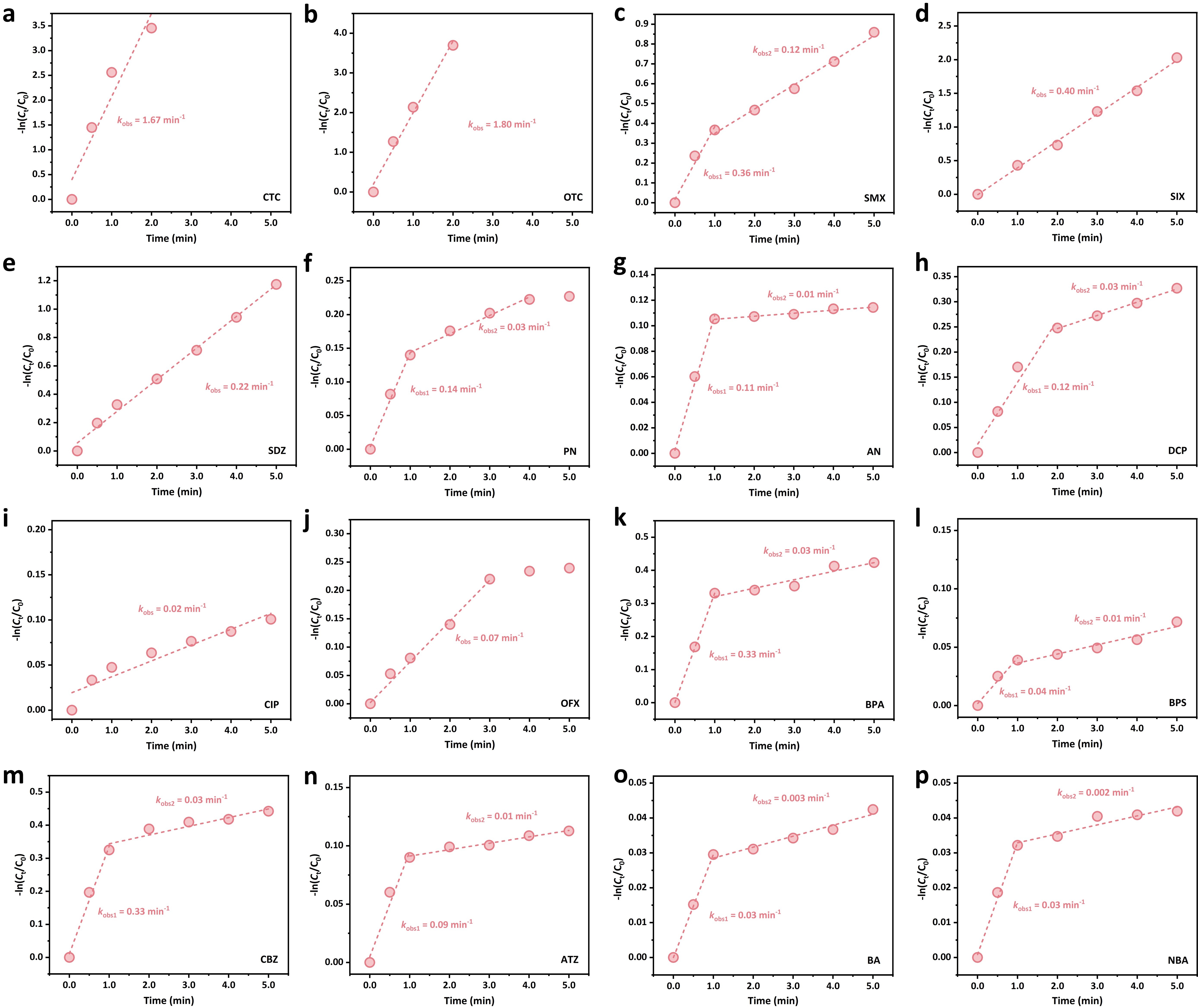


**Figure S52.** The catalytic rates toward (a) CTC, (b) OTC, (c) SMX, (d) SIX, (e) SDZ, (f) PN, (g) AN, (h) DCP, (i) CIP, (j) OFX, (k) BPA, (l) BPS, (m) CBZ, (n) ATZ, (o) BA and (p) NBA.

**Experimental conditions:** [Catalyst] = 0.2 g L^–1^, [Pollutant] = 10.0 mg L^–1^, [PMS] = 0.2 mM.


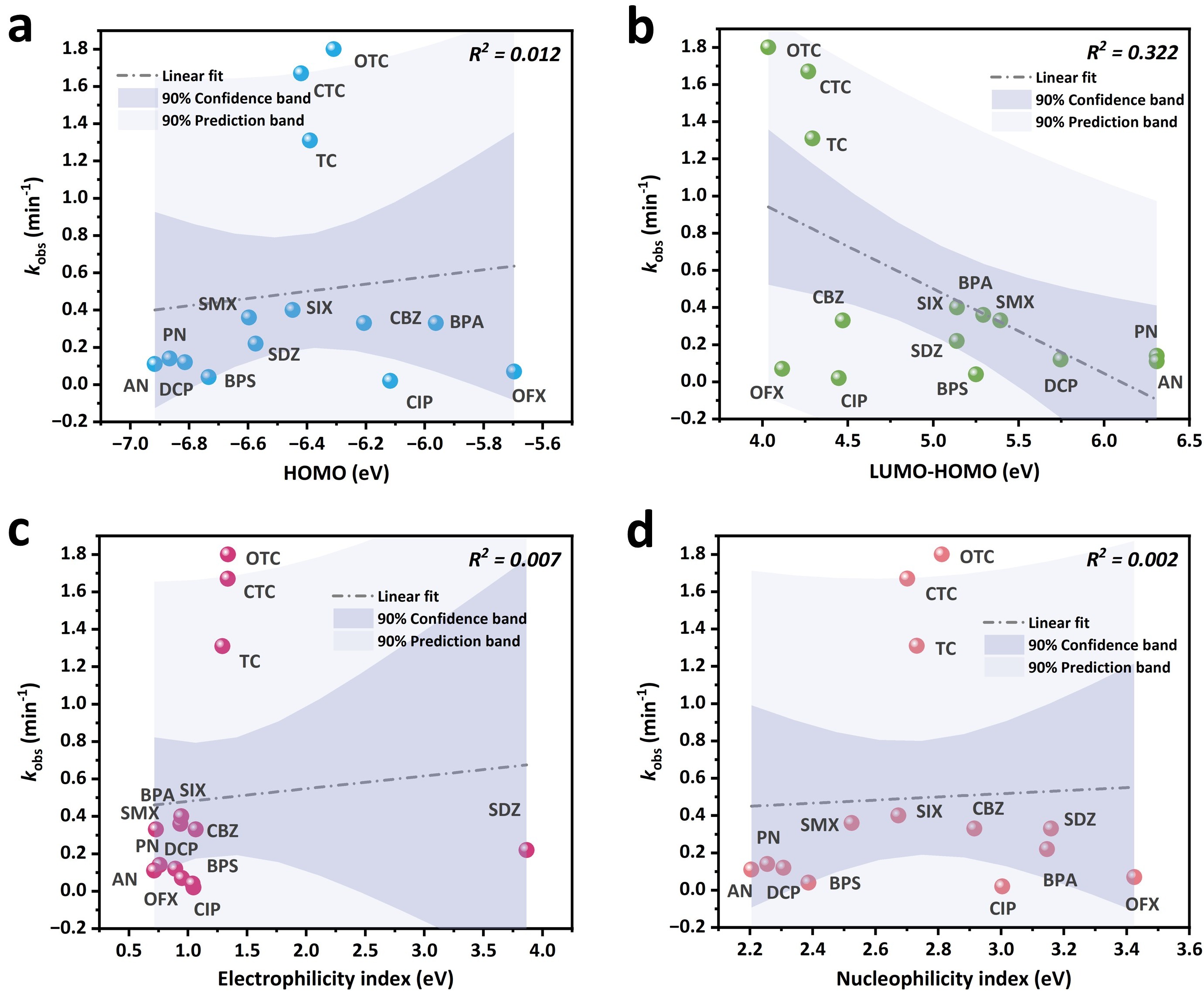


**Figure S53.** The line correlation between organics’ degradation rates and their (a) HOMO, (b) energy gap, (c) electrophilicity index as well as (d) nucleophilicity index.


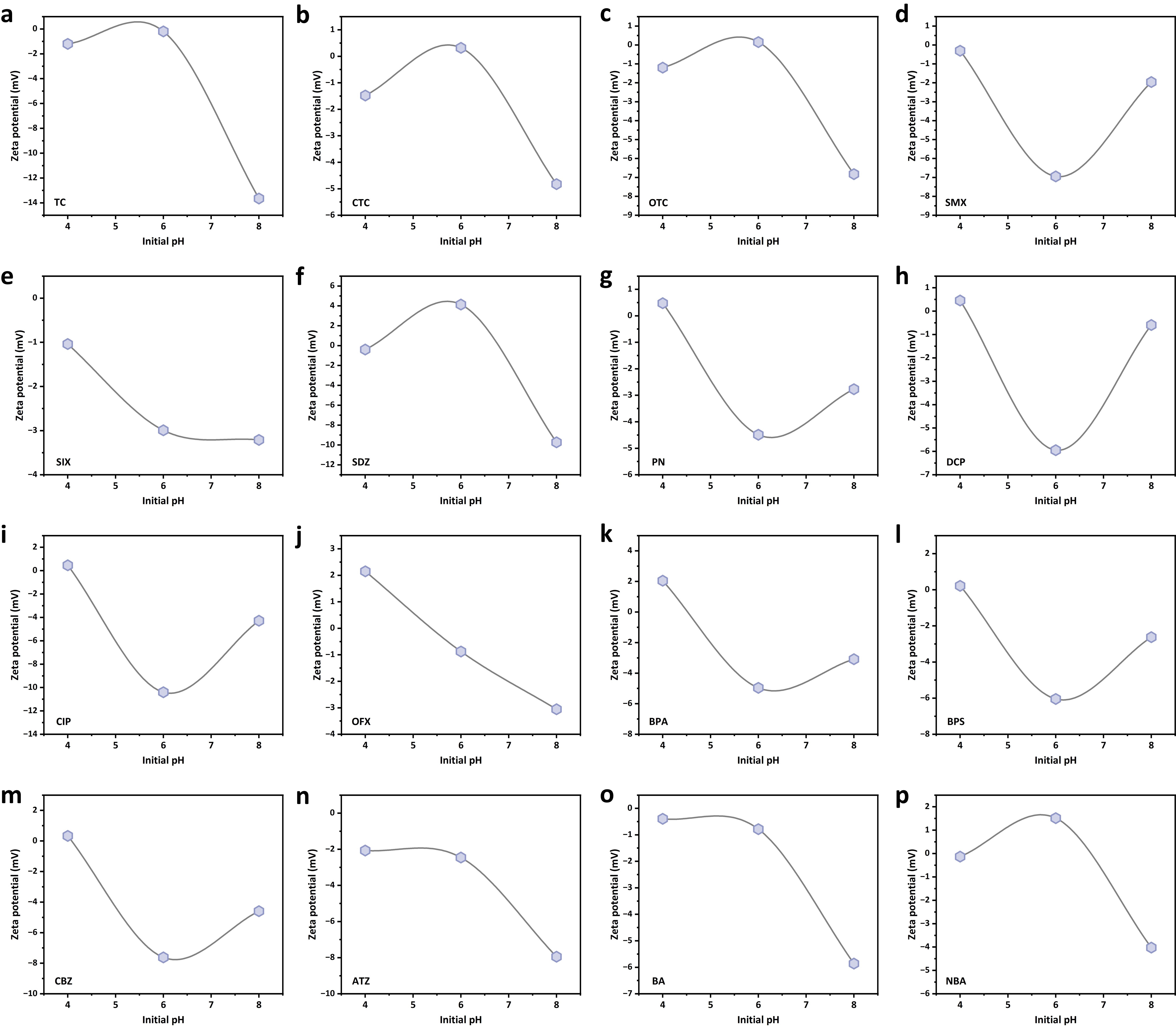


**Figure S54.** Zeta potentials of (a) TC, (b) CTC, (c) OTC, (d) SMX, (e) SIX, (f) SDZ, (g) PN, (h) DCP, (i) CIP, (j) OFX, (k) BPA, (l) BPS, (m) CBZ, (n) ATZ, (o) BA in different pH aqueous solutions.


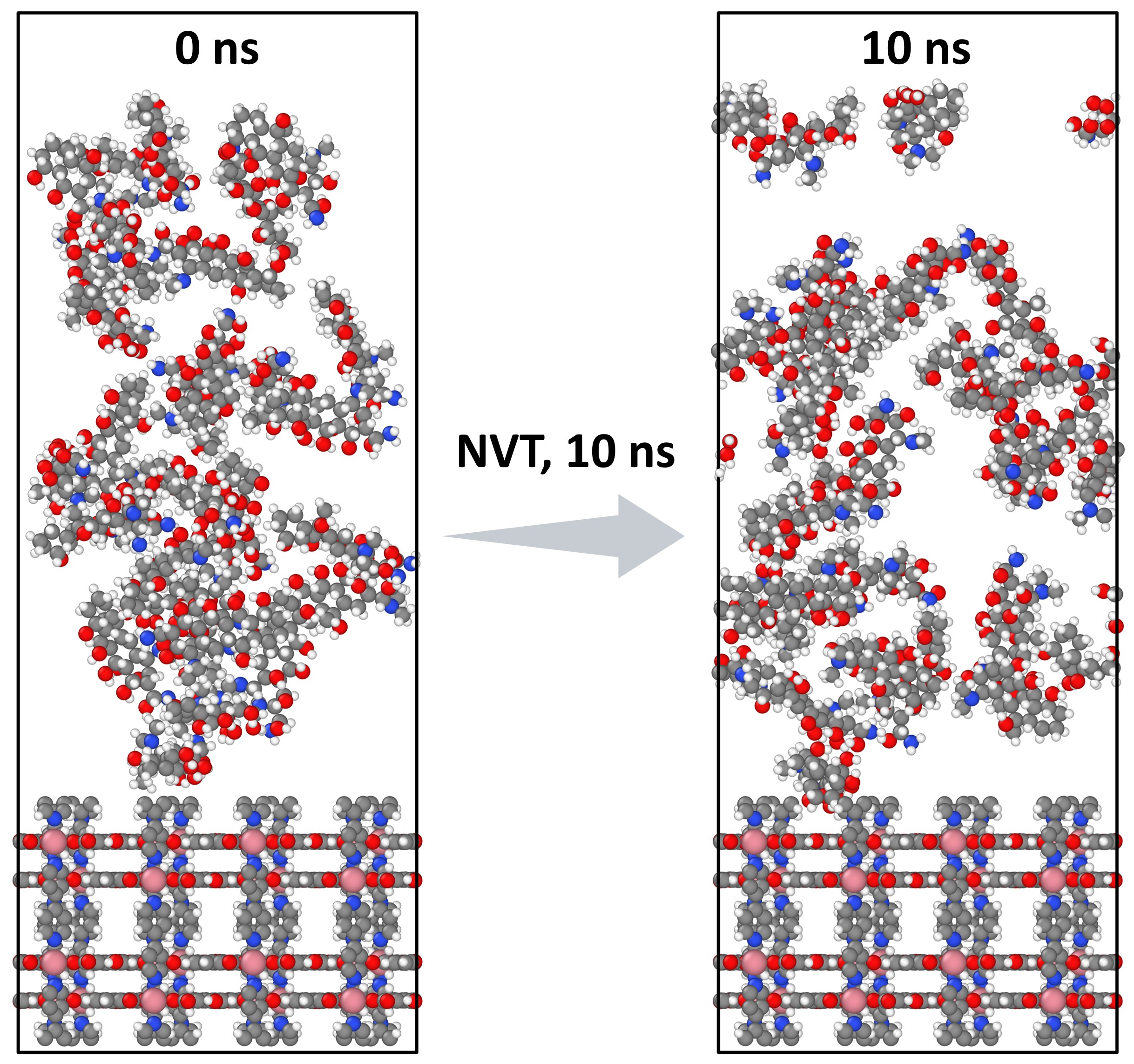


**Figure S55.** Snapshots of the structural models of PKU-24 and TC from molecular dynamics simulations, respectively.


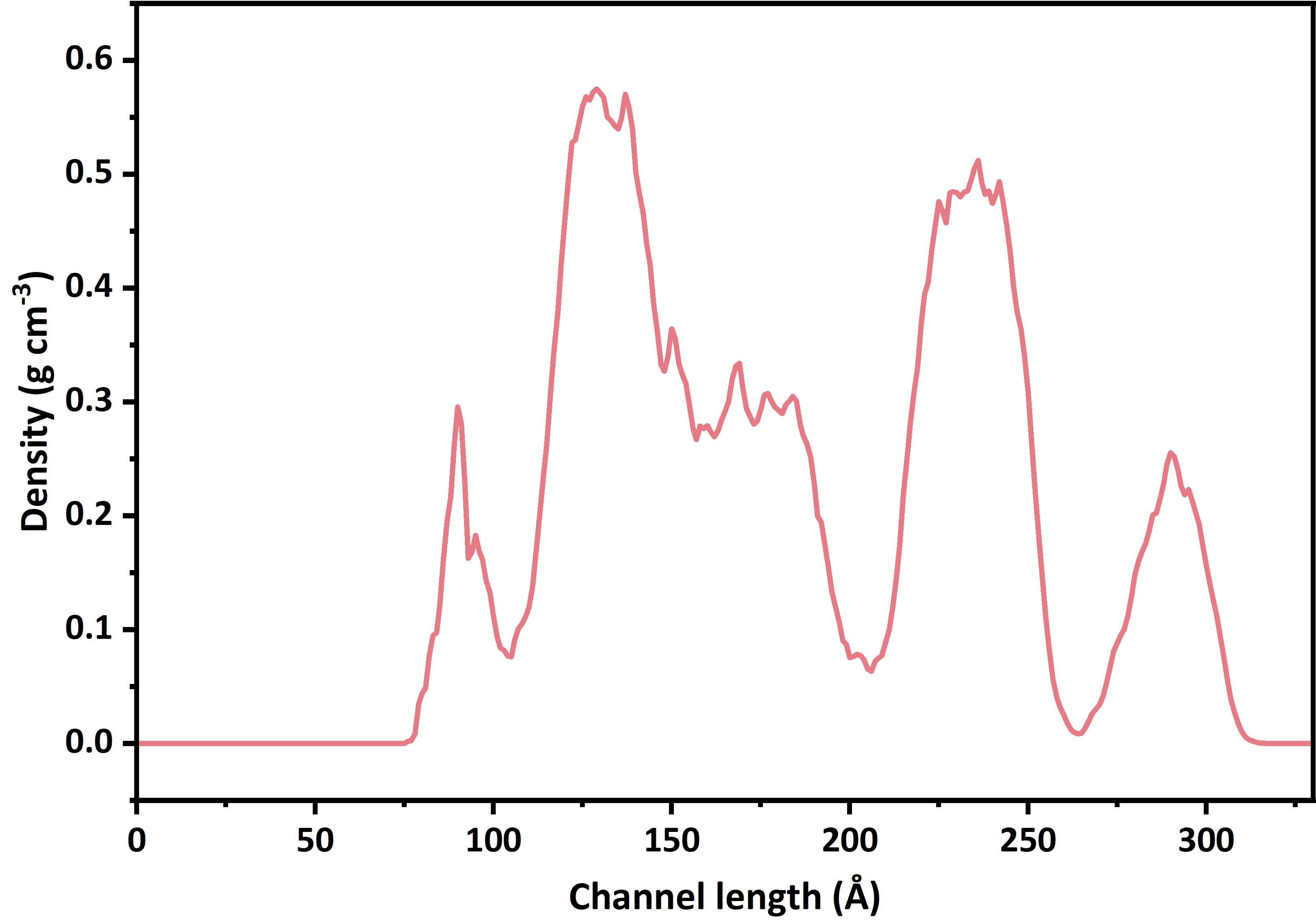


**Figure S56.** The density profile of TC molecules adsorbed on the PKU-24.


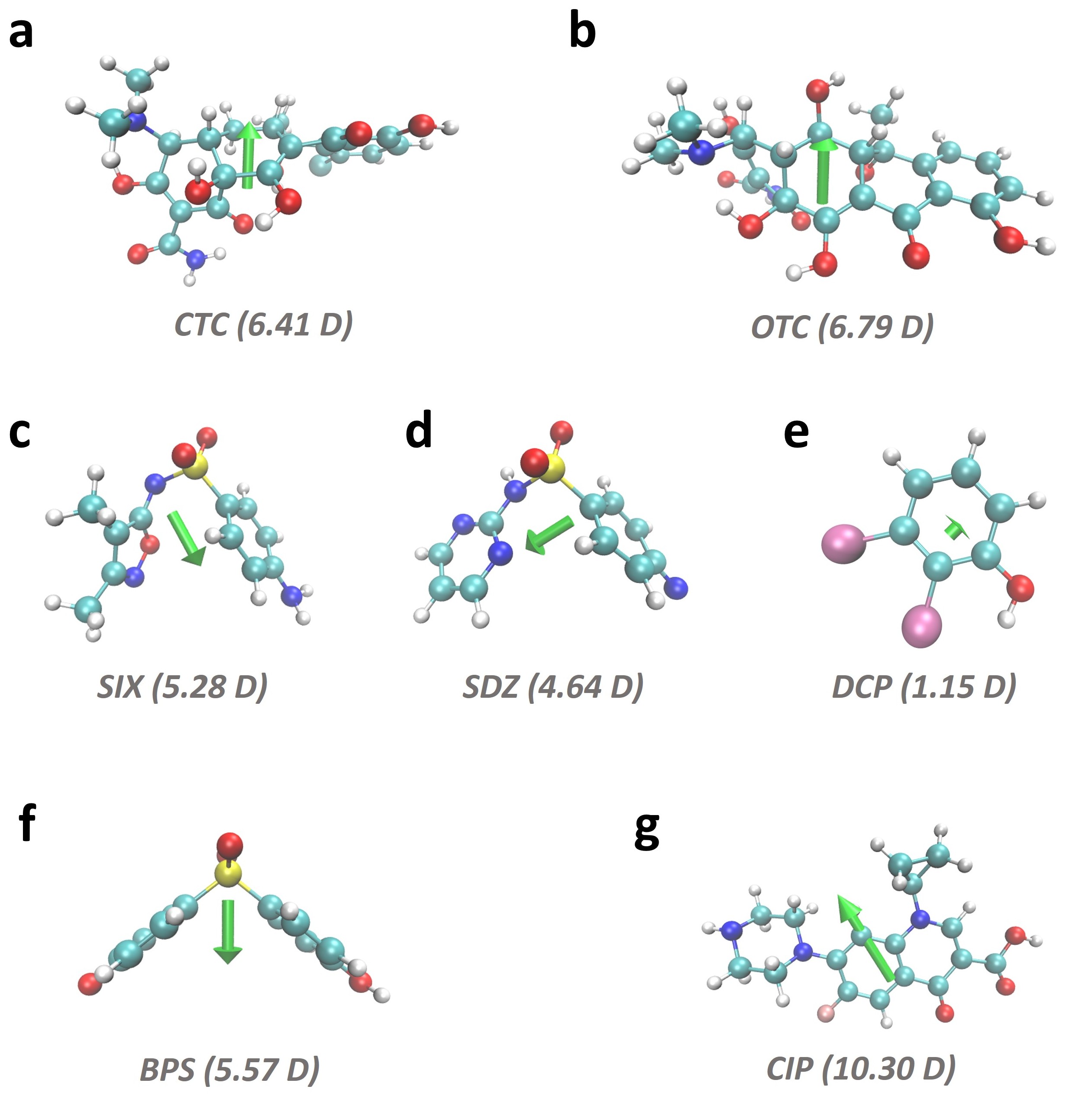


**Figure S57.** The dipole moment of (a) CTC, (b) OTC, (c) SIX, (d) SDZ, (e) DCP, (f) BPS and (g) CIP.


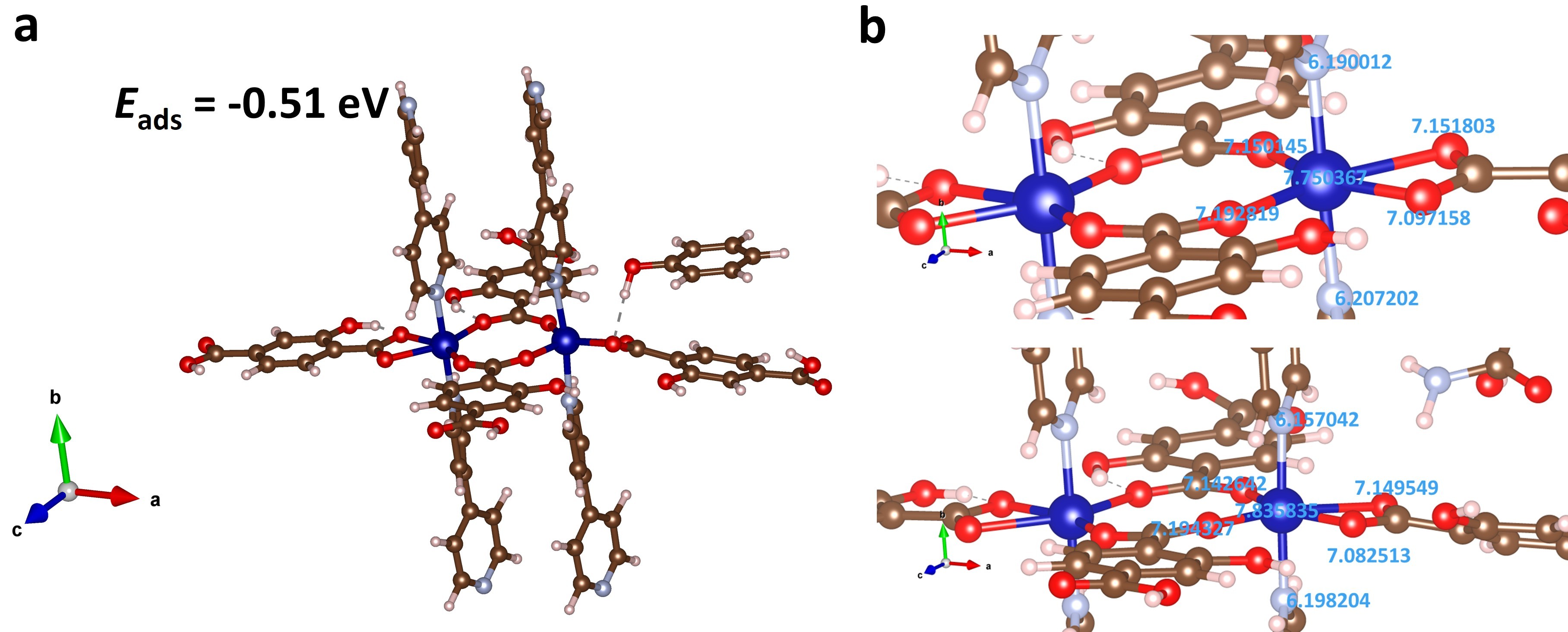


**Figure S58.** (a) PN adsorption model on PKU-24 and sorption energy calculation. (b) The change of the Bader charge of [Co-N_2_O_4_] center before and after PN adsorption.


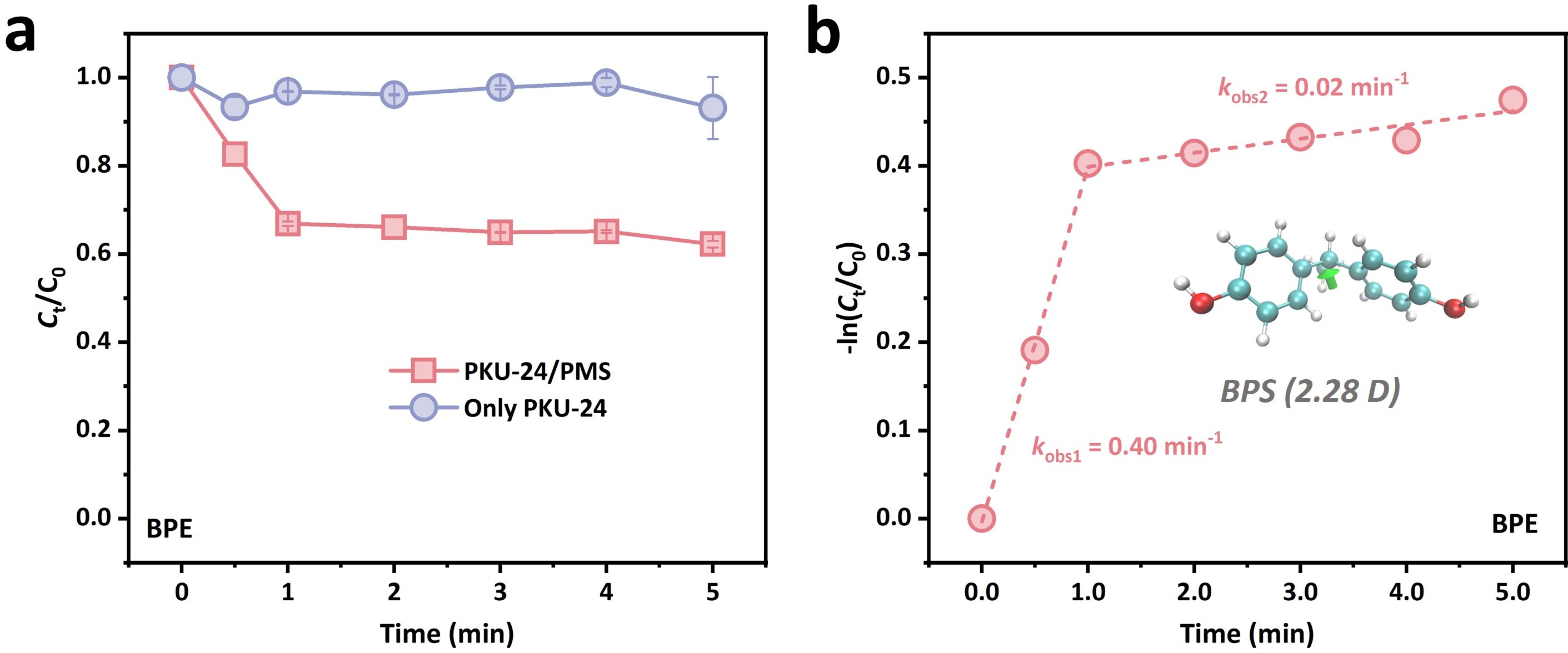


**Figure S59.** The BPE (a) degradation performances in PKU-24/PMS system and (b) the corresponding first-order rate constants. The error bars in the figures represented the standard deviations from triplicate tests.

**Experimental conditions:** [Catalyst] = 0.2 g L^–1^, [BPE] = 10.0 mg L^–1^, [PMS] = 0.2 mM.


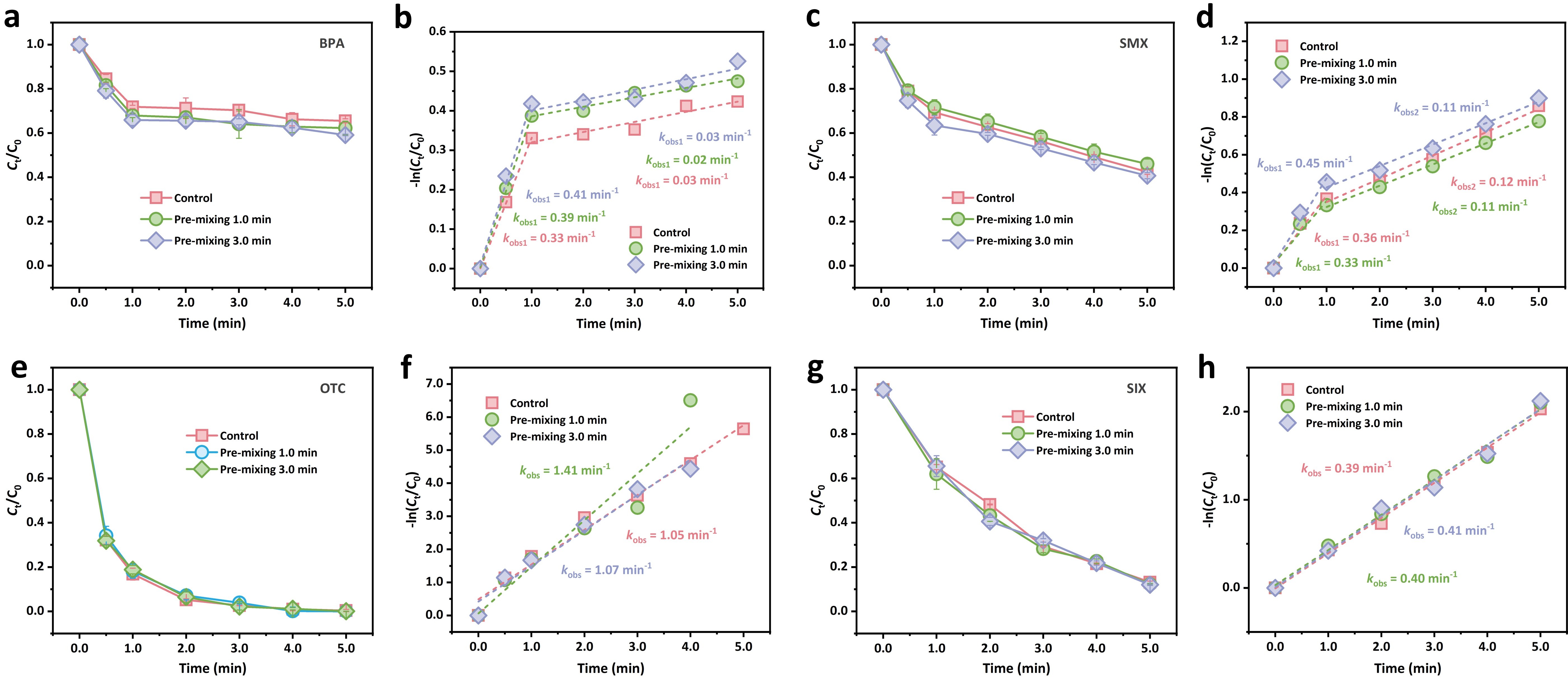


**Figure S60.** (a, b) BPA, (c, d) SMX, (e, f) OTC and (g, h) SIX removal efficiencies after PMS mixing with PKU-24 in advance and their corresponding first-order rate constants. The error bars in the figures represented the standard deviations from triplicate tests.

**Experimental conditions:** [Catalyst] = 0.2 g L^–1^, [BPA] = [SMX] = [OTC] = [SIX] = 10.0 mg L^–1^, [PMS] = 0.2 mM.


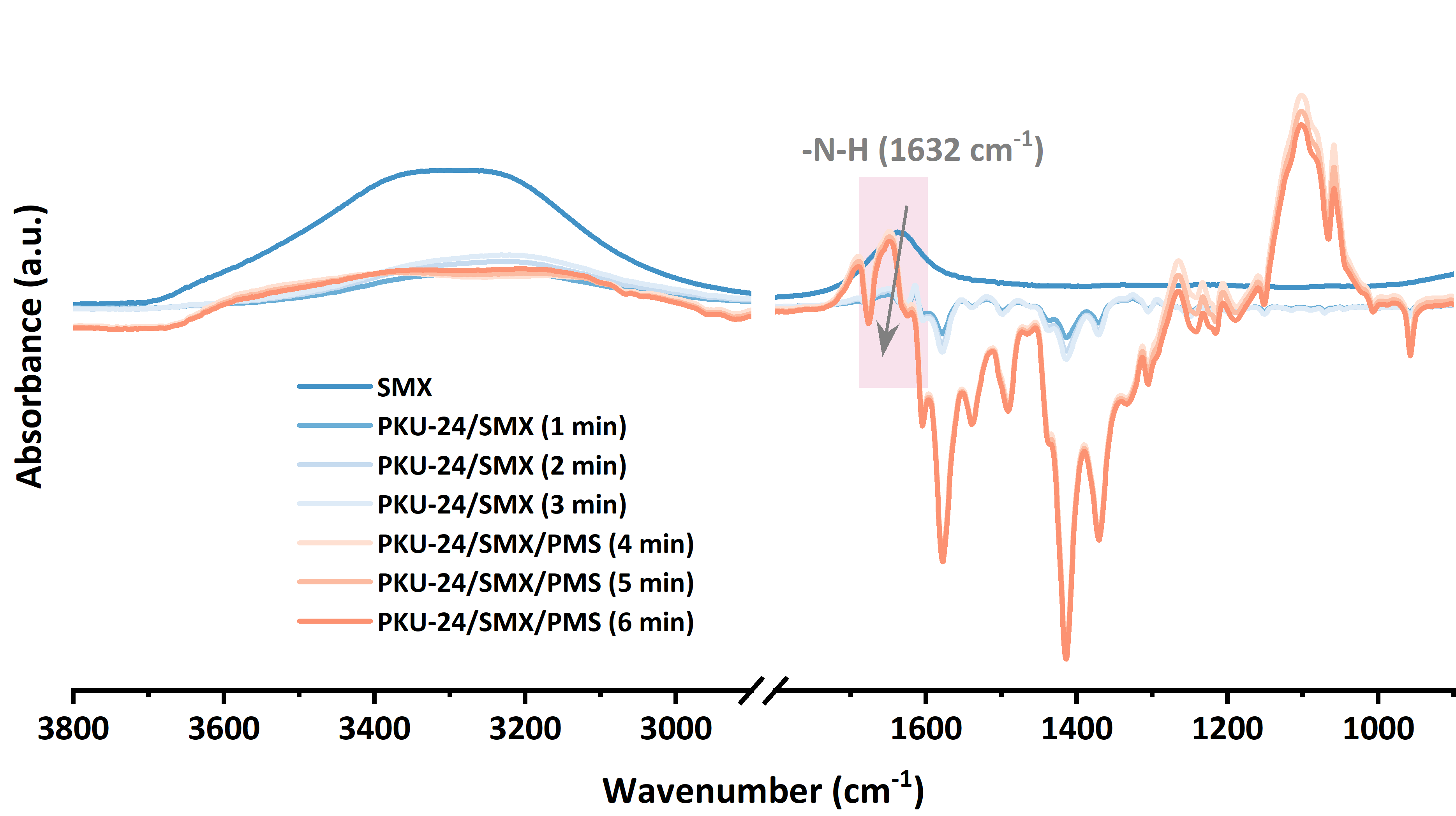


**Figure S61.** In-situ FTIR spectra of PKU-24/PMS/SMX.


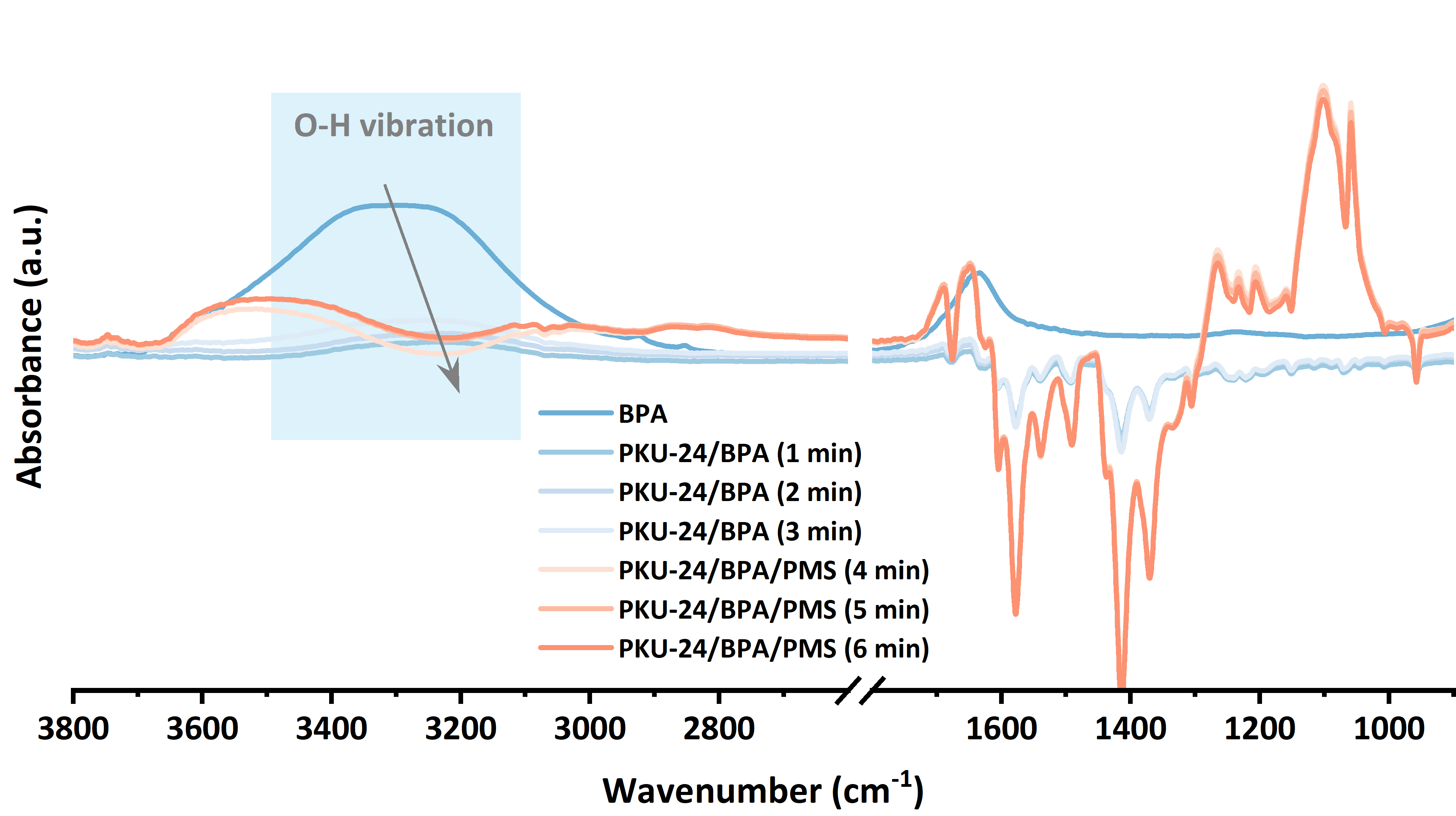


**Figure S62.** In-situ FTIR spectra of PKU-24/PMS/BPA.


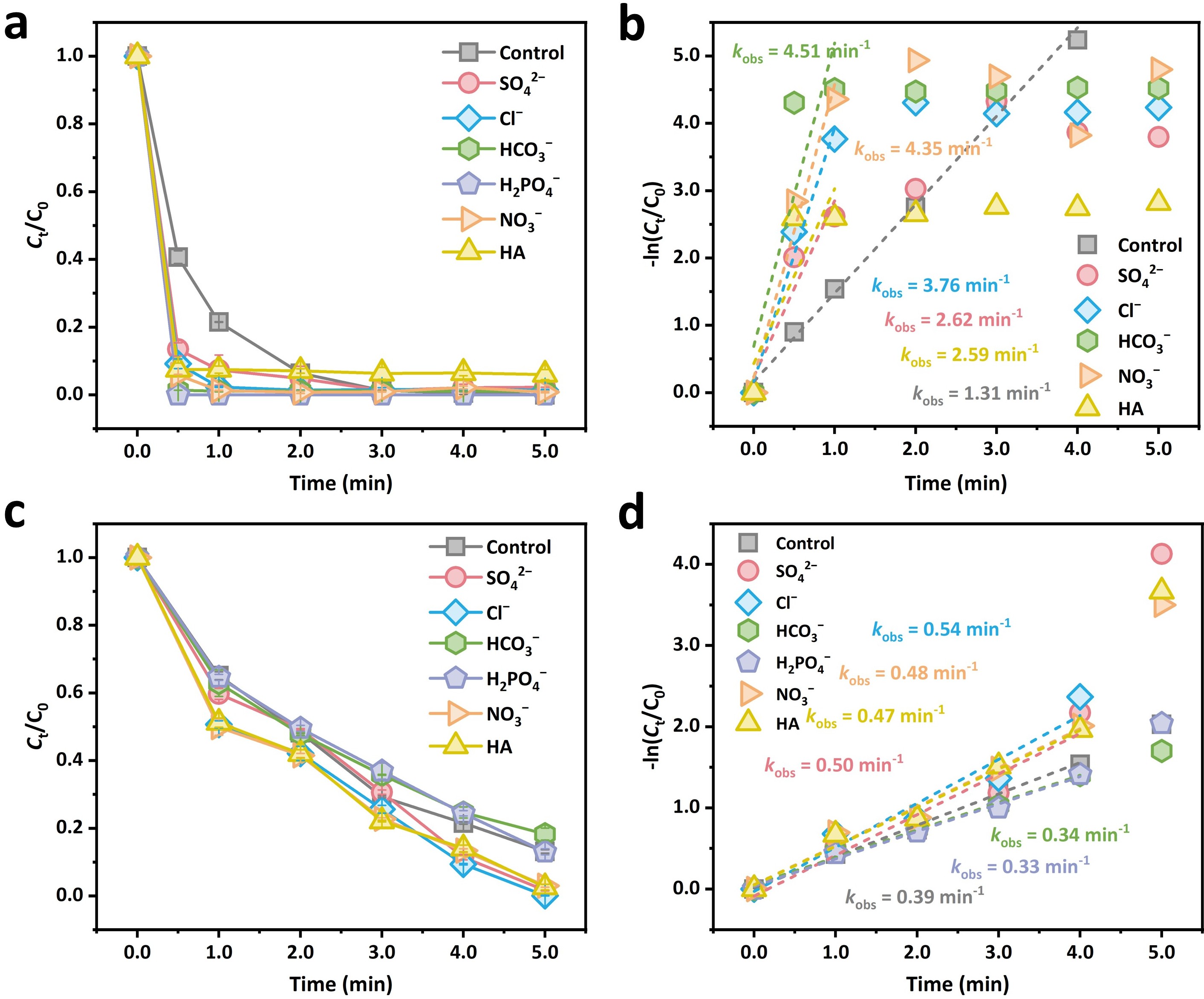


**Figure S63.** Influence of co-existing ions and HA on (a, b) TC and (c, d) SIX removal efficiencies in PKU-24/PMS system as well as their corresponding first-order rate constants. The error bars in the figures represented the standard deviations from triplicate tests.

**Experimental conditions:** [Catalyst] = 0.2 g L^–1^, [PMS] = 0.2 mM, [TC] = [SIX]= 10.0 mg L^–1^.


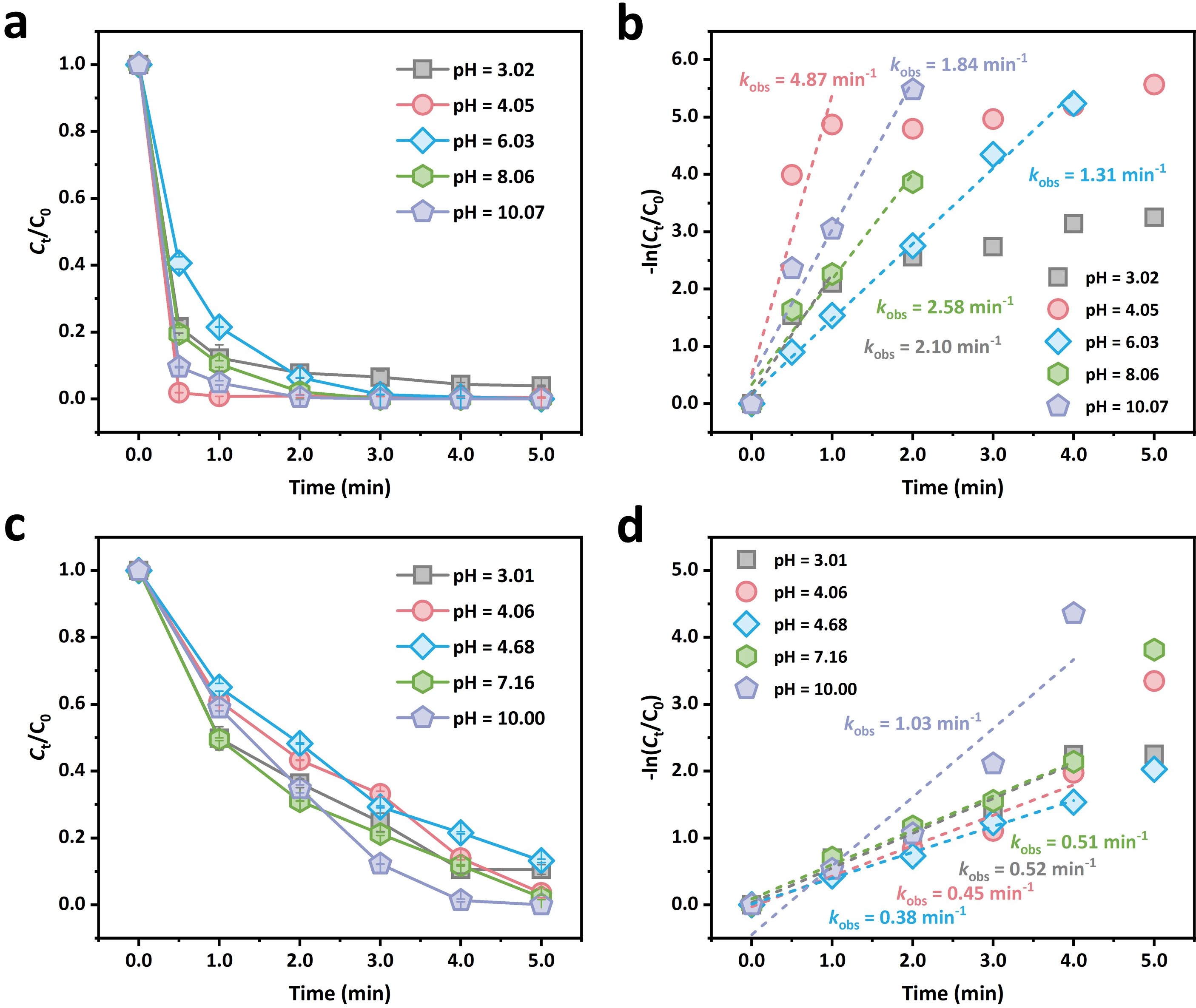


**Figure S64.** Influences of initial pH values on the (a, b) TC and (c, d) SIX removal efficiencies as well as their corresponding first-order rate constants. The error bars in the figures represented the standard deviations from triplicate tests.

**Experimental conditions:** [Catalyst] = 0.2 g L^–1^, [PMS] = 0.2 mM, [TC] = [SIX] =10.0 mg L^–1^.


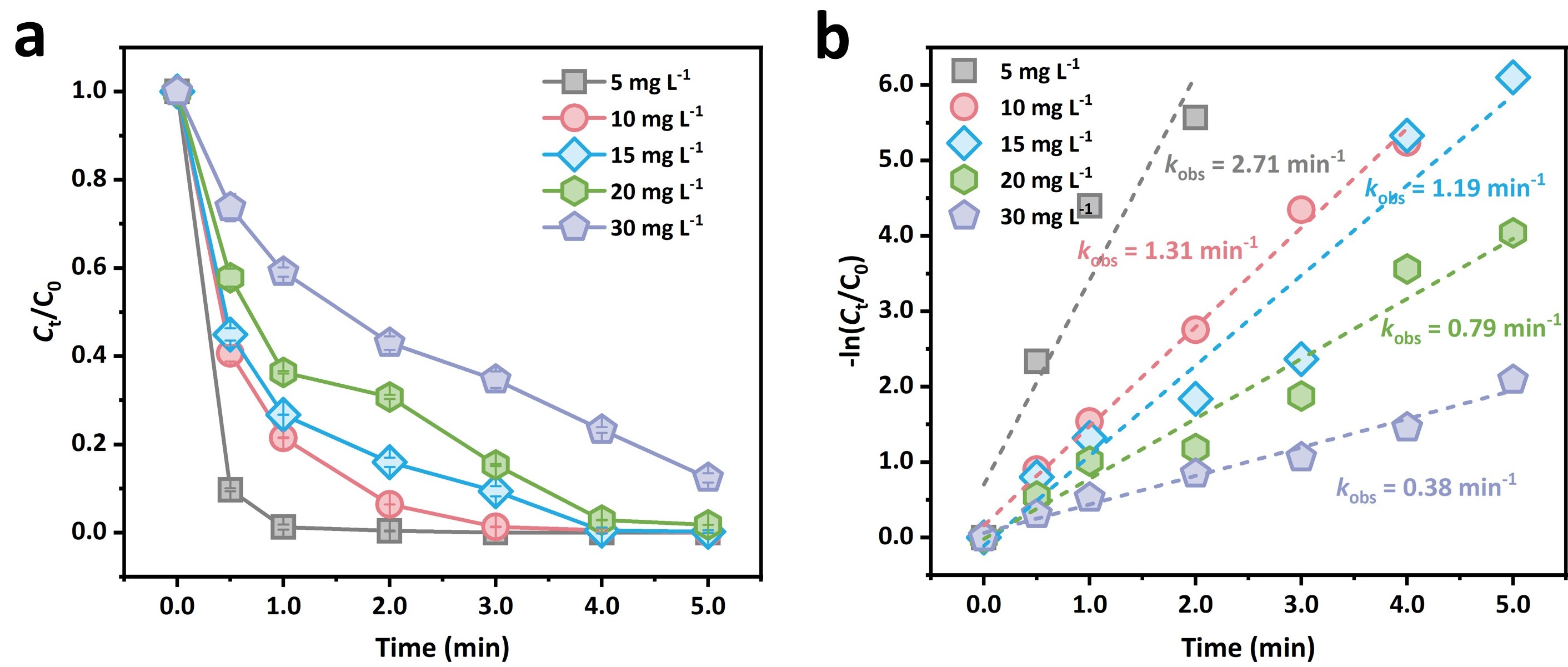


**Figure S65.** Influences of initial TC concentration on the (a) TC removal efficiencies and (b) the corresponding first-order rate constants. The error bars in the figures represented the standard deviations from triplicate tests.

**Experimental conditions:** [Catalyst] = 0.2 g L^–1^, [PMS] = 0.2 mM.


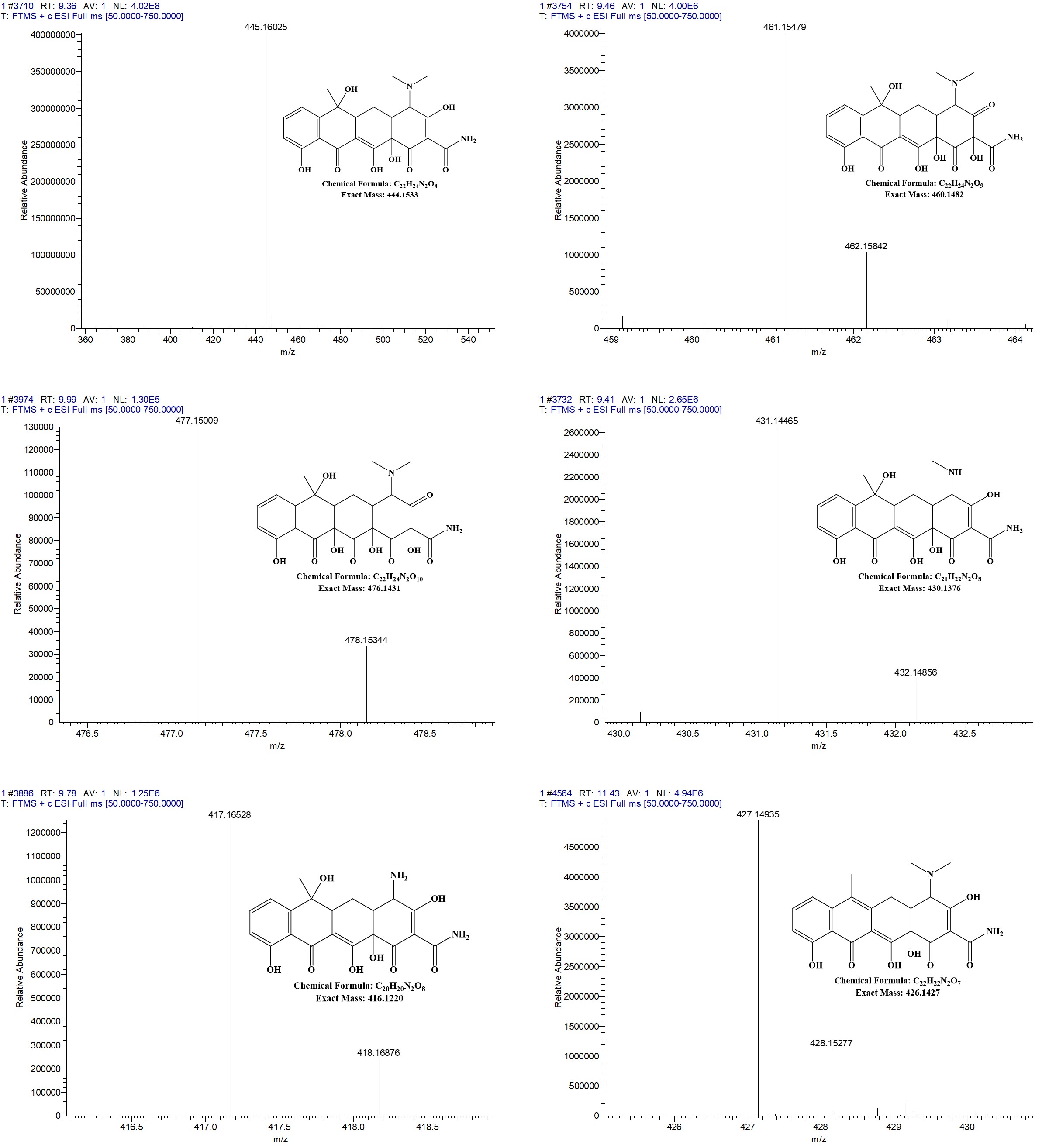


**Figure S66.** The Mass spectra by HPLC-MS/MS (ESI-) and proposed fragmentation pathway.


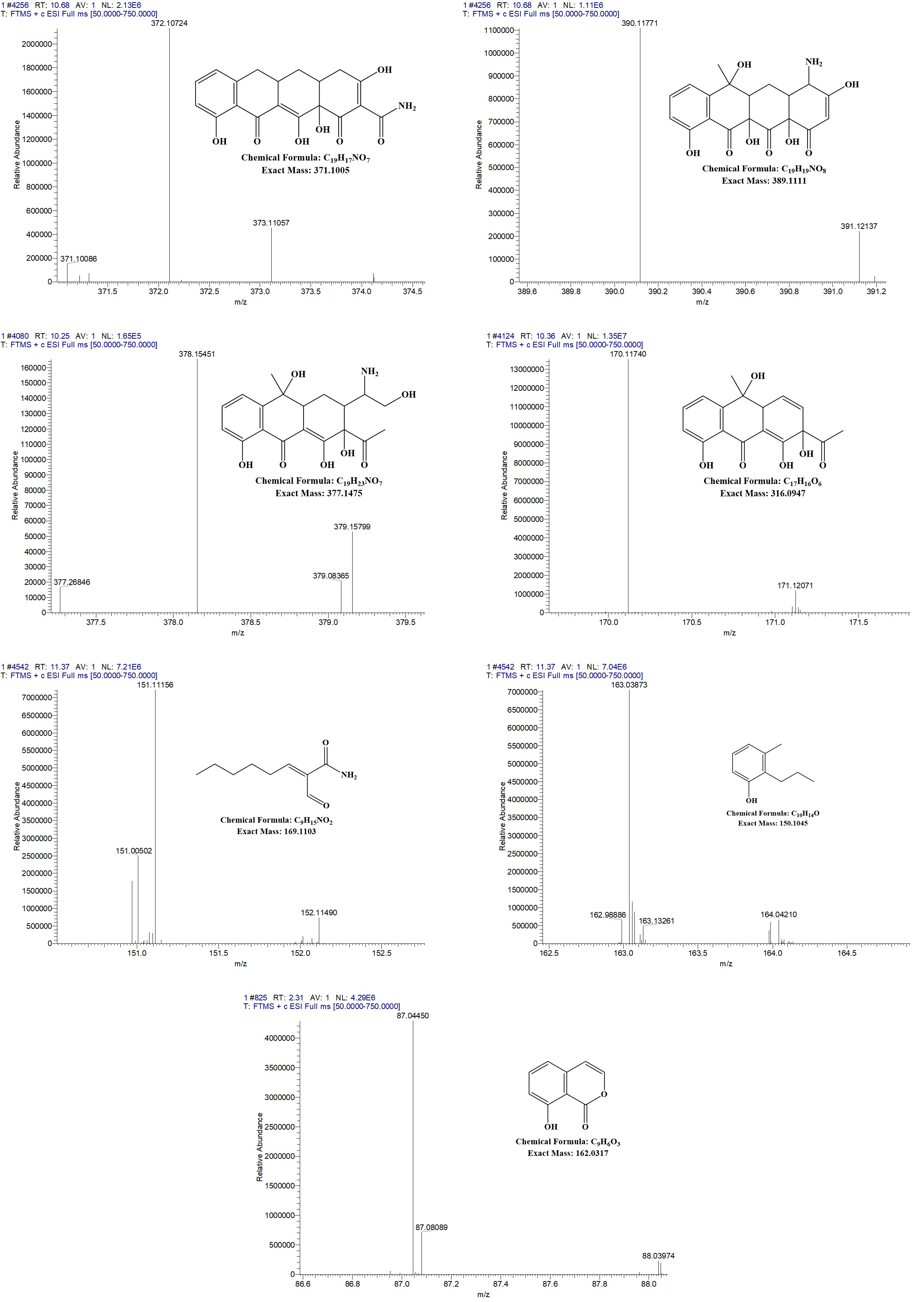


**Figure S67.** The Mass spectra by HPLC-MS/MS (ESI-) and proposed fragmentation pathway.

**Figure S68.** The three possible TC degradation pathways in PKU-24/PMS system.


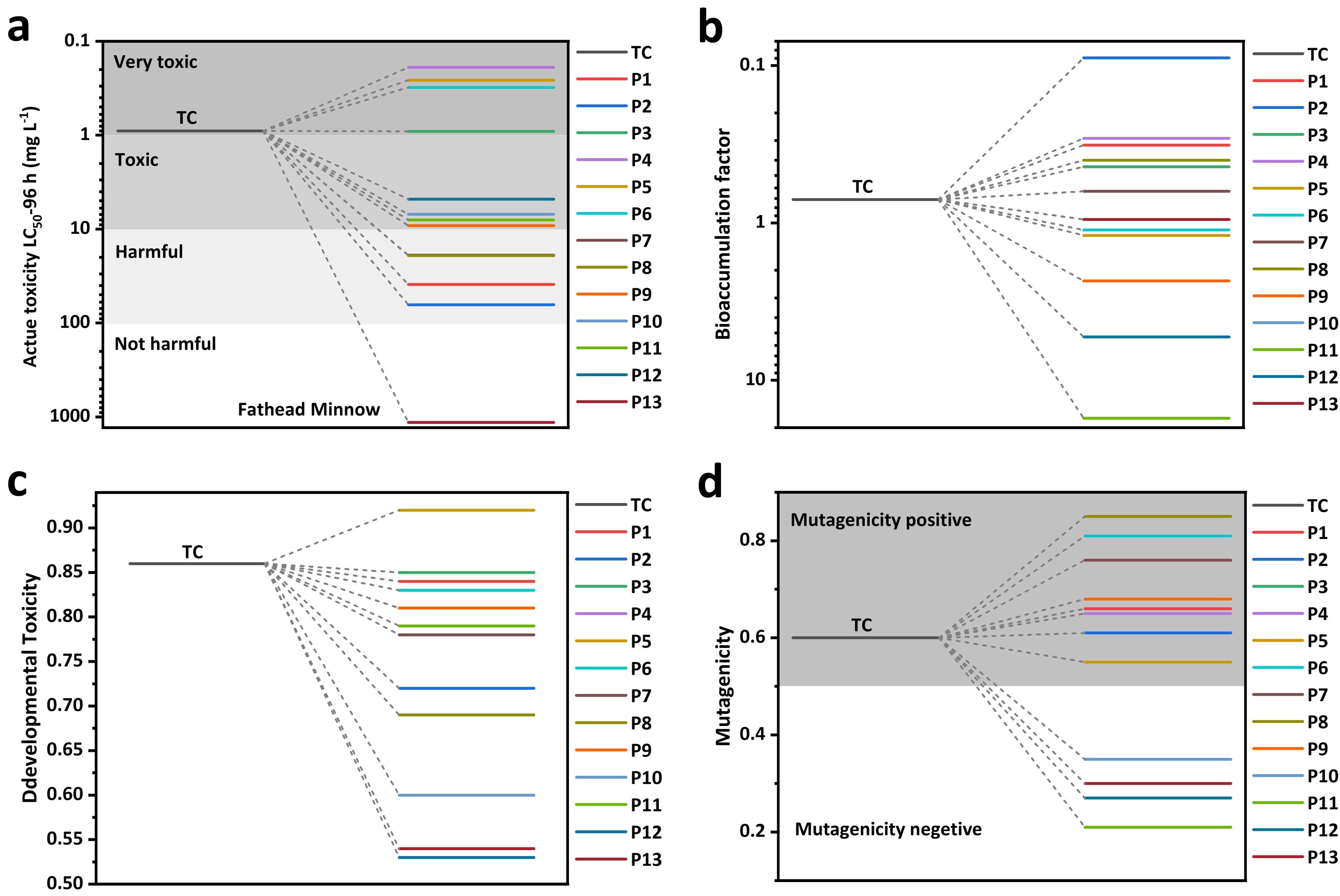


**Figure S69.** (a) Acute toxicity LC_50_ 96h (Fathead minnow), (b) bioaccumulation factor, (c) developmental toxicity and (d) mutagenicity of TC and its degradation intermediates.


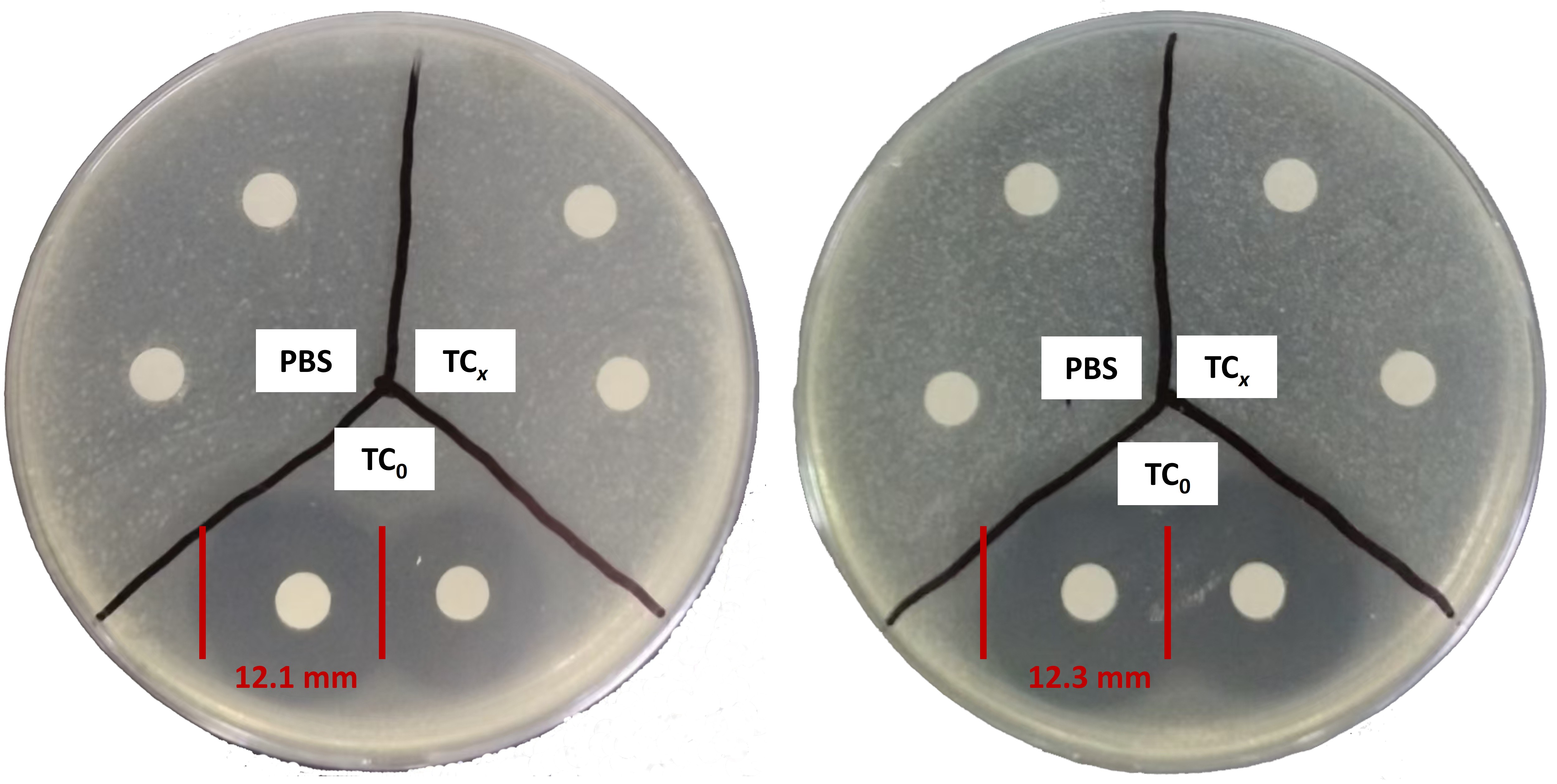


**Figure S70.** Inhibitions of TC and its degradation intermediates on *E. coli* growth.


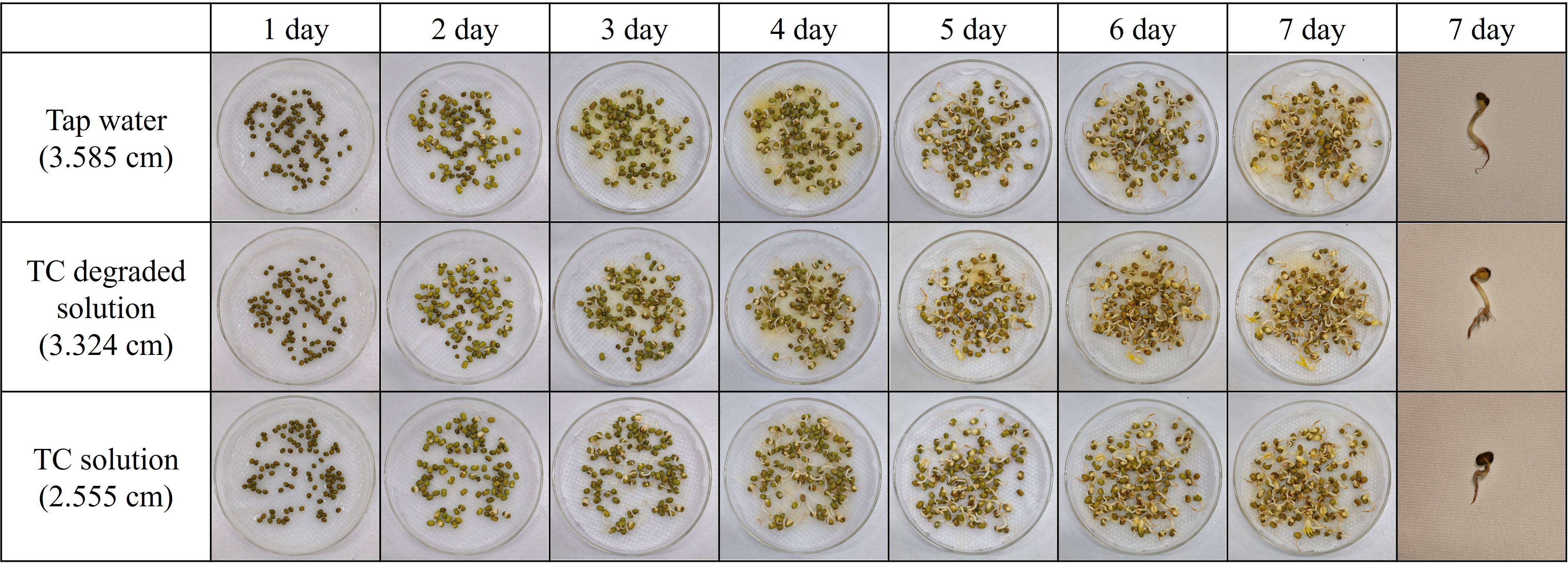


**Figure S71.** The germination rate and growth status of bean sprouts cultivated with tap water, TC-degraded solution and TC solution.


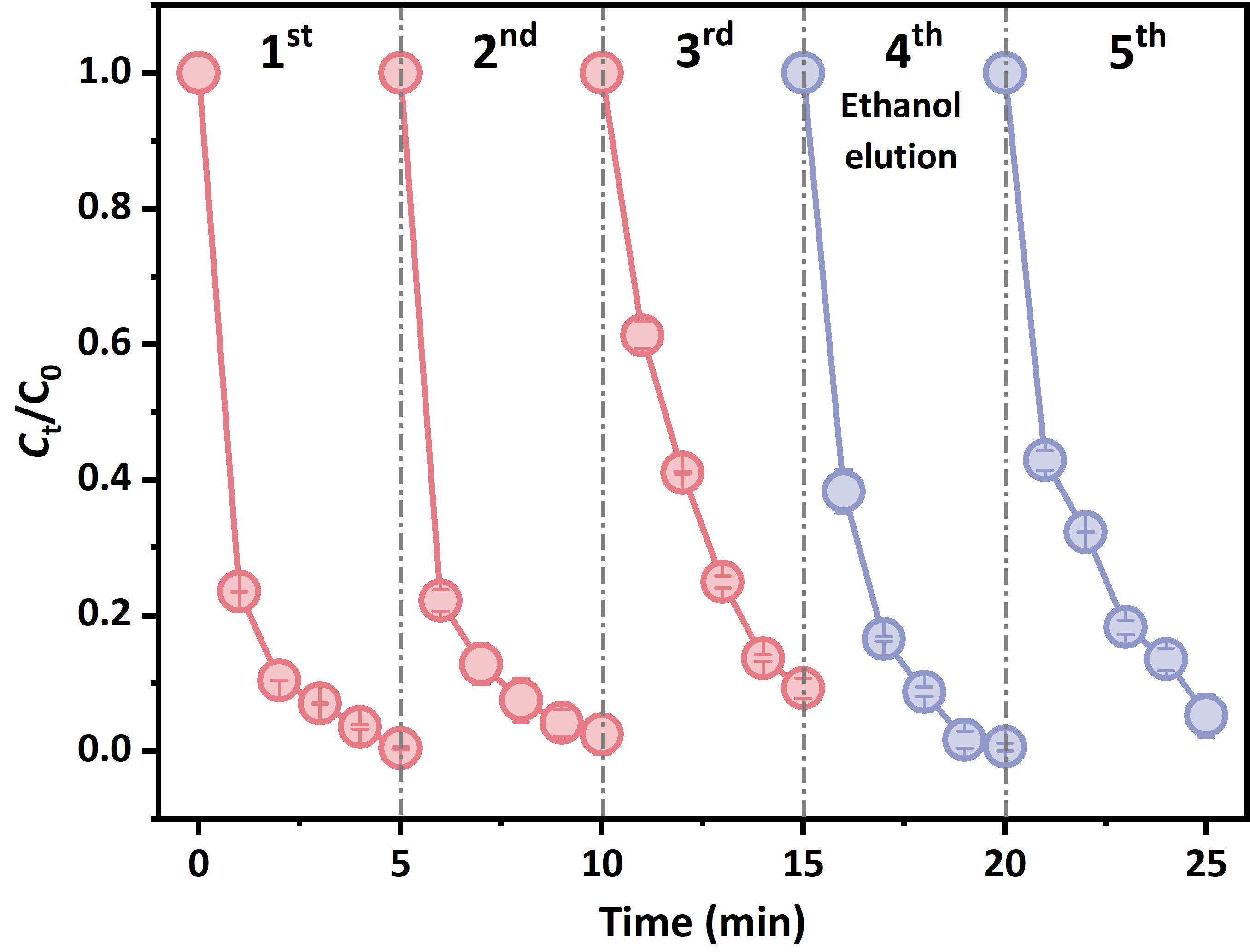


**Figure S72.** The Fenton-like reaction activities of PKU-24 in five consecutive runs.

**Experimental conditions:** [Catalyst] = 0.2 g L^–1^, [PMS] = 0.2 mM, [TC] = 10.0 mg L^–1^, [Initial pH] = 6.03.

**Note:**

After the third catalytic cycle, the used PKU-24 was washed by ethanol to remove the degraded intermediates adsorbed in active sites. Following this regeneration treatment, the TC removal efficiencies in the fourth and fifth cycles were significantly restored, indicating that the activity loss mainly originated from surface fouling rather than permanent deactivation of the catalyst.


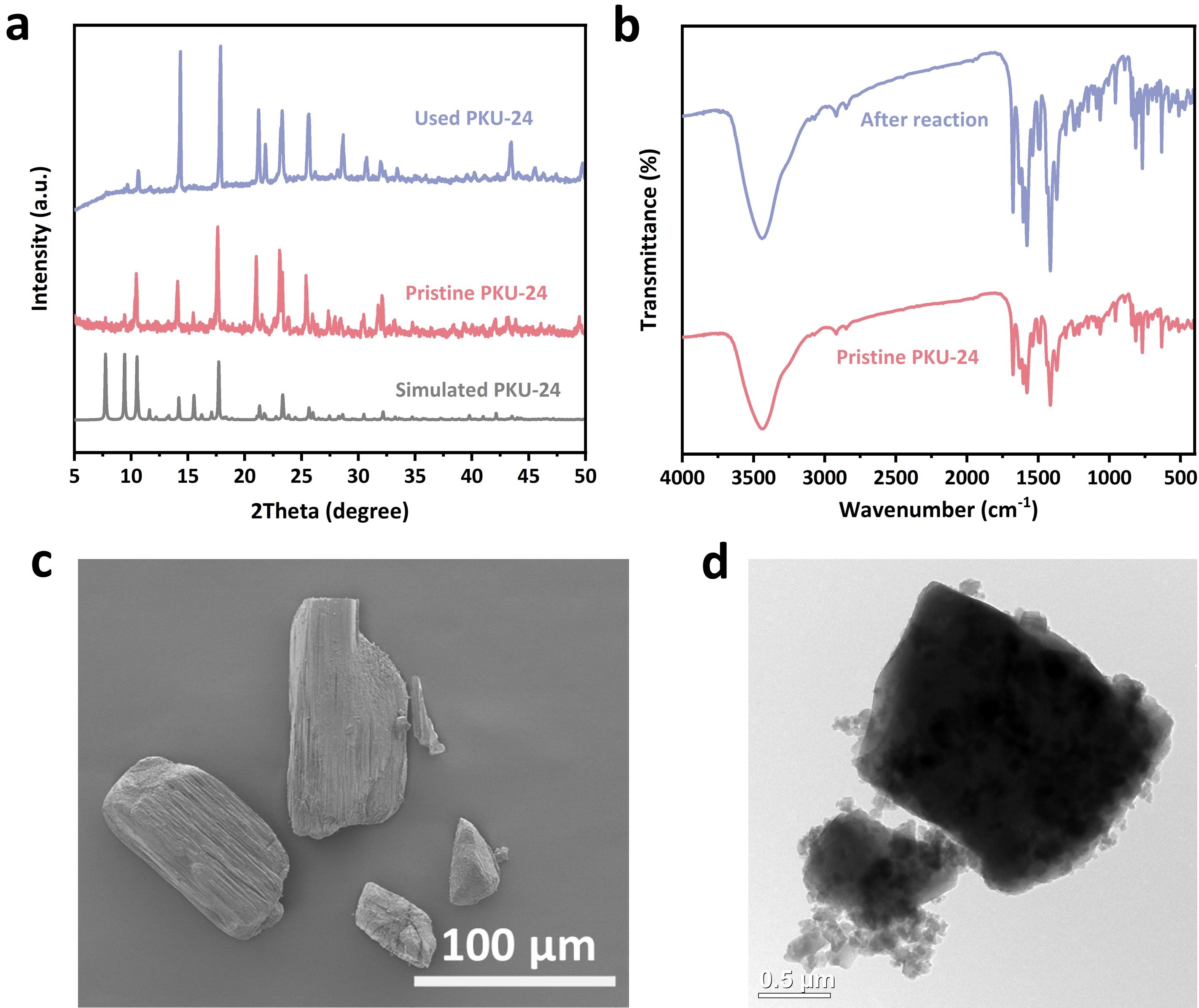


**Figure S73.** The (a) PXRD patterns, (b) FTIR spectrum, (c) SEM image and (d) HR-TEM image of PKU-24 after cyclic experiments.


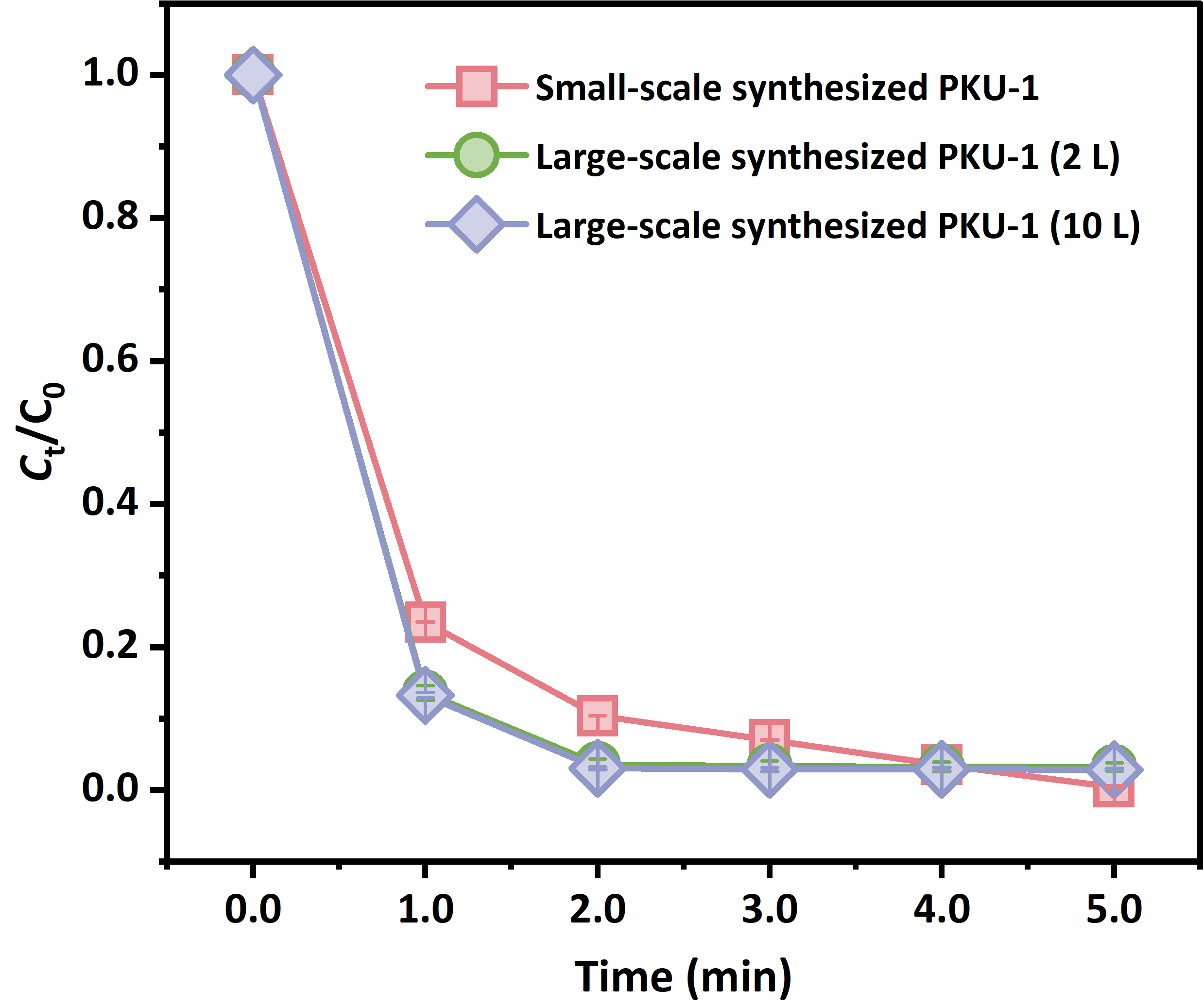


**Figure S74.** The TC degradation activity tests over PKU-24 synthesized at the kilogram scale. The error bars in the figures represented the standard deviations from triplicate tests.

**Experimental conditions:** [Catalyst] = 0.2 g L^–1^, [PMS] = 0.2 mM, [TC] = 10.0 mg L^–1^, [Initial pH] = 6.03.


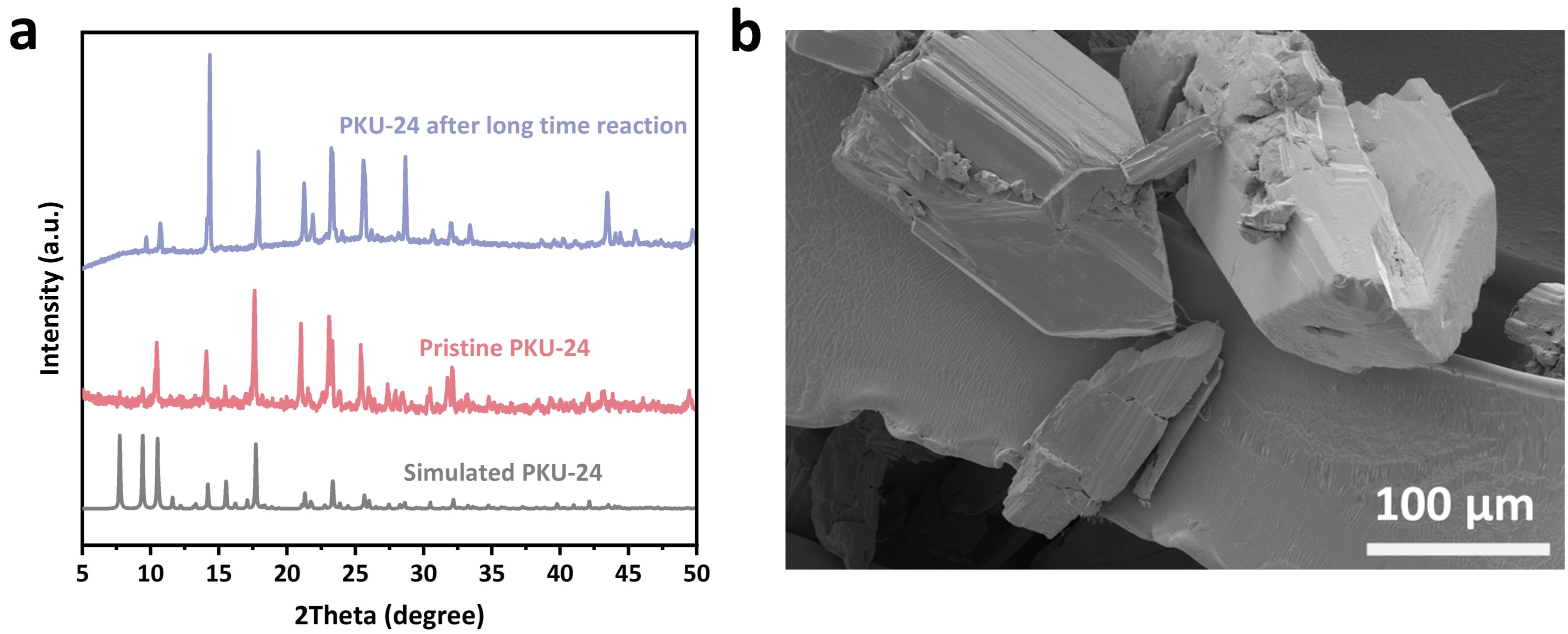


**Figure S75.** The (a) PXRD pattern and (b) SEM image of PKU-24 after long time treatment.


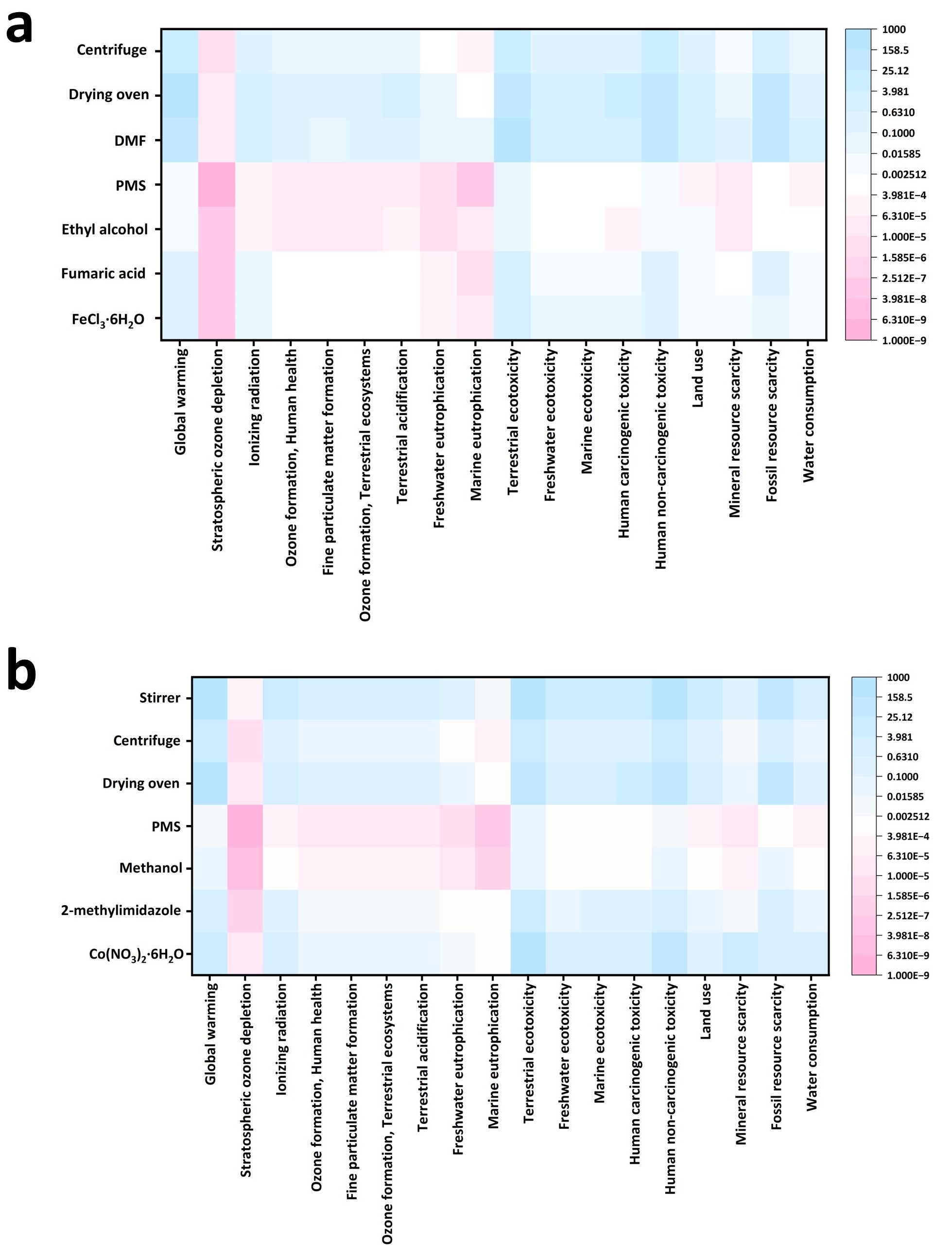


**Figure S76.** Impact assessment for purifying 1.0-ton TC wastewater by (a) MIL-88A/PMS and (b) ZIF-67/PMS.


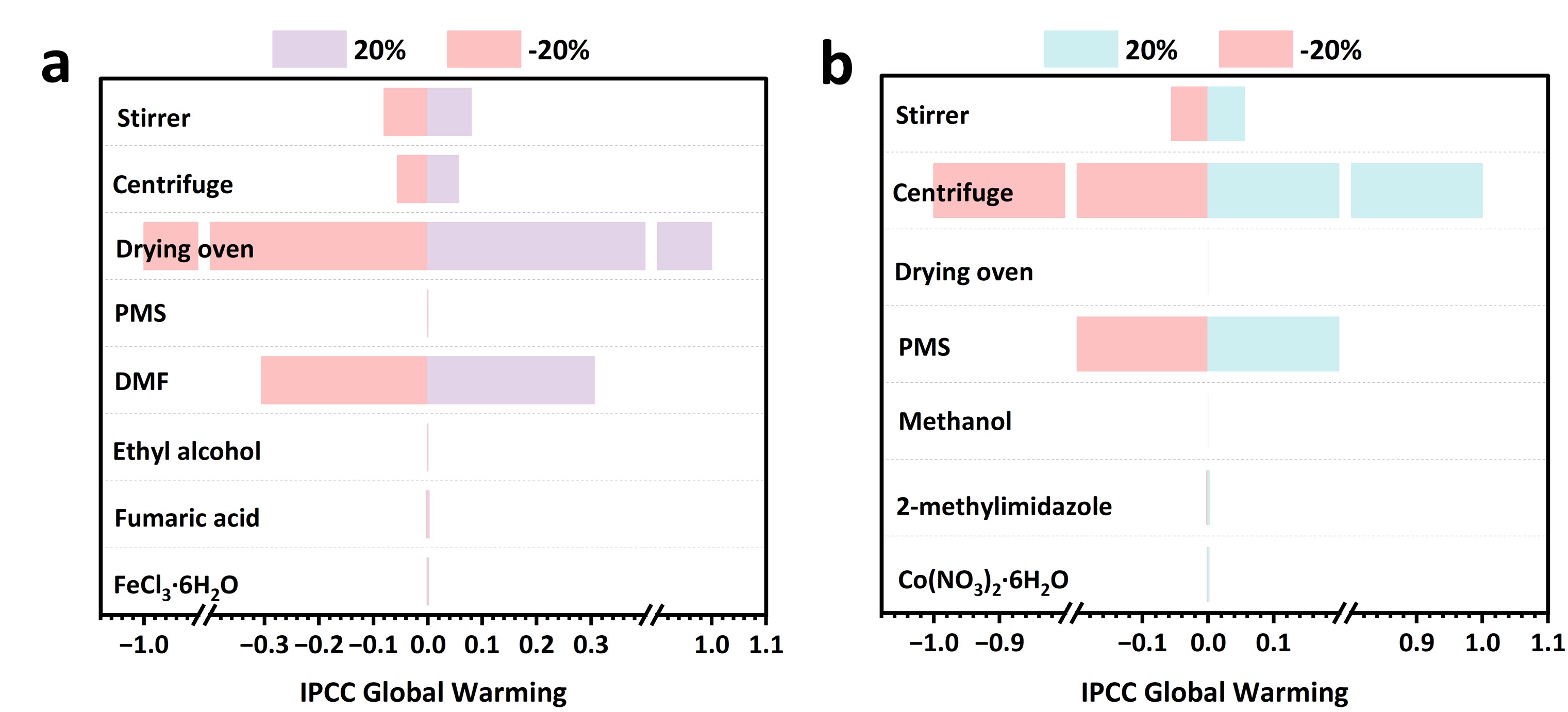


**Figure S77.** The sensitivity analysis of IPCC global warming in (a) MIL-88A/PMS and (b) ZIF-67/PMS.

**Table S1.** The UHPLC analysis conditions for different organic pollutants.

| **Pollutants** | **Flow**  **(mL min^-1^)** | **λ**  **(nm)** | **Acetonitrile (%)** | **CH_3_OH (%)** | **H_2_O**  **(%)** | **phosphoric acid (%)** |
| --- | --- | --- | --- | --- | --- | --- |
| TC | 0.4 | 340 | 20 | NA | NA | 80 |
| CTC | 0.4 | 355 | 18 | NA | 82 | NA |
| OTC | 0.4 | 355 | NA | 35 | NA | 65 |
| SMX | 0.4 | 270 | 45 | NA | NA | 55 |
| SDZ | 0.4 | 255 | 45 | NA | NA | 55 |
| SIX | 0.4 | 271 | NA | 30 | NA | 70 |
| PN | 0.3 | 270 | NA | 40 | 60 | NA |
| AN | 0.4 | 280 | NA | 70 | 30 | NA |
| DCP | 0.5 | 284 | 60 | NA | NA | 40 |
| BPA | 0.4 | 227 | 50 | NA | 50 | NA |
| BPS | 0.2 | 258 | NA | 60 | 40 | NA |
| BPE | 0.25 | 245 | NA | 60 | NA | 40 |
| CIP | 0.3 | 277 | NA | 30 | NA | 70 |
| OFX | 0.4 | 280 | 20 | NA | NA | 80 |
| CBZ | 0.4 | 285 | 50 | NA | NA | 50 |
| ATZ | 0.4 | 220 | 45 | NA | 55 | NA |
| BA | 0.3 | 227 | NA | 50 | NA | 50 |
| NBA | 0.45 | 270 | 50 | NA | NA | 50 |
| PMSO | 0.4 | 215 | 28 | NA | 72 | NA |
| PMSO_2_ | 0.4 | 230 | 28 | NA | 72 | NA |

**Table S2.** The crystal data for PKU-24 in this work (CCDC: 2424707).

| **Compound** | **PKU-24** |
| --- | --- |
| Formula | C_36_H_24_Co_2_N_4_O_10_ |
| Formula weight | 790.45 |
| Space group | *P c c 2* |
| *a*/Å | 18.8002(10) |
| *b*/Å | 11.4172(6) |
| *c*/Å | 16.6030(9) |
| α/° | 90 |
| β/° | 90 |
| γ/° | 90 |
| V/Å^3^ | 3563.8(3) |
| D_c_/g cm^-3^ | 1.473 |

**Table S3.** The EXAFS fitting parameters at the Co K-edge for various samples.

| **Sample** | **Shell** | ***N^a^*** | ***R*(Å)*^b^*** | ***σ*^2^(Å^2^)*^c^*** | **Δ*E*_0_ (eV)*^d^*** | ***R* factor** |
| --- | --- | --- | --- | --- | --- | --- |
| Co foil | Co-Co | 12* | 2.49±0.01 | 0.0064 | 7.5±0.4 | 0.0026 |
| PKU-24 | Co-N/O | 5.3±0.4 | 2.06±0.01 | 0.0084 | -1.3±1.8 | 0.0167 |
|  | Co-C | 7.7±2.3 | 3.05±0.03 | 0.0138 |  |  |
| CoO | Co-O | 6.3±0.7 | 2.10±0.01 | 0.0095 | -2.8±1.1 | 0.0118 |
|  | Co-Co | 13.0±0.7 | 3.01±0.01 | 0.0083 |  |  |
| Co_3_O_4_ | Co-O | 5.3±0.3 | 1.91±0.01 | 0.0029 | -9.1±0.7 | 0.0057 |
|  | Co-Co | 4.8±0.2 | 2.86±0.01 | 0.0044 |  |  |
|  | Co-Co1 | 7.9±0.4 | 3.36±0.01 | 0.0058 |  |  |

**Note:**

*^a^N*: coordination numbers; *^b^R*: bond distance; *^c^σ*^2^: Debye-Waller factors; *^d^* Δ*E*_0_: the inner potential correction. *R* factor: goodness of fit. *Ѕ*_0_^2^ was set to 0.76, according to the experimental EXAFS fit of Co foil reference by fixing CN as the known crystallographic value.

**Table S4.** Comparison of the kinetics of organic contaminant degradation in recently reported Fenton-like processes.

| **Catalyst (g L^-1^)** | **PMS (mM)** | **Pollutant (mg L^-1^)** | **Removal efficiency** | ***k*_obs_**  **(min^-1^)** | ***k*-value**  **(min^-1^·M^-1^)** | **Mechanism** | **Ref.** |
| --- | --- | --- | --- | --- | --- | --- | --- |
| PKU-24 (0.2) | 0.2 | TC (10) | 99.6% (5 min) | 1.31 | 327.5 | ^1^O_2_ | This work |
| re-7Mn-CeO_2_ NMs (0.1) | 0.3 | TC (13.32) | 100% | 0.363 | 161.2 | Radicals | [12] |
| Co-CN (0.5) | 0.8 | TC (10) | 100% | 0.194 | 4.85 | Radicals, ^1^O_2_ | [13] |
| B-Mn (0.4) | 1.6 | TC (10) | 99.4% | 0.105 | 1.64 | Radicals, ^1^O_2_ | [14] |
| 70% Fe_3_O_4_ (0.4) | 2.0 | TC (20) | 90.12% | 0.02193 | 0.55 | ^1^O_2_ | [15] |
| Co_3_O_4_/BC (0.1) | 0.65 | TC (40) | 90% | 0.251 | 154.5 | Radicals, nonradical | [16] |
| BOB-1/FeOOH (0.2) | 0.24 | TC (20) | 96% | 0.0762 | 31.75 | Radicals, ^1^O_2_ | [17] |
| MoS_2_/Ag/g-C_3_N_4_ (0.2) | 0.1 | TC (20) | 91.2% | 0.0837 | 83.7 | Radicals | [18] |
| Co/Fe doped g-C_3_N_4_ (0.1) | 0.65 | TC (20) | 86.7% | 0.06 | 18.5 | Radicals, ^1^O_2_ | [19] |
| Cu-doped hematite (0.2) | 0.45 | TC (40) | 97.8% | 0.049 | 8.71 | Radicals, ^1^O_2_ | [20] |
| NiCo_2_O_4_/g-C_3_N_4_ (0.1) | 1.0 | TC (250) | 99% | 0.0117 | 29.25 | Radicals, ^1^O_2_ | [21] |
| FeVO_4_ (0.8) | 1.0 | OTC (20) | 100% | 0.1073 | 2.68 | Radicals, nonradical | [22] |
| MAC (0.1) | 0.5 | OTC (20) | 100% | 0.0414 | 16.56 | ^1^O_2_ | [23] |
| 15-NFO@CuO (0.2) | 1.25 | OTC (40) | 100% | 0.06362 | 10.2 | SO_4_^•−^, ^•^OH | [24] |
| Co_9_S_8_@S-N-RG (0.2) | 0.8 | SMX (10.1) | 100% | 0.377 | 0.596 | Radicals | [25] |
| CoSx@N-S-O-C-5 (0.1) | 0.8 | SMX (10.1) | 100% | 0.384 | 48.63 | Radicals | [26] |
| Fe@N-CNs (0.15) | 0.5 | SMX (10) | 100% | 0.39 | 52 | Radicals, ^1^O_2_ | [27] |
| DSLB-800 (2.0) | 2.5 | SMX (15) | 96.6% | 0.0299 | 0.0897 | Radicals, ^1^O_2_ | [28] |
| Co–Mo–TiO_2_ (0.1) | 1.0 | SMX (10) | 100% | 0.0991 | 9.91 | Radicals, ^1^O_2_ | [29] |
| Ag_0.06_-CoFe_2_O_4_ (0.1) | 0.1 | SMX (2.53) | 95.1% | 0.098 | 24.8214 | Radicals, ^1^O_2_ | [30] |
| MnO_X_-180 (0.5) | 1 | SMX (10) | 97% | 0.0223 | 0.446 | ^1^O_2_ | [31] |
| OMC-Co-T800 (0.1) | 0.65 | SMX (10) | 99.94% | 0.1988 | 30.538 | Radicals, ^1^O_2_ | [32] |
| N-GA-2 (0.1) | 0.8 | SMX (15) | 96.32% | 0.0231 | 3.773 | Electron-transfer | [33] |
| CoSA-NC (0.15) | 0.3 | SIX (5) | 100% | 0.367 | 40.7 | ^1^O_2_ | [34] |

**Note:**

*k*-value = (*k*_obs_ × *c*[pollutant])/(*c*[catalyst] × *c*[PMS])

**Table S5.** The electrophilicity and nucleophilicity index of various organic molecules.

| **Organic** | **Electrophilicity index (eV)** | **Nucleophilicity index (eV)** |
| --- | --- | --- |
| TC | 1.2912 | 2.7314 |
| OTC | 1.3386 | 2.8113 |
| CTC | 1.3361 | 2.7006 |
| SMX | 0.9359 | 2.5238 |
| SIX | 0.9421 | 2.6725 |
| SDZ | 3.8645 | 3.1464 |
| PN | 0.7598 | 2.2550 |
| AN | 0.7127 | 2.2044 |
| DCP | 0.8928 | 2.3062 |
| CIP | 1.0468 | 3.0038 |
| OFX | 0.9483 | 3.4239 |
| BPA | 0.7286 | 3.1588 |
| BPS | 1.0387 | 2.3866 |
| CBZ | 1.0657 | 2.9147 |
| ATZ | 0.8520 | 2.3769 |
| BA | 1.1913 | 1.5059 |
| NBA | 2.0615 | 0.7815 |

**Table S6.** The Fukui index and NPA charge distribution of different atoms in TC.

| **No.** | **Atom** | **Charge(0)**  **(e/Å^3^)** | **Charge(+1)**  **(e/Å^3^)** | **Charge(-1)**  **(e/Å^3^)** | ***f* ^－^** | ***f* ^+^** | ***f* ^0^** |
| --- | --- | --- | --- | --- | --- | --- | --- |
| 1 | O | -0.2325 | -0.2588 | -0.2133 | 0.0193 | 0.0263 | 0.0228 |
| 2 | O | -0.2121 | -0.2226 | -0.2023 | 0.0098 | 0.0105 | 0.0101 |
| 3 | O | -0.1716 | -0.1902 | -0.1259 | 0.0458 | 0.0186 | 0.0322 |
| 4 | O | -0.2944 | -0.3179 | -0.2750 | 0.0194 | 0.0235 | 0.0214 |
| 5 | N | -0.0923 | -0.0924 | -0.0253 | 0.0670 | 0.0001 | 0.0335 |
| 6 | C | -0.0196 | -0.0221 | -0.0186 | 0.0010 | 0.0025 | 0.0017 |
| 7 | C | -0.0264 | -0.0294 | -0.0183 | 0.0081 | 0.0030 | 0.0055 |
| 8 | C | -0.0564 | -0.0592 | -0.0487 | 0.0078 | 0.0028 | 0.0053 |
| 9 | C | 0.0732 | 0.0648 | 0.0736 | 0.0004 | 0.0084 | 0.0044 |
| 10 | C | 0.0219 | 0.0177 | 0.0294 | 0.0074 | 0.0042 | 0.0058 |
| 11 | C | 0.0898 | 0.0882 | 0.0905 | 0.0007 | 0.0016 | 0.0012 |
| 12 | C | -0.0637 | -0.0813 | 0.0005 | 0.0643 | 0.0175 | 0.0409 |
| 13 | C | 0.0979 | 0.0509 | 0.1360 | 0.0381 | 0.0469 | 0.0425 |
| 14 | C | 0.1388 | 0.1083 | 0.1358 | -0.0030 | 0.0305 | 0.0138 |
| 15 | C | 0.0081 | -0.0172 | 0.0156 | 0.0075 | 0.0253 | 0.0164 |
| 16 | C | 0.1273 | 0.0872 | 0.1287 | 0.0014 | 0.0401 | 0.0207 |
| 17 | C | 0.1279 | 0.0676 | 0.1392 | 0.0113 | 0.0604 | 0.0358 |
| 18 | C | -0.0776 | -0.0881 | -0.0662 | 0.0114 | 0.0104 | 0.0109 |
| 19 | C | -0.0332 | -0.0462 | -0.0119 | 0.0213 | 0.0130 | 0.0172 |
| 20 | C | -0.0905 | -0.0981 | -0.0851 | 0.0054 | 0.0077 | 0.0065 |
| 21 | C | -0.0573 | -0.0797 | -0.0153 | 0.0419 | 0.0224 | 0.0322 |
| 22 | C | -0.0393 | -0.0453 | -0.0232 | 0.0160 | 0.0060 | 0.0110 |
| 23 | C | -0.0427 | -0.0480 | -0.0268 | 0.0159 | 0.0053 | 0.0106 |
| 24 | C | 0.0914 | 0.0666 | 0.1227 | 0.0314 | 0.0247 | 0.0281 |
| 25 | C | 0.1650 | 0.1575 | 0.1680 | 0.0030 | 0.0076 | 0.0053 |
| 26 | C | -0.0285 | -0.0835 | 0.0068 | 0.0353 | 0.0550 | 0.0451 |
| 27 | C | -0.0716 | -0.1005 | -0.0436 | 0.0279 | 0.0290 | 0.0284 |
| 48 | O | -0.1734 | -0.2155 | -0.1189 | 0.0545 | 0.0421 | 0.0483 |
| 50 | O | -0.1577 | -0.1946 | -0.1388 | 0.0189 | 0.0369 | 0.0279 |
| 52 | N | -0.1340 | -0.1480 | -0.1213 | 0.0127 | 0.0141 | 0.0134 |
| 55 | O | -0.2368 | -0.2860 | -0.2126 | 0.0243 | 0.0491 | 0.0367 |
| 56 | O | -0.2453 | -0.3156 | -0.2107 | 0.0346 | 0.0703 | 0.0524 |

**Table S7.** Input materials and energy required to treat 1 ton of TC wastewater based on batch experiments through different systems.

| **Treatment of 1 ton of TC wastewater** | **PKU-24/PMS system** | **Money ($)** | **ZIF-67/PMS system** | **Money ($)** | **MIL-88A/PMS system** | **Money ($)** |
| --- | --- | --- | --- | --- | --- | --- |
| Co(NO_3_)_3_·6H_2_O (g) | 83.15 | 3.15 | 484.95 | 22.99 |  |  |
| FeCl_3_·6H_2_O (g) |  |  |  |  | 300.4 | 45.92 |
| 2-hydroxyterephthalic acid (g) | 52.06 | 68.05 |  |  |  |  |
| 2-methylimidazole (g) |  |  | 547.34 | 18.12 |  |  |
| Fumaric acid (g) |  |  |  |  | 128.88 | 9.76 |
| 4, 4-bipyridine (g) | 44.619 | 14.71 |  |  |  |  |
| Methanol (L) |  |  | 106.656 | 329.09 |  |  |
| Ethyl alcohol (L) |  |  |  |  | 11.1 | 40.48 |
| H_2_O (L) | 9.523 | 2.14 |  |  |  |  |
| DMF (L) | 0.9523 | 5.07 |  |  | 22.22 | 118.32 |
| Drying oven (kWh) | 142.8 | 13.02 | 166.65 | 15.19 | 199.98 | 18.23 |
| Centrifuge (kWh) |  |  | 11.11 | 1.01 | 11.11 | 1.01 |
| Suction filtration (kWh) | 3.8 | 0.35 |  |  |  |  |
| Stirrer (kWh) | 16 | 1.46 | 655.9 | 58.34 | 16 | 1.46 |
| PMS (g) | 122.94 | 4.13 | 122.94 | 4.13 | 122.94 | 4.13 |

**Table S8.** Impact assessment for the treatment of 1 ton TC wastewater (ZIF-67/PMS system).

| **Impact category** | **Unit** | **Total** | **Co(NO_3_)_2_·6H_2_O (g)** | **2-methylimidazole** | | **Methanol** | | **PMS** | | **Drying oven** | | **Centrifuge** | | **Stirrer** | |
| --- | --- | --- | --- | --- | --- | --- | --- | --- | --- | --- | --- | --- | --- | --- | --- |
|  |  |  | **Cobalt(III) nitrate \| market for cobalt(III) nitrate \| Cut-off, S** | **N-methylimidazole {GLO}\| market for \| Cut-off, S** | **Methanol {GLO}\| market for \| Cut-off, S** | | **Sodium persulfate {GLO}\| market for \| Cut-off, S** | | **Electricity, high voltage {CN}\| market group for \| Cut-off, S** | | **Electricity, high voltage {CN}\| market group for \| Cut-off, S** | | **Electricity, high voltage {CN}\| market group for \| Cut-off, S** | |  |
| Global warming | kg CO_2_ eq | 883.649 | 11.64172 | 3.028993 | | 0.074386 | | 0.003843 | | 173.6946 | | 11.57964 | | 683.6259 | |
| Stratospheric ozone depletion | kg CFC11 eq | 0.000194 | 1.12E-05 | 6.66E-07 | | 3.95E-08 | | 2.25E-09 | | 3.63E-05 | | 2.42E-06 | | 0.000143 | |
| Ionizing radiation | kBq Co-60 eq | 17.12597 | 3.715632 | 0.15658 | | 0.001163 | | 0.000315 | | 2.649152 | | 0.17661 | | 10.42652 | |
| Ozone formation, Human health | kg NO_x_ eq | 2.447383 | 0.026979 | 0.005412 | | 0.000149 | | 1.04E-05 | | 0.482729 | | 0.032182 | | 1.899922 | |
| Fine particulate matter formation | kg PM2.5 eq | 1.363371 | 0.032599 | 0.003655 | | 7.03E-05 | | 1.15E-05 | | 0.265277 | | 0.017685 | | 1.044074 | |
| Ozone formation, Terrestrial ecosystems | kg NO_x_ eq | 2.453361 | 0.027578 | 0.005679 | | 0.000159 | | 1.07E-05 | | 0.483749 | | 0.03225 | | 1.903936 | |
| Terrestrial acidification | kg SO_2_ eq | 3.059008 | 0.091194 | 0.008921 | | 0.000194 | | 2.82E-05 | | 0.591443 | | 0.03943 | | 2.327798 | |
| Freshwater eutrophication | kg P eq | 0.168097 | 0.006784 | 0.001041 | | 1.09E-05 | | 2.51E-06 | | 0.032036 | | 0.002136 | | 0.126086 | |
| Marine eutrophication | kg N eq | 0.013767 | 0.0019 | 0.00164 | | 6.73E-07 | | 1.75E-07 | | 0.002044 | | 0.000136 | | 0.008046 | |
| Terrestrial ecotoxicity | kg 1,4-DCB | 1019.581 | 533.8495 | 8.327432 | | 0.093842 | | 0.068116 | | 95.40157 | | 6.360105 | | 375.4809 | |
| Freshwater ecotoxicity | kg 1,4-DCB | 10.89789 | 2.3166 | 0.098378 | | 0.001101 | | 0.000661 | | 1.695396 | | 0.113026 | | 6.672729 | |
| Marine ecotoxicity | kg 1,4-DCB | 15.00256 | 3.129091 | 0.128777 | | 0.001525 | | 0.000867 | | 2.347306 | | 0.156487 | | 9.238511 | |
| Human carcinogenic toxicity | kg 1,4-DCB | 27.76163 | 1.215565 | 0.154264 | | 0.001833 | | 0.000408 | | 5.275317 | | 0.351688 | | 20.76256 | |
| Human non-carcinogenic toxicity | kg 1,4-DCB | 453.4418 | 66.28687 | 2.085608 | | 0.023251 | | 0.012987 | | 76.96874 | | 5.13125 | | 302.9331 | |
| Land use | m^2^a crop eq | 10.85374 | 0.43501 | 0.049215 | | 0.001341 | | 0.000116 | | 2.072593 | | 0.138173 | | 8.157296 | |
| Mineral resource scarcity | kg Cu eq | 4.592288 | 4.317523 | 0.007189 | | 0.000114 | | 4.27E-05 | | 0.053458 | | 0.003564 | | 0.210398 | |
| Fossil resource scarcity | kg oil eq | 174.6121 | 3.923068 | 1.257445 | | 0.081194 | | 0.001121 | | 33.85319 | | 2.256879 | | 133.2392 | |
| Water consumption | m^3^ | 4.799807 | 2.600057 | 0.048845 | | 0.000479 | | 0.000128 | | 0.429848 | | 0.028657 | | 1.691793 | |

| Calculation: | Analyze |
| --- | --- |
| Results: | Impact assessment |
| Product: | Treatment of 1 ton TC wastewater (ZIF-67/PMS system). |
| Method: | ReCiPe 2016 Midpoint (H) V1.07 / World (2010) H |
| Indicator: | Characterization |
| Skip categories: | Never |
| Exclude infrastructure processes: | No |
| Exclude long-term emissions: | No |
| Sorted on item: | Impact category |
| Sort order: | Ascending |

**Table S9.** Impact assessment for the treatment of 1 ton TC wastewater (MIL-88A/PMS system).

| **Impact category** | **Unit** | **Total** | **FeCl_3_·6H_2_O** | **Fumaric acid** | | **Ethyl alcohol** | | **PMS** | | **DMF** | | **Drying oven** | | **Centrifuge** | |
| --- | --- | --- | --- | --- | --- | --- | --- | --- | --- | --- | --- | --- | --- | --- | --- |
|  |  |  | **Iron (III) chloride, without water, in 40% solution state {GLO}\| market for \| Cut-off, S** | **Fumaric acid {GLO}\| market for fumaric acid \| Cut-off, S** | **Ethanol, without water, in 95% solution state, from fermentation [35]\| market for ethanol, without water, in 95% solution state, from fermentation \| Cut-off, S** | | **Sodium persulfate {GLO}\| market for \| Cut-off, S** | | **N,N-dimethylformamide {GLO}\| market for \| Cut-off, S** | | **Electricity, high voltage {CN}\| market group for \| Cut-off, S** | | **Electricity, high voltage {CN}\| market group for \| Cut-off, S** | |  |
| Global warming | kg CO_2_ eq | 284.3573 | 0.242957 | 0.421694 | | 0.009011 | | 0.003843 | | 63.66667 | | 208.4335 | | 11.57964 | |
| Stratospheric ozone depletion | kg CFC11 eq | 7.07E-05 | 2.16E-07 | 1.42E-07 | | 5.02E-08 | | 2.25E-09 | | 2.43E-05 | | 4.36E-05 | | 2.42E-06 | |
| Ionizing radiation | kBq Co-60 eq | 7.201552 | 0.02007 | 0.021859 | | 0.000124 | | 0.000315 | | 3.803592 | | 3.178982 | | 0.17661 | |
| Ozone formation, Human health | kg NO_x_ eq | 0.739775 | 0.000706 | 0.000762 | | 2.71E-05 | | 1.04E-05 | | 0.126812 | | 0.579275 | | 0.032182 | |
| Fine particulate matter formation | kg PM2.5 eq | 0.425038 | 0.00061 | 0.000537 | | 1.93E-05 | | 1.15E-05 | | 0.087844 | | 0.318332 | | 0.017685 | |
| Ozone formation, Terrestrial ecosystems | kg NO_x_ eq | 0.746557 | 0.000716 | 0.0008 | | 2.82E-05 | | 1.07E-05 | | 0.132254 | | 0.580499 | | 0.03225 | |
| Terrestrial acidification | kg SO_2_ eq | 0.963143 | 0.001161 | 0.001202 | | 8.15E-05 | | 2.82E-05 | | 0.211509 | | 0.709732 | | 0.03943 | |
| Freshwater eutrophication | kg P eq | 0.076128 | 0.000153 | 0.000131 | | 2.93E-06 | | 2.51E-06 | | 0.035261 | | 0.038443 | | 0.002136 | |
| Marine eutrophication | kg N eq | 0.066621 | 1.09E-05 | 5.83E-06 | | 1.16E-05 | | 1.75E-07 | | 0.064003 | | 0.002453 | | 0.000136 | |
| Terrestrial ecotoxicity | kg 1,4-DCB | 354.4227 | 3.033878 | 1.138243 | | 0.028681 | | 0.068116 | | 229.3118 | | 114.4819 | | 6.360105 | |
| Freshwater ecotoxicity | kg 1,4-DCB | 4.702647 | 0.035394 | 0.011401 | | 0.000675 | | 0.000661 | | 2.507014 | | 2.034475 | | 0.113026 | |
| Marine ecotoxicity | kg 1,4-DCB | 6.272704 | 0.046054 | 0.015179 | | 0.000452 | | 0.000867 | | 3.236899 | | 2.816767 | | 0.156487 | |
| Human carcinogenic toxicity | kg 1,4-DCB | 9.591013 | 0.031791 | 0.014422 | | 0.000381 | | 0.000408 | | 2.861944 | | 6.33038 | | 0.351688 | |
| Human non-carcinogenic toxicity | kg 1,4-DCB | 149.045 | 0.621379 | 0.240404 | | 0.014336 | | 0.012987 | | 50.66217 | | 92.36249 | | 5.13125 | |
| Land use | m^2^a crop eq | 4.260427 | 0.010202 | 0.008668 | | 0.01285 | | 0.000116 | | 1.603308 | | 2.487111 | | 0.138173 | |
| Mineral resource scarcity | kg Cu eq | 0.257604 | 0.00268 | 0.000834 | | 4.10E-05 | | 4.27E-05 | | 0.186294 | | 0.064149 | | 0.003564 | |
| Fossil resource scarcity | kg oil eq | 76.64982 | 0.060373 | 0.191883 | | 0.00131 | | 0.001121 | | 33.51442 | | 40.62383 | | 2.256879 | |
| Water consumption | m^3^ | 1.354403 | 0.005132 | 0.002651 | | 0.001507 | | 0.000128 | | 0.80051 | | 0.515818 | | 0.028657 | |

| Calculation: | Analyze |
| --- | --- |
| Results: | Impact assessment |
| Product: | Treatment of 1 ton TC wastewater (MIL-88A/PMS system). |
| Method: | ReCiPe 2016 Midpoint (H) V1.07 / World (2010) H |
| Indicator: | Characterization |
| Skip categories: | Never |
| Exclude infrastructure processes: | No |
| Exclude long-term emissions: | No |
| Sorted on item: | Impact category |
| Sort order: | Ascending |

**Table S10.** Impact assessment for the treatment of 1 ton TC wastewater (PKU-24/PMS system).

| **Impact category** | **Unit** | **Total** | **Co(NO_3_)_2_·6H_2_O (g)** | **2-hydroxyterephthalic acid** | **4, 4-bipyridine** | **PMS** | **H_2_O** | **DMF** | **Drying oven** | **Suction filtration** | **Stirrer** |
| --- | --- | --- | --- | --- | --- | --- | --- | --- | --- | --- | --- |
|  |  |  | **Cobalt(III) nitrate [35]\| market for cobalt(III) nitrate \| Cut-off, S** | **Hydroxyterephthalic acid {GLO}\| market for \| Cut-off, S** | **Pyridine-compound {GLO}\| market for \| Cut-off, S** | **Sodium persulfate {GLO}\| market for \| Cut-off, S** | **Water, deionised [35]\| market for water, deionised \| Cut-off, S** | **N,N-dimethylformamide {GLO}\| market for \| Cut-off, S** | **Electricity, high voltage {CN}\| market group for \| Cut-off, S** | **Electricity, high voltage {CN}\| market group for \| Cut-off, S** | **Electricity, high voltage {CN}\| market group for \| Cut-off, S** |
| Global warming | kg CO_2_ eq | 174.8613 | 1.9961 | 0.178869 | 0.47784 | 0.001922 | 0.004572 | 2.728612 | 148.8364 | 3.960632 | 16.67635 |
| Stratospheric ozone depletion | kg CFC11 eq | 3.93E-05 | 1.92E-06 | 6.21E-07 | 2.58E-07 | 1.13E-09 | 4.30E-09 | 1.04E-06 | 3.11E-05 | 8.28E-07 | 3.49E-06 |
| Ionizing radiation | kBq Co-60 eq | 3.428333 | 0.637086 | 0.008189 | 0.034808 | 0.000157 | 0.000308 | 0.163014 | 2.27002 | 0.060407 | 0.254344 |
| Ozone formation, Human health | kg NO_x_ eq | 0.482519 | 0.004626 | 0.00038 | 0.001065 | 5.22E-06 | 1.07E-05 | 0.005435 | 0.413644 | 0.011007 | 0.046347 |
| Fine particulate matter formation | kg PM2.5 eq | 0.269355 | 0.005589 | 0.000315 | 0.000838 | 5.77E-06 | 1.18E-05 | 0.003765 | 0.227312 | 0.006049 | 0.025469 |
| Ozone formation, Terrestrial ecosystems | kg NO_x_ eq | 0.483918 | 0.004728 | 0.000404 | 0.001109 | 5.33E-06 | 1.08E-05 | 0.005668 | 0.414518 | 0.011031 | 0.046445 |
| Terrestrial acidification | kg SO_2_ eq | 0.604283 | 0.015636 | 0.000779 | 0.001691 | 1.41E-05 | 2.81E-05 | 0.009065 | 0.506799 | 0.013486 | 0.056784 |
| Freshwater eutrophication | kg P eq | 0.03425 | 0.001163 | 0.0001 | 0.000214 | 1.25E-06 | 2.53E-06 | 0.001511 | 0.027451 | 0.00073 | 0.003076 |
| Marine eutrophication | kg N eq | 0.005118 | 0.000326 | 4.61E-06 | 4.94E-05 | 8.77E-08 | 1.86E-07 | 0.002743 | 0.001752 | 4.66E-05 | 0.000196 |
| Terrestrial ecotoxicity | kg 1,4-DCB | 199.9542 | 91.53435 | 1.589733 | 3.819785 | 0.034058 | 0.065434 | 9.827797 | 81.74824 | 2.175373 | 9.159467 |
| Freshwater ecotoxicity | kg 1,4-DCB | 2.21548 | 0.397206 | 0.014643 | 0.041007 | 0.000331 | 0.000654 | 0.107445 | 1.452761 | 0.038659 | 0.162774 |
| Marine ecotoxicity | kg 1,4-DCB | 3.019495 | 0.536517 | 0.018935 | 0.033766 | 0.000433 | 0.000856 | 0.138726 | 2.011373 | 0.053524 | 0.225364 |
| Human carcinogenic toxicity | kg 1,4-DCB | 5.516732 | 0.208422 | 0.009687 | 0.028091 | 0.000204 | 0.000557 | 0.122657 | 4.520344 | 0.120289 | 0.506481 |
| Human non-carcinogenic toxicity | kg 1,4-DCB | 89.4742 | 11.36561 | 0.285403 | 0.53425 | 0.006494 | 0.01296 | 2.171269 | 65.95341 | 1.755063 | 7.389738 |
| Land use | m^2^a crop eq | 2.180658 | 0.074587 | 0.003071 | 0.011876 | 5.80E-05 | 0.000127 | 0.068714 | 1.775975 | 0.04726 | 0.198989 |
| Mineral resource scarcity | kg Cu eq | 0.803267 | 0.740287 | 0.001054 | 0.001707 | 2.14E-05 | 5.55E-05 | 0.007984 | 0.045807 | 0.001219 | 0.005132 |
| Fossil resource scarcity | kg oil eq | 35.37246 | 0.672653 | 0.081654 | 0.149609 | 0.000561 | 0.001154 | 1.436354 | 29.00832 | 0.77193 | 3.250231 |
| Water consumption | m^3^ | 0.923763 | 0.445808 | 0.003008 | 0.011208 | 6.40E-05 | 0.009964 | 0.034308 | 0.368331 | 0.009802 | 0.04127 |

| Calculation: | Analyze |
| --- | --- |
| Results: | Impact assessment |
| Product: | Treatment of 1 ton TC wastewater (PKU-24/PMS system). |
| Method: | ReCiPe 2016 Midpoint (H) V1.07 / World (2010) H |
| Indicator: | Characterization |
| Skip categories: | Never |
| Exclude infrastructure processes: | No |
| Exclude long-term emissions: | No |
| Sorted on item: | Impact category |
| Sort order: | Ascending |

**References**

[1] X.-H. Yi, H. Ji, C.-C. Wang, Y. Li, Y.-H. Li, C. Zhao, A. Wang, H. Fu, P. Wang and X. Zhao, “Photocatalysis-activated SR-AOP over PDINH/MIL-88A(Fe) composites for boosted chloroquine phosphate degradation: Performance, mechanism, pathway and DFT calculations,” *Appl. Catal. B* 293 (2021): 120229.

[2] K.-X. Shi, F. Qiu, J.-W. Wang, P. Wang, H.-Y. Li and C.-C. Wang, “Sulfamethoxazole degradation via peroxydisulfate activation over WO_3_/MIL-100(Fe) under low power LED visible light,” *Sep. Purif. Technol.* 309 (2023): 122991.

[3] F.-X. Wang, Z.-C. Zhang, X.-H. Yi, C.-C. Wang, P. Wang, C.-Y. Wang and B. Yu, “A micron-sized Co-MOF sheet to activate peroxymonosulfate for efficient organic pollutant degradation,” *CrystEngComm* 24 (2022): 5557-5561.

[4] L. Dai, J. Xu, J. Lin, L. Wu, H. Cai, J. Zou and J. Ma, “Iodometric spectrophotometric determination of peroxydisulfate in hydroxylamine-involved AOPs: 15 min or 15 s for oxidative coloration?,” *Chemosphere* 272 (2021): 128577.

[5] C. Liang, C.F. Huang, N. Mohanty and R.M. Kurakalva, “A rapid spectrophotometric determination of persulfate anion in ISCO,” *Chemosphere* 73 (2008): 1540-1543.

[6] S. Plimpton, “Fast parallel algorithms for short-range molecular dynamics,” *J. Comput. Phys.* 117 (1995): 1-19.

[7] A. Stukowski, “Visualization and analysis of atomistic simulation data with OVITO–the Open Visualization Tool,” *Model. Simul. Mater. Sci. Eng.* 18 (2009): 015012.

[8] W.L. Jorgensen, D.S. Maxwell and J. Tirado-Rives, “Development and testing of the OPLS all-atom force field on conformational energetics and properties of organic liquids,” *J. Am. Chem. Soc.* 118 (1996): 11225-11236.

[9] S. Nosé, “A unified formulation of the constant temperature molecular dynamics methods,” *J. Chem. Phys.* 81 (1984): 511-519.

[10] W.G. Hoover, “Canonical dynamics: Equilibrium phase-space distributions,” *Phys. Rev. A* 31 (1985): 1695.

[11] U. Essmann, L. Perera, M.L. Berkowitz, T. Darden, H. Lee and L.G. Pedersen, “A smooth particle mesh Ewald method,” *J. Chem. Phys.* 103 (1995): 8577-8593.

[12] A. Wang, Z. Zheng, H. Wang, Y. Chen, C. Luo, D. Liang, B. Hu, R. Qiu and K. Yan, “3D hierarchical H_2_-reduced Mn-doped CeO_2_ microflowers assembled from nanotubes as a high-performance Fenton-like photocatalyst for tetracycline antibiotics degradation,” *Appl. Catal. B* 277 (2020): 119171.

[13] X. Zhang, B. Xu, S. Wang, X. Li, C. Wang, B. Liu, F. Han, Y. Xu, P. Yu and Y. Sun, “Tetracycline degradation by peroxymonosulfate activated with CoN_x_ active sites: Performance and activation mechanism,” *Chem. Eng. J.* 431 (2022): 133477.

[14] N. Tian, X. Tian, Y. Nie, C. Yang, Z. Zhou and Y. Li, “Biogenic manganese oxide: An efficient peroxymonosulfate activation catalyst for tetracycline and phenol degradation in water,” *Chem. Eng. J.* 352 (2018): 469-476.

[15] X. Ren, Y. Wang, G. Hu, Q. Guo, D. Gao, P. Huang, W. Zhang, L. Wang and X. Hu, “Improved PMS activation by natural sepiolite/Fe_3_O_4_ composite for effective tetracycline degradation: Performance, mechanism and degradation pathway,” *J. Environ. Chem. Eng.* 12 (2024): 111878.

[16] M. Xiong, J. Yan, G. Fan, Y. Liu, B. Chai, C. Wang and G. Song, “Built-in electric field mediated peroxymonosulfate activation over biochar supported-Co_3_O_4_ catalyst for tetracycline hydrochloride degradation,” *Chem. Eng. J.* 444 (2022): 136589.

[17] C. Wang, N. Liu, X. Liu, Y. Tian, H. Xie, Y. Zhang, X. Chen and B. Hou, “Photo-Fenton-like degradation of tetracycline through peroxymonosulfate activation over 2D BiOBr/FeOOH nanosheets and membrane application,” *Chem. Eng. J.* 491 (2024): 151993.

[18] C. Jin, J. Kang, Z. Li, M. Wang, Z. Wu and Y. Xie, “Enhanced visible light photocatalytic degradation of tetracycline by MoS_2_/Ag/g-C_3_N_4_ Z-scheme composites with peroxymonosulfate,” *Appl. Surf. Sci.* 514 (2020): 146076.

[19] H. Shi, Y. He, Y. Li and P. Luo, “Unraveling the synergy mechanism between photocatalysis and peroxymonosulfate activation on a Co/Fe bimetal-doped carbon nitride,” *ACS Catal.* 13 (2023): 8973-8986.

[20] T. Guo, L. Jiang, H. Huang, Y. Li, X. Wu and G. Zhang, “Enhanced degradation of tetracycline in water over Cu-doped hematite nanoplates by peroxymonosulfate activation under visible light irradiation,” *J. Hazard. Mater.* 416 (2021): 125838.

[21] J. Jiang, X. Wang, C. Yue, S. Liu, Y. Lin, T. Xie and S. Dong, “Efficient photoactivation of peroxymonosulfate by Z-scheme nitrogen-defect-rich NiCo_2_O_4_/g-C_3_N_4_ for rapid emerging pollutants degradation,” *J. Hazard. Mater.* 414 (2021): 125528.

[22] Y. Tang, J. Kang, M. Wang, C. Jin, J. Liu, M. Li, S. Li and Z. Li, “Catalytic degradation of oxytetracycline via FeVO_4_ nanorods activating PMS and the insights into the performance and mechanism,” *J. Environ. Chem. Eng.* 9 (2021): 105864.

[23] X. Di, X. Zeng, T. Tang, D. Liu, Y. Shi, W. Wang, Z. Liu, L. Jin, X. Ji and X. Shao, “Non-radical activation of peroxymonosulfate by modified activated carbon for efficient degradation of oxytetracycline: Mechanisms and applications,” *Sep. Purif. Technol.* 349 (2024): 127877.

[24] H. Ji, Y. Xu, H. Shi and X. Yang, “Enhanced activation of persulfate by magnetic NiFe_2_O_4_@CuO coupled with ultrasonic for degradation of oxytetracycline: Activation mechanism and degradation pathway,” *Appl. Surf. Sci.* 652 (2024): 159373.

[25] S. Wang, J. Hu and J. Wang, “Degradation of sulfamethoxazole using PMS activated by cobalt sulfides encapsulated in nitrogen and sulfur co-doped graphene,” *Sci. Total Environ.* 827 (2022): 154379.

[26] S. Wang and J. Wang, “High efficient activation of peroxymonosulfate by Co_9_S_8_ anchored in N, S, O co-doped carbon composite for degradation of sulfamethoxazole: effect of sulfur precursor and sulfur doping content,” *Chem. Eng. J.* 434 (2022): 134824.

[27] J. He, J. Huang, Z. Wang, Z. Liu, Y. Chen, R. Su, X. Ni, Y. Li, X. Xu and W. Zhou, “The enhanced catalytic degradation of sulfamethoxazole over Fe@nitrogen-doped carbon-supported nanocomposite: Insight into the mechanism,” *Chem. Eng. J.* 439 (2022): 135784.

[28] Y. Wang, W. Peng, J. Wang, G. Chen, N. Li, Y. Song, Z. Cheng, B. Yan, L.a. Hou and S. Wang, “Sulfamethoxazole degradation by regulating active sites on distilled spirits lees-derived biochar in a continuous flow fixed bed peroxymonosulfate reactor,” *Appl. Catal. B* 310 (2022): 121342.

[29] J. Li, X. Jiang, H. Guan, Z. Liu, J. Li, Z. Lin, F. Li and W. Xu, “Visible-light-driven peroxymonosulfate activation by robust TiO_2_-base nanoparticles for efficient removal of sulfamethoxazole,” *Environ. Pollut.* 334 (2023): 122150.

[30] X. Lv, M. Yu, Y. Guo and M. Sui, “Efficient heterogeneous activation of peroxymonosulfate by Ag-doped CoFe_2_O_4_ nanoparticles for sulfamethoxazole degradation,” *J. Environ. Chem. Eng.* 11 (2023): 110980.

[31] L. Xie, J. Hao, Y. Wu and S. Xing, “Non-radical activation of peroxymonosulfate with oxygen vacancy-rich amorphous MnOX for removing sulfamethoxazole in water,” *Chem. Eng. J.* 436 (2022): 135260.

[32] H. Zhang, R.L. Smith, H. Guo and X. Qi, “Cobalt cross-linked ordered mesoporous carbon as peroxymonosulfate activator for sulfamethoxazole degradation,” *Chem. Eng. J.* 472 (2023): 145060.

[33] N. An, S. Li, B. Xu, L. Qian, Y. Shen, K. Wang, X. Li, M. Zhao, X. Zheng and R. Liu, “Role of nitrogen dual reaction sites in N-doped graphene aerogels for synergistic sulfamethoxazole adsorption and peroxymonosulfate activation in Fenton-like process,” *Chem. Eng. J.* 475 (2023): 146309.

[34] X. Wang, Z. Xiong, H. Shi, Z. Wu, B. Huang, H. Zhang, P. Zhou, Z. Pan, W. Liu and B. Lai, “Switching the reaction mechanisms and pollutant degradation routes through active center size-dependent Fenton-like catalysis,” *Appl. Catal. B* 329 (2023): 122569.

[35] J. Krzystek, A. Ozarowski and J. Telser, “Multi-frequency, high-field EPR as a powerful tool to accurately determine zero-field splitting in high-spin transition metal coordination complexes,” *Coord. Chem. Rev.* 250 (2006): 2308-2324.
